# Mutation rate estimate and population genomic analysis reveals decline of koalas prior to human arrival

**DOI:** 10.1093/molbev/msag108

**Published:** 2026-06-09

**Authors:** Toby G L Kovacs, Nicole M Foley, Luke W Silver, Elspeth A McLennan, William J Murphy, Carolyn J Hogg, Simon Y W Ho

**Affiliations:** School of Life and Environmental Sciences, University of Sydney, Sydney, NSW, Australia; Veterinary Integrative Biosciences, Texas A&M University, College Station, TX, USA; School of Life and Environmental Sciences, University of Sydney, Sydney, NSW, Australia; Australian Research Council Centre of Excellence for Innovations in Peptide and Protein Science, The University of Sydney, Sydney, NSW, Australia; School of Life and Environmental Sciences, University of Sydney, Sydney, NSW, Australia; Veterinary Integrative Biosciences, Texas A&M University, College Station, TX, USA; School of Life and Environmental Sciences, University of Sydney, Sydney, NSW, Australia; Australian Research Council Centre of Excellence for Innovations in Peptide and Protein Science, The University of Sydney, Sydney, NSW, Australia; School of Life and Environmental Sciences, University of Sydney, Sydney, NSW, Australia

**Keywords:** mutation rate, historical demographics, population genetics, recombination rate, koala, Australia

## Abstract

The koala (*Phascolarctos cinereus*), an iconic Australian marsupial, has experienced substantial historical and contemporary population declines. Identifying the drivers of these declines has been hindered by limited genomic data and uncertainty regarding the koala mutation rate. Here, we report a direct estimate of the koala mutation rate, based on genome sequences of four parent-offspring trios, yielding a mean of 6.12 × 10^−9^ mutations per base pair per generation (95% confidence interval: 5.03 to 7.45 × 10^−9^). Using this estimate of the mutation rate, we reconstructed the demographic history of koalas using 457 whole-genome sequences sampled across their entire range. Our results refine the estimated timing of past changes in population size, suggesting a large decline beginning ∼100 kya, before the arrival of humans in Australia. The koala population then split into five genetic populations 6 to 30 kya, which are now distributed along the east coast of Australia. We also use our estimate of the mutation rate to infer recombination maps for each koala population, confirming lower recombination rates in marsupials than in eutherian mammals. Using these estimates of population-specific recombination rates, we inferred the timing of recent population declines for koalas across all eastern states. These findings provide critical insights into the evolutionary history of koalas, while highlighting the impacts of using species-specific estimates of evolutionary rates on the inference of demographic histories. Our estimates of the genome-wide mutation rate and population-specific recombination maps for koalas provide valuable resources for future evolutionary and conservation analyses of marsupials.

## Introduction

The Australian continent contains a rich diversity of endemic flora and fauna, which endured severe environmental shifts throughout the late Cenozoic. The continent was dominated by wet forests during the Paleogene but underwent drastic changes during the Miocene as the Australian tectonic plate drifted northwards (Martin 2006). Widespread aridification was marked by the retraction of rainforests to the coasts, the opening of forests, and the expansion of grasslands and deserts (Byrne et al. 2011). Intense glacial and interglacial climate cycles during the Pleistocene drove further shifts toward increasingly arid and fire-prone environments (White 1994). These changes have underpinned the evolution of the Australian biota, driving adaptation to arid environments, the isolation of taxa in remnant mesic habitats, and the extinction of many lineages, especially rainforest specialists (Byrne 2008; Byrne et al. 2008, 2011). More recently, the arrival of modern humans sometime between 65 and 47 kya (O’Connell and Allen 2015; Clarkson et al. 2017; Allen and O’Connell 2025) occurred at a similar time to changes to habitat and fire frequency, and the extinction of many megafaunal species (van der Kaars et al. 2017; Adeleye et al. 2023). However, the sequence and connection between these patterns in Australia is still heavily debated, largely due to the sparsity of the fossil record and uncertainty in the timing of events (Price et al. 2018).

Marsupials are among the most recognizable components of the Australian fauna. These include the koala (*Phascolarctos cinereus*), which is the sole extant member of the family Phascolarctidae. The koala lineage diverged from its closest living relatives, the wombats, ∼36 Mya (Duchêne et al. 2018), with fossil evidence suggesting that the late Oligocene and early Miocene contained a large diversity of koala species across multiple genera (Black et al. 2014; Black 2016; Crichton et al. 2023). More recently, during the Pleistocene, koalas comprised four species across two genera, with the modern koala appearing more than 350 kya and representing the only lineage known to persist into the Holocene (Black et al. 2014). Bioclimate modeling has estimated that koala habitat on the east coast experienced substantial contractions during the Last Glacial Maximum before expanding to its present-day range during the current interglacial period (Adams-Hosking et al. 2011). Contemporary koala populations inhabit an enormous range along the east and south-east coast of Australia but have experienced recent declines as a result of land clearing, disease, hunting, feral dog attacks, vehicle strikes, and bushfires (Menkhorst 2008; Adams-Hosking et al. 2016; Cristescu et al. 2023). Consequently, koalas were listed as Endangered in Queensland (QLD), New South Wales (NSW), and the Australian Capital Territory (ACT) in 2022.

Genetic studies have suggested historically small population sizes in koalas, with museum samples from the 1800s containing low mitochondrial diversity (Tsangaras et al. 2012). Studies of mitochondrial DNA have suggested that population sizes were stable over the last 50,000 years, before expanding after the Last Glacial Maximum (Neaves et al. 2016). Although evidence from exons has pointed to pronounced bottlenecks >300 kya (Lott et al. 2022), whole-genome analyses have suggested that koala populations declined precipitously after modern humans arrived on the continent (Johnson et al. 2018; De Cahsan et al. 2025; Ahrens et al. 2026). This aligns with global patterns of anthropogenic impacts on megafauna, a pattern that has been claimed to extend to Australia (Johnson 2016; van der Kaars et al. 2017; Adeleye et al. 2023). However, the demographic inferences from koala genomes relied on mutation rate estimates from distantly related eutherian mammals (humans and mice), introducing uncertainty in the timing of population-size changes. Therefore, a comprehensive analysis using a more relevant mutation rate can provide a clearer picture of the patterns and drivers of demographic change in koalas.

Mutation rates can be estimated using three main methods: (i) the phylogenetic method, which infers the mutation rate from an estimate of the substitution rate; (ii) the mutation-accumulation method, which requires the sampling of a population for many generations; and (iii) the parent-offspring trio method (Pfeifer 2020). The phylogenetic method has historically been the most widely used, given the relative ease of estimating the substitution rate using molecular-dating methods with fossil calibrations. However, the substitution rate is expected to underestimate the mutation rate, because a proportion of de novo mutations are removed by purifying selection over time (Ho et al. 2011; Yoder and Tiley 2021). Mutation-accumulation and trio-based methods tend to yield broadly similar estimates of mutation rates (Wang and Obbard 2023), but the former method is only feasible for model organisms with short generations.

There has been rapid growth in mutation rate estimates from parent-offspring trios, particularly for vertebrate species (Bergeron et al. 2023; Wang and Obbard 2023). Although many estimates have been obtained for eutherian mammals, there have only been two estimates for marsupial species, each based on a single parent-offspring trio (Tasmanian devil, *Sarcophilus harrisii*; Virginia opossum, *Didelphis virginiana*) (Bergeron et al. 2023). Therefore, obtaining further estimates of mutation rates in Australian marsupials, particularly for the speciose order Diprotodontia (kangaroos, wallabies, possums, wombats, koalas, and others), has the potential to produce substantial improvements in demographic and evolutionary inferences.

Another benefit of obtaining a species-specific mutation rate is accurately inferring other evolutionary parameters, such as recombination rates. Until recently, producing recombination maps was a laborious process that involved genotyping individuals of known pedigree through generations to allow the identification of crossover events between chromosomes (Burbrink et al. 2025). This barrier has now been overcome with the availability of reference-quality long-read assemblies for species of conservation concern (Johnson et al. 2018) and machine-learning approaches that can infer recombination maps from population genomic data (Adrion et al. 2020). These methods have been used to estimate the recombination landscape for a number of non-model species (Bredemeyer et al. 2023; Foley et al. 2024), but their accuracy has been hindered by uncertainty in mutation rates (Adrion et al. 2020).

Recombination rates in marsupials are expected to be lower than those of eutherian mammals, based on their linkage map length (Dumont and Payseur 2008). However, an accurate genomic estimation of the recombination landscape using a relevant mutation rate is required to confirm this. Such an estimate could also improve reconstructions of recent demographic history based on linkage disequilibrium, which rely on an average recombination rate to convert a physical genomic map into a genetic (linkage) map when one is unavailable.

Here we estimate the genomic mutation rate of the modern koala (*P. cinereus*) from four parent-offspring trios and use it to infer demographic history from a large set of whole-genome sequences. We further leverage this mutation rate estimate to generate population-specific recombination maps for koalas and demonstrate their use by inferring recent demographic trajectories. Our study shows that the koala population began to decline before the arrival of modern humans on the Australian continent.

## Results

### Estimation of the koala mutation rate

**Figure 1:**
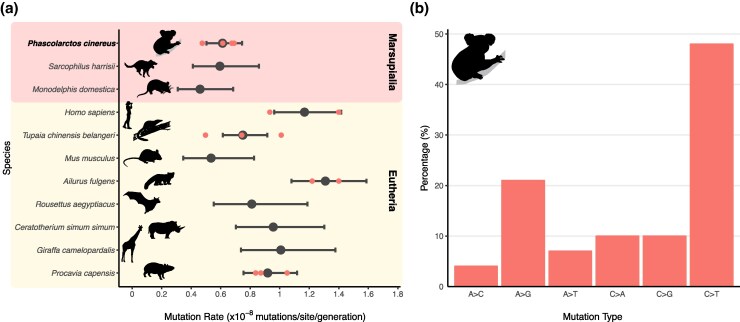
Germline mutation rate of the koala and other mammals. a) Germline mutation rates inferred in the koala (*Phascolarctos cinereus*) based on four parent-offspring trios. Individual estimates for each trio are shown in orange. The average mutation rate is shown in black and 95% confidence intervals are indicated with error bars. Other mutation rate estimates are from Bergeron et al. (2023) and can be found in Table S2. Silhouettes were sourced from Phylopic (http://phylopic.org). b) Mutation spectrum for koala genomes. The percentage of each mutation type is shown after collapsing reverse complements.

We sequenced the genomes of 12 koalas from three families, comprising seven parents and five offspring, with an average coverage of 32× across the samples. The inferred relationships in one trio differed from those recorded in the studbook, and so this trio could not be used to estimate mutation rates. We identified 100 unique de novo mutations in the remaining four offspring after filtering for false discovery following the methods used by Bergeron et al. (2023). We estimated a mean mutation rate of 6.12 × 10^−9^ mutations/bp/generation (95% confidence interval: 5.03 to 7.45 × 10^−9^) across the four trios (Fig. 1a), using the false-negative rate (0.068) and the size of the callable genome (average 68.6% total genome size) (Table S1). The mean estimate can also be expressed as 8.74 × 10^−10^ mutations/bp/year, assuming a generation length of 7 years (Phillips 2000). The detected mutations had a transition to transversion ratio of 2.23 (Fig. 1b).

### Estimation of the population-specific koala recombination maps

**Figure 2:**
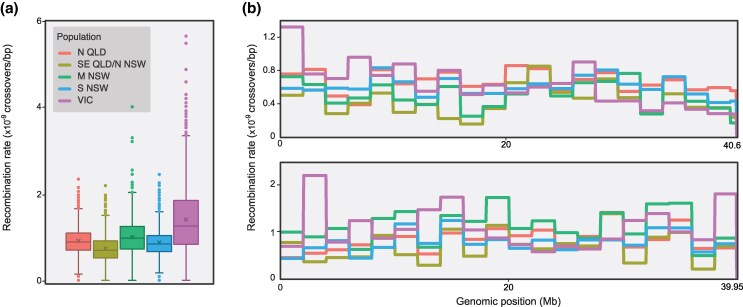
Variation in recombination rates across koala populations. a) Boxplots showing genome-wide recombination rates. In each box, the × and the horizontal line represent the mean and the median recombination rate, respectively. Whiskers extend to the most extreme data points within 1.5 times the interquartile range from the first and third quartiles. Points beyond this are plotted. b) Variation in recombination rates among populations across the two largest scaffolds.

We estimated recombination maps using representatives from each of the five genetic populations identified in the Koala Genome Survey: North Queensland (N QLD), South-East Queensland/North New South Wales (SE QLD/N NSW), Middle New South Wales (M NSW), Southern New South Wales (S NSW), and Victoria (VIC) (Hogg et al. 2023; McLennan et al. 2025; Fig. 4b). We estimated differing recombination rates between populations using ReLERNN (Adrion et al. 2020) (Fig. 2), with the VIC population having the highest mean recombination rate of 1.40 × 10^−9^ crossovers/bp/generation (Q1 − Q3: 0.83 to 1.84 × 10^−9^ crossovers/bp/generation) and the SE QLD/N NSW population with the lowest recombination rate of 0.74 × 10^−9^ crossovers/bp/generation (Q1 − Q3: 0.51 to 0.91 × 10^−9^ crossovers/bp/generation) (Fig. 2; Table S3). The VIC population had a significant difference in recombination rate compared with the four other populations (Mann-Whitney test, all comparisons *P* < 2.2 × 10^−16^) (Fig. 2). The recombination landscape was highly correlated when using either the pan-mammal or koala-specific mutation rate (M NSW rho = 0.74, *P* < 2.2 × 10^−16^; N QLD rho = 0.65, *P* < 2.2 × 10^−16^; VIC rho = 0.66, *P* < 2.2 × 10^−16^). However, the mean recombination rate was significantly higher when using the pan-mammal mutation rate than when using the koala-specific mutation rate for the three populations analyzed (Mann–Whitney test, all comparisons *P* < 2.2 × 10^−16^; Figure S5).

### Estimation of demographic history

**Figure 3:**
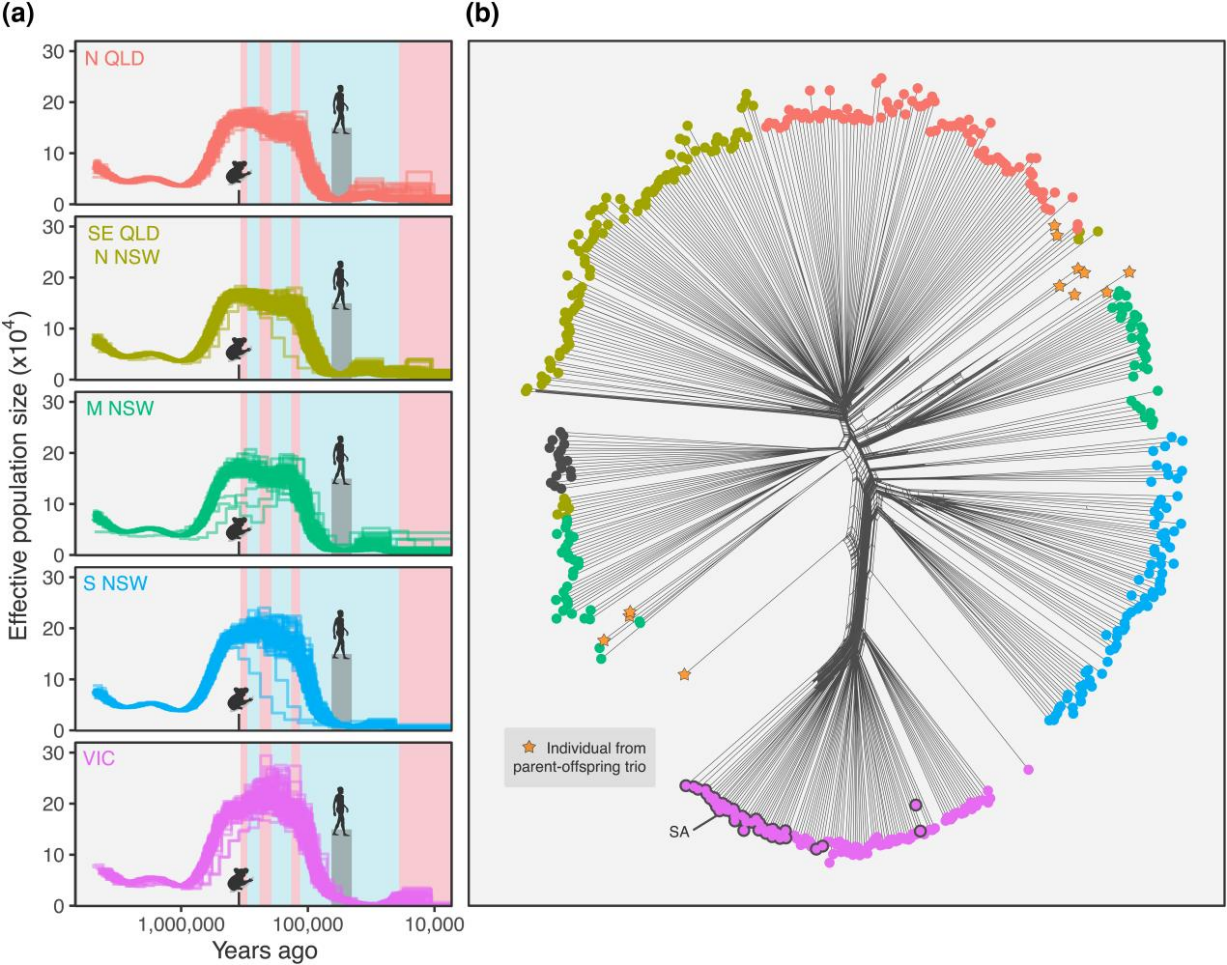
a) Historical effective population sizes estimated using PSMC for 418 individuals across the five genetic populations. Backgrounds indicate the interglacial (warmer and wetter; pink) and glacial (cooler and drier; blue) periods of the last three glacial cycles. The dark gray column and human silhouette indicate the period in which humans are believed to have arrived and spread across Australia (65 to 47 kya; O’Connell and Allen 2015; Clarkson et al. 2017). The koala silhouette indicates the earliest known fossil of the modern koala species (Black et al. 2014). b) Phylogenetic network of all 474 koala samples, including 457 wild and 17 captive koalas, based on Euclidean genetic distances. Colors correspond to the five genetic clusters shown in (a). Individuals that form parent-offspring trios used for mutation-rate estimation are indicated by yellow stars. Koalas from Narrandera, which represent a mix of two populations, are shown in dark gray. Gray outlines highlight koalas from South Australia (SA), which are nested within the Victorian population.

We performed population demographic analyses using 457 genomes of wild koalas, including all 413 samples from the Koala Genome Survey (Hogg et al. 2023; McLennan et al. 2025) and 44 additional samples from Kangaroo Island and the Mount Lofty Ranges (South Australia) to fill geographical gaps. Our phylogenetic network of wild and captive koalas showed a weakly structured population, with samples broadly grouping into the five populations that were recognized previously (McLennan et al. 2025; Fig. 3b). We also show that most of the additional South Australian koalas form a nested clade within the Victorian population. However, a few koalas from the Mount Lofty Ranges were scattered throughout the Victorian clade, suggesting that multiple lineages could be present in South Australia.

We estimated the historical demography of the koala using the pairwise sequentially Markovian coalescent (PSMC; Li and Durbin 2011). Our results suggest that koala populations expanded ∼700 kya and remained stable until ∼120 kya, when they experienced severe and prolonged reductions to reach their minimum effective population size ∼60 kya (Fig. 3a). This pattern showed little variation within and between populations and did not appear to be influenced by the level of sequencing coverage (Figure S1). Our results also suggest that population sizes remained small following this bottleneck, although PSMC is not particularly reliable for inferring demographic trends on recent timeframes (Patton et al. 2019).

When we used the default time intervals designed for analyzing human genomic data, several of our samples produced PSMC plots with recent spikes in population size (Figure S2). These spikes are known artifacts in PSMC analyses and previous studies have shown that they can be removed by subdividing recent time intervals (Hilgers et al. 2025). We also found that, in cases where similar artifacts were produced when grouping the first four intervals (denoted as “4+…”), subdividing the first time interval into two or four intervals (as “2 + 2+…” and “1 + 1 + 1 + 1+…”) sometimes removed these peaks (Figure S2). In some cases, however, when grouping the first four intervals did not introduce an artifact, subdividing the first interval introduced an artificial peak in population size, suggesting that this issue should be assessed on a case-by-case basis.

**Figure 4:**
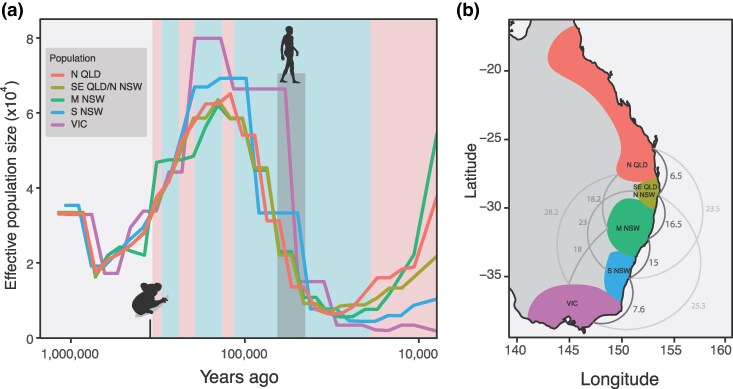
a) Historical population sizes estimated using SMC++ for each of the five genetic groups of koalas. Pink and blue backgrounds indicate the interglacial (warmer and wetter) and glacial (cooler and drier) periods of the last three glacial cycles. The dark gray column and human silhouette indicate the period in which humans are believed to have arrived and spread across Australia. The koala silhouette indicates the earliest known fossil of the modern koala species (Black et al. 2014). b) Population split times (kya) estimated using SMC++ for all pairs of populations. The split times are darker and larger between more closely located populations.

Demographic inference using the sequentially Markovian coalescent can be improved by the addition of multiple samples per population, with SMC++ considered the state-of-the-art method for analyzing samples of more than eight genomes (Terhorst et al. 2017). Our estimates of historical population sizes using SMC++ showed increased resolution, with a larger number of shorter time intervals, and with greater similarity in demographic plots between populations when increasing the number of knots (Figure S3). Our plots using 50 knots showed a similar pattern to that observed in our PSMC results, with koala populations increasing more gradually from 700 to 200 kya before declining between 110 and 40 kya to ∼10% of their maximum population size (Fig. 4a). Following the bottleneck, the three northern populations (N QLD, SE QLD/N NSW, and M NSW) expanded at 15 kya before stabilizing. The South New South Wales population recovered slightly, whereas the Victorian population did not show any signs of recovery.

We also used SMC++ to estimate the split times between pairs of koala populations. Divergences among the populations were estimated to have occurred in the last 30 ky, following a general isolation-by-distance pattern (Fig. 4b; Figure S4). This suggests that the severe bottleneck occurred in the ancestral population of all present-day koalas, with modern lineages subsequently diverging as the population sizes recovered. Based on the pairwise estimates between geographically adjacent populations, we found that the ancestral koala population split at 15 − 16.5 kya to form the three ancestral populations of (i) N QLD and SE QLD/N NSW, (ii) M NSW, and (iii) S NSW and VIC. The two northernmost and two southernmost populations were the last to split, diverging from the remaining populations at 6.5 and 7.6 kya, respectively.

**Figure 5:**
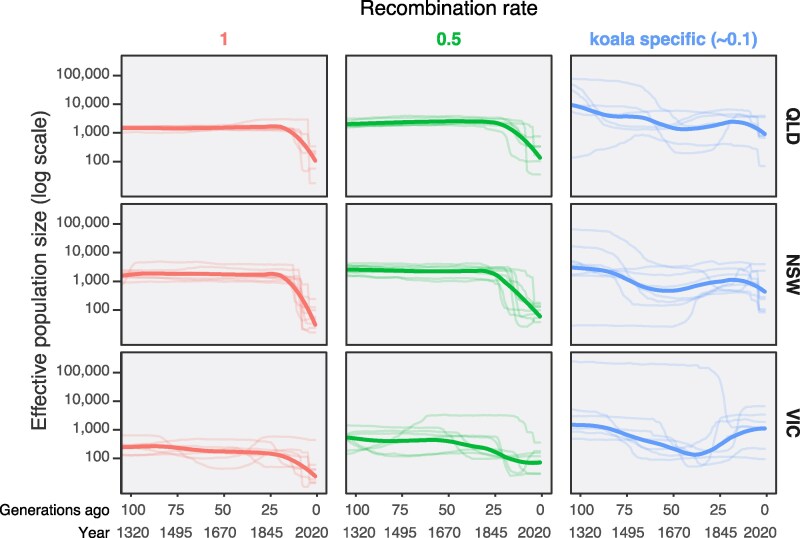
Historical effective population sizes estimated using GoNe2 for 21 local government area (LGA) populations defined by Ahrens et al. (2026). Each population was analyzed under three recombination rate scenarios: 1 cM/Mb (red), 0.5 cM/Mb (green), and the mean recombination rate estimated for the corresponding genetic cluster 0.074 to 0.140 cM/Mb (blue). Populations are grouped by state: Queensland (QLD), New South Wales (NSW), and Victoria (VIC). Individual LGA trajectories are shown as faint lines, while darker lines represent the geometric mean effective population size across populations within each state. One LGA population (South Gippsland), which was inferred to have a very large effective population size, was excluded from the mean calculation for the VIC population.

We estimated demographic histories over the last 100 generations based on linkage disequilibrium using GoNe2 (Santiago et al. 2025; Fig. 5). We used the input files generated by Ahrens et al. (2026), who inferred recent demographic histories for 27 local government area (LGA) populations to minimize the impact of substructure (Ahrens et al. 2026). We analyzed 21 LGA populations after removing three populations that were previously shown to contain individuals from different genetic populations (McLennan et al. 2025) and three populations with three or fewer individuals. In the absence of a genetic map (which measures distances between SNPs in cM), GoNe2 relies on an average recombination rate to interpret a physical map (which measures distances between SNPs in bp). This is the most common approach in analyses of non-model organisms, and has been implemented several times for koalas (de Cahsan et al. 2025; Gates et al. 2025; Ahrens et al. 2026). However, without prior knowledge of the recombination rate in koalas, previous studies have opted to use the default value of 1 cM/Mb (e.g. de Cahsan et al. 2025; Ahrens et al. 2026) or rough estimates from marsupials (e.g. Gates et al. 2025).

Our analyses reveal substantial differences between inferred demographic patterns using our population-specific estimates of koala recombination rates (0.074 to 0.140 cM/Mb; Fig. 2) and the default recombination rate of 1 cM/Mb (Fig. 5). Across all three eastern Australian states, using a recombination rate that is too high (1 or 0.5 cM/Mb) pushed the inferred demographic declines toward the present and did not infer any population recovery. Using the koala-specific recombination rates, we inferred population declines to have occurred on average across all three states (QLD, NSW, and VIC) over the past 75 generations (Fig. 5). Populations in Victoria were found to have experienced the most intense population bottleneck (with the smallest effective population size), but also the largest population recovery post-bottleneck. In the last 10 generations, four of the six QLD populations and six of the eight NSW populations were inferred to have experienced substantial declines, while all populations in Victoria were inferred to be stable (Fig. 5; Figure S6).

## Discussion

### Germline mutation rate in the koala

Our sequencing and analysis of koala genomes from parent-offspring trios has yielded a mutation rate estimate of 6.12 × 10^−9^ mutations/bp/generation (95% confidence interval: 5.03 to 7.45 × 10^−9^), similar to those reported for the Tasmanian devil (5.95 × 10^−9^ mutations/bp/generation) and Virginia opossum (4.60 × 10^−9^ mutations/bp/generation) but considerably lower than that inferred for modern humans (11.6 × 10^−9^ mutations/bp/generation) (Bergeron et al. 2023). Our estimate is lower than a recent estimate of the koala mutation rate using a large pedigree (Neumann et al. 2026), most likely because we filtered de novo mutations for false detection due to the high detection rate of false positives (Bergeron et al. 2023).

The estimated yearly mutation rate of the koala (0.87 × 10^−9^ mutations/bp/year) is lower than those of the Tasmanian devil (1.27 × 10^−9^ mutations/bp/year) and Virginia opossum (2.35 × 10^−9^ mutations/bp/year), due to the shorter generations of these two other marsupials (Lewin and Eyre-Walker 2025). The yearly koala rate is comparable to those of eutherian mammals with similar generation times but higher than that of humans (0.43 × 10^−9^ mutations/bp/year; Lewin and Eyre-Walker 2025). Along with the recent estimate reported by Neumann et al. (2026), our estimate of the koala mutation rate is the first for Diprotodontia, the largest extant marsupial order, providing a valuable resource for evolutionary and demographic analyses of this group.

We obtained similar estimates of mutation rates from genomes sequenced from museum skins and from fresh blood. This result demonstrates that older tissue samples can provide a useful genomic resource with strict site filtering, despite theoretical concerns (Bergeron et al. 2022). Despite a lower average sequencing depth of 32× in our data set compared with the depth of 67× achieved by Bergeron et al. (2023), we recovered a comparable proportion of the genome as callable following the same stringent filtering criteria (69% vs. 72%, respectively). However, our study has also revealed potential risks in relying on studbook records for parental identification. We detected high relatedness between the parents in one trio, suggesting that their founding individuals were likely to be related. This led to a small number of sites for which the parents were homozygous for different alleles, meaning that this trio contributed minimally to the calculation of the false-negative rate.

Compared with previous estimates of mutation rates in marsupials, which only used a single trio each (Bergeron et al. 2023), our analysis of four replicate trios has enabled us to more accurately estimate the distribution of mutations across classes. The distribution across mutation classes for koalas was broadly similar to that observed in primates (Fig. 1b; Versoza et al. 2025), despite >120 My since the divergence between marsupial and eutherian mammals (Álvarez-Carretero et al. 2022) and mutation spectra diverging between some eutherian mammals (Beichman et al. 2023).

Our study provides an estimate of a key evolutionary parameter that can enhance genomic analyses of koalas and other marsupials. Further estimates of mutation rates from a broader range of marsupial species will allow us to test whether the drivers of mutation rates are consistent with those seen in eutherian mammals. Previous studies have reported relationships between the number of de novo mutations and both maternal and paternal age at conception (Jónsson et al. 2017), as well as a paternal bias in mutation contributions (de Manuel et al. 2022). Marsupials could differ in the rate at which mutations accumulate because they give birth after a short gestation period, with most development occurring in the pouch rather than in utero (Smith and Keyte 2020). Teasing apart how de novo mutations arise during this process could help to reveal the broader determinants of mutation rates. Among species, mutation rate variation is correlated with generation time (Bergeron et al. 2023; Zhu et al. 2025), effective population size (Lynch et al. 2016; Zhu et al. 2025), and genome size (Lynch et al. 2016). It is unclear whether these patterns hold in marsupials, but their contrasting developmental strategies make them an interesting test case for evolutionary theories of mutation rate variation in mammals.

### Population-specific koala recombination landscape

Our inferred recombination maps contained mean recombination rates ranging from 0.74 × 10^−9^ to 1.40 × 10^−9^ crossovers/bp/generation, an order of magnitude lower than the average rate in animals of 25.2 × 10^−9^ crossovers/bp/generation (Stapley et al. 2017). However, the estimated recombination rates in koalas are more consistent with the low rates inferred for other marsupials from linkage maps, such as 2.3 × 10^−9^ crossovers/bp/generation in the gray short-tailed opossum (*Monodelphis domestica*) (Samollow et al. 2007, 2010) and 5.1 × 10^−9^ crossovers/bp/generation in the tammar wallaby (*Notamacropus eugenii*) (given a genome length of 3.3 Gb; Wang et al. 2011). Reduced recombination rates in marsupials have been explained by fewer double-stranded breaks during meiosis (Marín-Gual et al. 2022), but the evolutionary drivers remain unknown.

When recombination is reduced, the Hill-Robertson effect reduces the efficacy of selection and the effective population size at linked sites, increasing genetic drift and lowering local levels of diversity (Nordborg et al. 1996; Charlesworth et al. 2009). Therefore, lower recombination rates are expected to further threaten genetically depleted species. We inferred differing recombination rates across the five koala populations, with the highest rate in the VIC population. This result suggests that although the VIC population has the lowest level of genetic diversity due to a recent bottleneck (McLennan et al. 2025), its high rate of genome-wide recombination means that it is likely to experience lower levels of linked selection and more efficient selection. A higher recombination rate could potentially explain how the Victorian population has successfully increased in size following its near extinction in the early 20th century, despite its reduced genetic diversity and high levels of inbreeding (Whisson and Ashman 2020; McLennan et al. 2025).

Previous estimates of recombination maps using ReLERNN in mammal species for which estimates of mutation rates are unavailable (Bredemeyer et al. 2023; Foley et al. 2024) have used a previously inferred “pan-mammal” rate (Kumar and Subramanian 2002). Although this approach enables robust comparison of the recombination landscape within a species, it does not allow direct comparison with other species. Our results confirm the original assessment that ReLERNN produces correlated recombination landscapes for differing mutation rates, but applying an overestimate of the mutation rate, as is the case when applying the “pan-mammal” rate to koalas, results in overestimation of the recombination rates (Adrion et al. 2020). These outcomes highlight another benefit of producing species-specific mutation rates for a diverse range of organisms.

Although our analysis was based on a high-quality, long-read-based genome assembly, it also highlighted some insufficiencies in this genome assembly strategy for the study of the immune loci. Immune genes are innately difficult to assemble because they are highly heterozygous, often evolve through gene duplication, and contain complex structural rearrangements (Logsdon et al. 2025). Although long-read sequencing technologies have evolved to account for repetitive genomic regions, they still struggle to assemble highly heterozygous parts of diploid genomes (Rice et al. 2020). This was reflected in the fact that some immune loci were assigned to scaffolds that were too small for their rate of recombination to be inferred with confidence. This was an issue for genes within the immunoglobulin kappa locus and genes located within the immunoglobulin heavy-chain locus (Table S4). Our results highlight the need for complete telomere-to-telomere haploid genomic sequences, not only for humans, apes, and agricultural and companion animals (Rice et al. 2020; Nurk et al. 2022; Bredemeyer et al. 2023), but also for species of conservation concern.

### Population declines coincide with glacial environmental shifts

Our results provide a compelling illustration of the impact of the mutation rate used to scale the timing of demographic inferences. Notably, our application of the koala mutation rate led to demographic plots that were drastically different from those scaled using the human mutation rate. Although our PSMC plots have the same shape as those previously inferred for koalas, we found that population declines occurred before the arrival of modern humans ∼65 to 47 kya (O’Connell and Allen 2015; Clarkson et al. 2017; Allen and O’Connell 2025), whereas previous studies suggested that these declines followed human arrival (Johnson et al. 2018; De Cahsan et al. 2025; Ahrens et al. 2026). There has been much debate over when humans first reached the continent (O’Connell et al. 2018; Bradshaw et al. 2023), but the arrival of humans into southern Australia, and the majority of the koala's range, most likely occurred toward the more recent end of this timeframe. While both our PSMC and SMC++ results show that the koala population started to decline before human arrival, our PSMC estimates suggest sharp bottlenecks while our SMC++ estimates suggest a more gradual decline that partially overlaps with the presence of humans. In addition, compared with previous studies (Johnson et al. 2018; De Cahsan et al. 2025), our PSMC and SMC++ analyses both infer an earlier expansion of koala populations 700 to 200 kya, aligning with the oldest known fossil of the present-day koala 350 kya (Black et al. 2014; Figs. 3a and 4a).

We estimate that a bottleneck occurred in the ancestral koala population before it split into the present-day populations in the last 30 ky. These results are substantially different from previous reports of the populations diverging 250 to 200 kya (De Cahsan et al. 2025) or the ancestor diverging into three populations >300 kya (Lott et al. 2022). After the populations began to diverge, we estimate slight recoveries in the three northernmost populations but not in the two southern populations. This pattern is consistent with the present-day diversity in each of the populations, pointing to the influence of pre-European processes. However, it is well documented that southern populations experienced more severe recent bottlenecks due to a prolific fur trade around the turn of the 20th century (Phillips 1990; Menkhorst 2008), leading to decreased diversity and increased inbreeding (McLennan et al. 2025). The impact of these recent processes on SMC++ analyses is not well understood, and they could potentially be driving the observed patterns in historical sizes of the southern populations. This bias could also explain the reduced resolution (number of time intervals) in the analysis of the Victorian population, for which a slightly later bottleneck was inferred than for the other populations. Given that the Victorian population was not isolated until after the bottleneck, the inferred later bottleneck is unlikely to be biologically meaningful.

Population declines during the last 110 ky coincide with the onset of the most recent glacial period. During this period, koalas became increasingly restricted to habitat refugia along the eastern and southwest coasts (Adams-Hosking et al. 2011). The emergence of the Nullarbor (meaning “no tree’) Plain, the treeless southern central region of Australia, is likely to have driven the final separation of the eastern koala populations from the now-extinct western population (although a few koala populations have been re-established in Western Australia using translocated individuals from the eastern population). During the mid-Pleistocene, the Nullarbor Plain harbored a diverse collection of herbivores that included large-bodied arboreal species such as koalas and tree kangaroos, suggesting a higher density of trees compared with the present (Prideaux et al. 2007). This would have provided a connection between eastern and western eucalypt forests before the landscape transitioned to semi-arid shrubland by ∼70 kya to form a vast biogeographical barrier between the western and eastern koala populations (van der Kaars et al. 2017; De Deckker et al. 2021; Adeleye et al. 2023). The appearance of this barrier would have led to a substantial reduction in the effective population size of koalas, matching the declines observed in our demographic models. Pre-human population declines in koalas support previous findings of progressive changes to mammalian assemblages before the arrival of humans (Price and Webb 2006; Price et al. 2012) and the idea that changes to the environment, associated with increasingly arid conditions, had substantial impacts on the Australian fauna during the last glacial period.

Koalas would have become increasingly restricted to the east and west coasts as they approached the Last Glacial Maximum (Adams-Hosking et al. 2011). This is consistent with the declines inferred from SMC++, which suggest a continued decline until ∼20 kya. Humans first arrived on the Australian continent after the koala population had already started to decline, but humans have been credited with the extinction of the last remaining Western Australian population (Balme et al. 1978; Prideaux et al. 2010). Population expansions and divergences after the Last Glacial Maximum also coincide with the expansion of habitat across the east coast of Australia to the koala's large present-day range (Adams-Hosking et al. 2011). However, the environmental conditions of the current interglacial period have remained too arid to allow the re-expansion of koalas across South Australia into suitable habitat in Western Australia.

Historical population sizes estimated using the sequentially Markovian coalescent (e.g. PSMC and SMC++) rely on the assumption that a population's inverse instantaneous coalescent rate is directly correlated with its effective population size. However, a reduction in the inverse instantaneous coalescent rate can be caused not only by a reduction in population size, but also by increasing structure with reduced migration and gene flow (Mazet et al. 2016; Chikhi et al. 2018; Mather et al. 2020). Therefore, it is possible that the inferences from these methods indicate a decline in connectivity between koala populations during this period instead of reductions in size. However, decreased population size and increased population structure would both support the notion that koala habitat became more restricted during the last glacial period. It is likely that these two processes occurred in tandem.

Another important source of uncertainty in demographic inference is generation time, which, together with the mutation rate, is used to scale the plot of effective population size. In koalas, generation time has been estimated at 6 to 8 years (Phillips 2000) and, in accordance with previous demographic studies, we used the midpoint (7 years) to scale our results. If the true generation time is shorter, inferred population declines would shift toward the present. However, a decline estimated to have begun ∼110 kya under a 7-year generation time would shift to ∼94 kya under a 6-year generation time, which still predates human arrival in Australia. Thus, while generation time remains a substantial source of uncertainty in demographic analyses of non-model species, modest variation does not alter our primary conclusions about the timing of population-size changes.

### Inference of recent population histories is highly dependent on recombination rate

Our results also provide a compelling illustration of the impact of the recombination rate used to infer recent demographic histories. Notably, our application of population-specific koala recombination rates led to demographic plots that were drastically different from those estimated using the default recombination rate. The impact of recombination rate on recent demographic analyses is more complicated than the impact of the mutation rate on SMC analyses. Varying the recombination rate scales the genetic map that GoNe2 uses to generate bins of recombination rates across loci, and alters demographic patterns beyond simply their size and timing. Using the default recombination rate of 1 cM/Mb, we inferred stable population sizes in NSW and QLD over the last 100 generations, with sudden declines only commencing in the last 25 generations. This more closely aligns with the original analysis of this data set using the default recombination rate (Ahrens et al. 2026). Using a reduced recombination rate of 0.5 cM/Mb also produced similar demographic patterns. Instead, applying the koala recombination rates (0.074 to 0.140 cM/Mb) uncovers more complex demographic histories, especially in NSW and QLD, which show earlier population declines, similar to those seen in VIC populations. Overestimating the recombination distance between SNPs, by using a recombination rate that is too high, is expected to push demographic events toward the present (Tenesa et al. 2007). This pattern occurred in some populations, but the impact was often more complex and, in some cases, entire demographic patterns (e.g. bottlenecks) were inferred using the population-specific recombination rates but not when using the default recombination rate (Figure S6). These results demonstrate the importance of evolutionary parameters in demographic models, and how they can have a meaningful impact on the interpretation of results. We recommend that conservation management decisions based on demographic analyses such as GoNe2 should be done so with caution and the sensitivities to unknown parameters in non-model organisms should be clearly communicated, especially for threatened species.

We inferred population declines across all three eastern Australian states over the last 75 generations. If we assume a generation time of 7 years for koalas (Phillips 2000), the European arrival to Australia did not occur until ∼35 generations ago, after koala populations were estimated to decline. Our results suggest that koala populations had already declined before the arrival of Europeans, which is consistent with evidence suggesting that populations were relatively small at the time of first European settlement (Black et al. 2014). However, population declines associated with a booming fur trade in the 18th and 19th centuries are well documented across all three states, particularly in Victoria, and are not well reflected in our results or those inferred previously (De Cahsan et al. 2025; Ahrens et al. 2026). Declining effective population size over the last 10 generations in QLD and NSW populations does align with population census estimates in these states (Adams-Hosking et al. 2016). However, stable effective population sizes for Victorian populations during this period do not reflect the rapid increase in census population size in recent decades (Menkhorst 2008).

Another possible explanation is that the generation time for koalas has been overestimated, but this would require the generation length to be 50% shorter for population declines to align with the arrival of Europeans and even lower to align with the fur trade. GoNe2 is also limited in its ability to accurately estimate the timing of demographic events and can infer declines to start before and end after the true timing of simulated sharp population bottlenecks (Santiago et al. 2025). Overall, these results highlight that the recombination rate is a critical parameter in demographic inference based on linkage disequilibrium, with inaccurate estimates capable of substantially altering inferred demographic trajectories and their interpretation.

## Conclusions

Genome-scale analyses of demographic and evolutionary history are allowing increasingly fine-grained inferences of complex historical processes. We have shown, however, that our ability to do so in non-model organisms, which often contain the most fascinating histories, is hindered by gaps in our knowledge of fundamental evolutionary parameters. With a growing catalogue of mutation rate and recombination rate estimates for organisms across the Tree of Life, our demographic modeling will become increasingly accurate and will allow us to resolve the drivers of historical population-size dynamics.

In the koala, our use of a direct estimate of the mutation rate led to a shift in the estimated timing of population-size changes, with important consequences for identifying the drivers of population declines. In particular, we found that late Pleistocene population contractions occurred before the arrival of humans on the continent. This shifts the narrative away from anthropogenic declines of koalas and highlights the substantial ecosystem changes that have occurred during the history of the continent. Broader analyses of the Australian fauna are required to determine whether this pattern is unique to koalas or if environmental change before human arrival has driven similar declines in other species.

We have also demonstrated that using species-specific mutation rates allows more accurate estimation of recombination maps. Using the average recombination rates from these maps, we modeled recent population-size changes in koalas and demonstrated the importance of species-specific recombination rates in the absence of a known genetic map. By applying our recombination rate estimates, we inferred comparable population declines across koala populations in all three states, with the largest recovery in Victorian koalas. These results provide a useful context to help conservation managers tailor management strategies to a population's needs.

## Materials and methods

### Whole-genome resequencing of parent-offspring trios

Our data set comprised 12 resequenced genomes from five male and seven female koalas, from five putative parent-offspring trios from three families. Eight individuals forming three trios were sourced from skins at the Australian Museum from a captive-bred colony at Featherdale Sydney Wildlife Park. Four live individuals forming two trios (two full siblings) were selected from the Taronga Conservation Society Australia's breeding program, with DNA extracted from blood. All genome sequences were produced in the first stage of the Koala Genome Survey (Hogg et al. 2023; McLennan et al. 2025). We downloaded BAM files from the Amazon Web Services Open Data Sponsorship program (Australasian Genomes; https://koalagenomes.s3.ap-southeast-2.amazonaws.com/index.html). Details of DNA extraction, sequencing, and read mapping were reported previously (Hogg et al. 2023; McLennan et al. 2025). These bam files were produced using the koala reference genome (GCA_002099425.1_phaCin_unsw_v4.1; Johnson et al. 2018), which comprises 1906 scaffolds with an N50 of 11.6 Mb. The average genome coverage across koala samples forming trios was 32×. De novo mutations, false-positive rates, and false-negative rates were calculated using the methods described by Bergeron et al. (2022), with the additional false-positive steps introduced by Bergeron et al. (2023).

The Koala Genome Survey variant call format (VCF) file was updated to include all available koala genomes (n = 474), including the samples from a recent population genetic analysis (n = 413) (McLennan et al. 2025), captive-bred individuals from our parent-offspring trios and Dubbo (n = 12 and 5 respectively), as well as wild representatives from South Australian populations on Kangaroo Island (n = 20) (Gates et al. 2025) and the Mount Lofty Ranges (n = 24). We used individual genomic variant call format (GVCF) files for each sample as input to Illumina's DRAGEN Germline Pipeline (v4.3.6; Illumina), using the koala reference genome (GCA_002099425.1_phaCin_unsw_v4.1; Johnson et al. 2018) to perform joint genotyping across all samples. This uses the DRAGEN gVCF PopGen Genotyper (4.3.6; Illumina) to produce a single, multi-sample variant calling file (VCF) used for all downstream analyses. Depth (DP) was calculated for all sites in all samples using bcftools (v1.17) by summing the localized allelic depths (LAD). Our unfiltered VCF contained 59,694,393 SNPs and 474 individuals. Variants were filtered using bcftools to remove sites that did not pass Illumina's QUAL filter and had read depth <15 or >60, leaving 47,655,085 SNPs. Finally, we filtered out variants that were missing in more than one individual, leaving 17,573,439 SNPs. Custom scripts based on those developed in Bergeron et al. (2022) and Bergeron et al. (2023) for the estimation of mutation rates are available at https://github.com/tobykovacs796/koalamutrate.

### Recombination maps

A subset of unrelated individuals from each of the five populations was selected to infer population-specific recombination landscapes. Relatedness (kinship) between individuals was estimated within each of the five populations identified in McLennan et al. (2025) using vcftools *–relatedness2*, which implements the KING method (Manichaikul et al. 2010). We wrote a script to randomly select individuals and iteratively build a group of unrelated individuals containing five females and five males. The algorithm began by randomly choosing one individual, then repeatedly searching the population for an unrelated individual, adding each as it was found, until either a group of ten was assembled or no additional unrelated individuals could be added. When multiple valid sets of ten were possible, we prioritized individuals to maximize genome coverage and ancestry representation. Variants for these individuals were subsetted from the unfiltered multi-sample VCF. ReLERNN requires that SNPs residing in transposable elements be masked before the analysis. As such, we identified the genome-wide transposable element content of the reference genome using RepeatMasker (version Open 3.2.6 A.F.A. Smit, R. Hubley & P. Green RepeatMasker at http://repeatmasker.org). This program was run using the -qq parameter and using the Zoonomia repeat library (Osmanski et al. 2023). To prepare variants for generating a recombination map, we removed variants overlapping a transposable element annotation in the reference genome. We further filtered variants, removing variants within 5 bp of an indel and those that did not meet the following quality criteria:e'%QUAL < 30 | INFO/DP < 16 | INFO/DP > 62 | QD < 2 | FS > 60 | SOR > 10 | ReadPosRankSum < −8 | MQRankSum < −12.5 | MQ < 40′ in bcftools (https://github.com/samtools/bcftools). Using VCFtools (https://vcftools.github.io/man_latest.html), we removed indels and subset the data to include only biallelic SNPs for each clade for further analysis. Custom scripts used to contextualize and average the raw ReLERNN output are available at https://github.com/eutherialab/Foley_XLRD.

To model the genome-wide recombination rate for each koala population, we used ReLERNN (https://github.com/kr-colab/ReLERNN), a deep learning approach that uses recurrent neural networks (Adrion et al. 2020). Our estimate of the mutation rate was incorporated into the analysis. ReLERNN was run using the simulate, train, predict, and bscorrect modules with default settings. Given that we were interested in broadly evaluating the differences in recombination rates among populations, we averaged rates in 2 Mb blocks with a 50 kb step. A non-parametric Mann-Whitney test was used to determine if there was a significant difference between the populations with the highest and lowest inferred recombination rates.

We then investigated whether there was a difference between recombination rates inferred using an empirically estimated mutation rate for koalas compared with a “pan-mammal” yearly mutation rate of 2.2 × 10^−9^, estimated in a previous analysis of branch lengths (Kumar and Subramanian 2002). This was multiplied by 7 to produce a per-generation mutation rate for koalas to allow direct comparison with our empirically estimated mutation rate. We reran ReLERNN for a subset of populations using the pan-mammal mutation rate. Tests for normality were conducted using qq-norm in R. Spearman's rank correlation test was used to evaluate differences in the inferred recombination landscapes. A Mann-Whitney test was used to determine if there were differences in the mean of the rates between recombination maps.

### Phylogenetic network

The filtered VCF was read into a genlight object in R using *read.PLINK*. Euclidean distances were calculated using *bitwise.dist* in the package poppr, with the “scale_missing” setting. Using these genetic distances, we inferred a phylogenetic network using SplitsTree 6 (Huson and Bryant 2024). Individuals were then assigned to populations based on their maximum ancestry in previous *fast*STRUCTURE analyses (McLennan et al. 2025). Custom scripts for demographic analyses are available at https://github.com/tobykovacs796/koalamutrate.

### Demographic inference

Historical population sizes were estimated using a suite of methods that use the sequentially Markovian coalescent (SMC) as well as the site frequency spectrum. SMC methods are generally most accurate between 1,000 and 100,000 generations ago (Patton et al. 2019), with PSMC being more accurate for deeper timeframes in this range, and newer methods, such as SMC++, being more accurate for more recent timeframes due to the consideration of the site frequency spectrum (Terhorst et al. 2017). In order to demonstrate the impact of the mutation rate on demographic inference, we ran analyses using PSMC and SMC++.

We analyzed all 457 genomes from wild koalas using PSMC (Li and Durbin 2011). We trimmed the koala reference genome (GCA_002099425.1_phaCin_unsw_v4.1; Johnson et al. 2018) using vcftools (Danecek et al. 2011) to remove all contigs <50 kb and those putatively corresponding to the X chromosome (Hogg et al. 2024). We generated the input file for PSMC (Li and Durbin 2011) by constructing a diploid consensus sequence for each individual from their BAM file aligned to the trimmed reference genome, using samtools mpileup (v1.6) and bcftools (v1.3.1). Filtering followed the instructions given for PSMC (Li and Durbin 2011), including removal of duplicate reads, exclusion of sites with extreme sequencing depth (<1/3 or >2× the individual mean depth), downgrading mapping quality near indels, and masking low-quality bases (Phred < 20). PSMC was run using the time intervals “4 + 25 × 2 + 4 + 6”, assuming a generation length of 7 years (Phillips 2000), and with our estimate of the per-generation mutation rate. Individual demographic plots were grouped according to the previously assigned genetic clusters of koalas (McLennan et al. 2025). Captively bred koalas from Dubbo (n = 5), which were not included in previous analyses of population structure (McLennan et al. 2025), were placed across M NSW and S NSW based on our phylogenetic network. Because some of our initial PSMC plots displayed population spikes in single recent time intervals, we tried splitting the earlier time intervals as done by Hilgers et al. (2025) (2 + 2 + 25 × 2 + 4 + 6 and 1 + 1 + 1 + 1 + 25 × 2 + 4 + 6). This removed the spikes in some plots but added them to other plots where they did not originally occur. Hence, the final plot was selected to minimize these extreme, single-interval peaks, which are a known artifact of these methods.

Historical population sizes were also estimated for each of the five genetic populations using SMC++. Koalas were assigned to a population based on their highest ancestry as identified previously (McLennan et al. 2025). Koalas from Narrandera were not included in any population for SMC++ analyses because they are the result of recent translocations from SE QLD/N NSW and VIC populations (McLennan et al. 2025). Because the captively bread koalas from Dubbo are likely to contain recently mixed ancestry, they were excluded from SMC++ analyses. Koalas from South Australia, which were nested within the Victorian population in our phylogenetic network, originated from serial bottlenecking and inbreeding and so were not included in the analysis of Victorian koalas using SMC++.

For our demographic analyses using SMC++ (Terhorst et al. 2017), we used vcftools (Danecek et al. 2011) to filter the VCF to keep only biallelic sites and remove all contigs <50 kb and those that have been putatively identified as forming the X chromosome (Hogg et al. 2024). Historical population sizes were inferred in SMC++ using *estimate*, calculating the composite likelihood across several distinguished lineages. Distinguished lineages were selected based on the largest group of unrelated individuals that could be found in each population, with up to 10 distinguished lineages. We ran SMC++ with default settings with our estimated mutation rate, across the time interval 10^3^ to 10^6^ years. Initial analyses using the default number of knots (8) showed limited resolution over key timeframes of interest, so analyses were rerun with the number of knots set to 16, 32, and 50. These results showed increasing resolution with the number of knots (Figure S3) by introducing more inflection points and inferring different effective population sizes for a larger number of shorter time intervals. Several runs with 32 or 50 knots failed shortly after starting, with the error: *erroneous average coalescent time*. These runs were restarted until they completed successfully. The timing of population separation was inferred in SMC++ (Terhorst et al. 2017) using the function *split* and the marginal estimates for each population when using 50 knots. Custom scripts for demographic analyses are available at https://github.com/tobykovacs796/koalamutrate.

Demographic histories over the last 100 generations were modeled using GoNe2 (Santiago et al. 2025). We used the input .ped and .map files generated by Ahrens et al. (2026) for each of the 27 local government area (LGA) populations. This was done in order to minimize population structure within the populations, which can bias estimates of N_e_. Three LGA populations were removed from our analyses because they are known to contain individuals from two different genetic clusters (BLMT, NARR, and REDL) (McLennan et al. 2025). Due to applying lower recombination rates and GoNe2 requiring chromosomes to have a minimum length of 20 cM, we removed six scaffolds (3,6,7,8,9,10) from all populations using PLINK (v1.9.0; Chang et al. 2015). Three of the populations also contained missing SNPs and these sites were filtered out using PLINK (−geno 0.999).

We ran GoNe2 using the average recombination rate (-r) for the genetic cluster to which each LGA was previously allocated (McLennan et al. 2025). This parameter is used to convert a physical map (in bp) to a genetic map (in cM), if a genetic map is not available. Analyses were also run with the default recombination rate of 1 cM/Mb and 0.5 cM/Mb for comparison. Resulting N_e_ trajectories were plotted in ggplot2, and the geometric means were calculated for populations in each of the states QLD, NSW, and VIC to mirror the original analyses of Ahrens et al. (2026). Three populations were removed because they consisted of three or fewer individuals (FREI, GILB, and QLDM). One population (SGIP) with substantially higher estimates of N_e_ (average > 200,000) was not included in the estimates of the mean for that state (VIC).

## Supplementary Material

msag108_Supplementary_Data

## Data Availability

Raw whole genome re-sequences are available in The National Center for Biotechnology Information (NCBI) under BioProject PRJNA940526 at https://www.ncbi.nlm.nih.gov/bioproject/PRJNA940526. All outputs including the position of de novo mutations and relatedness values are available at https://github.com/tobykovacs796/koalamutrate and the Supplementary material.
