# Supplementary material for: Mutation rate estimate and population genomic analysis reveals decline of koalas prior to human arrival
Source: Mol Biol Evol. 2026 Jun 9;43(6):msag108. doi: 10.1093/molbev/msag108 (PMC13247528; doi:10.1093/molbev/msag108)
Supplement: msag108_Supplementary_Data [file msag108_supplementary_data.zip › Supplementary figure with captions.docx]

## Supplementary figures for “Mutation rate estimate and population genomic analysis reveals decline of koalas prior to human arrival”

Toby G. L. Kovacs^1^, Nicole M. Foley^2^, Luke W. Silver^1,3^, Elspeth A. McLennan^1^, William J. Murphy^2^, Carolyn J. Hogg^1,3^, Simon Y. W. Ho^1^

^1^School of Life and Environmental Sciences, University of Sydney, Sydney, Australia

^2^Veterinary Integrative Biosciences, Texas A&M University, College Station, TX, USA

^3^Australian Research Council Centre of Excellence for Innovations in Peptide and Protein Science, The University of Sydney, Sydney, NSW, Australia

Corresponding author: Toby G. L. Kovacs, toby.kovacs@sydney.edu.au

**Fig. S1** Historical effective population sizes estimated using PSMC for individuals across the five genetic populations. Backgrounds indicate the interglacial (warmer and wetter; pink) and glacial (cooler and drier; blue) periods of the last three glacial cycles. The dark grey column and human silhouette indicate the period in which humans are believed to have arrived and spread across Australia (65–47 kya; O’Connell & Allen 2015; Clarkson et al. 2017). PSMC plots are colored based on the individual’s average sequence coverage.

**Fig. S2** Historical effective population sizes estimated using PSMC for each koala individual using three different time interval settings. These include “4” when the first four intervals are grouped together (denoted as “4+…”), “22” when these intervals are subdivided into two groups (denoted as “2+2+…”) or “1111” when subdivided into four individual intervals (denoted as “1+1+1+1+…”).

**Fig. S3** Historical population sizes estimated using SMC++ for each of the five genetic groups of koalas using 8 (*a*), 16 (*b*), 32 (*c*) and 50 (*d*) knots. Pink and blue backgrounds indicate the interglacial (warmer and wetter) and glacial (cooler and drier) periods of the last three glacial cycles. The dark grey column indicates the period in which humans are believed to have arrived and spread across Australia.

**Fig. S4** Historical population sizes and population split times estimated using SMC++ for each pair of the five genetic groups of koalas using 50 knots. The black vertical line indicates the inferred split time between the two populations. Pink and blue backgrounds indicate the interglacial (warmer and wetter) and glacial (cooler and drier) periods of the last three glacial cycles. The dark grey column indicates the period in which humans are believed to have arrived and spread across Australia.

**Fig. S5** Variation in recombination rates across koala populations using the species-specific koala mutation rate and the pan-mammal mutation rate (PM). Boxplots showing genome-wide recombination rates. In each box, the × and the horizontal line represent the mean and the median recombination rate, respectively. Whiskers extend to the most extreme data points within 1.5 times the interquartile range from the first and third quartiles. Points beyond this are plotted.

**Fig. S6** Historical population sizes estimated using GoNe2 for each of 24 local government area populations of koalas analysed in Ahrens et al. (2026) grouped back states; Queensland (QLD), New South Wales (NSW) and Victoria (VIC). Each population was analysed under three recombination rate scenarios: 1 cM/Mb (red), 0.5 cM/Mb (green), and the mean recombination rate we estimated for the corresponding genetic cluster 0.074–0.140 cM/Mb (blue). Population codes for New South Wales: CAMT = Campbelltown, CLAV = Clarence Valley, KYOG = Kyogle, MONA = Monaro, NRIV = Northern Rivers, PILL = Pilliga, PMAC = Port Macquarie, PTS = Port Stephens; for Queensland: QLDM = Queensland Midcoast, SUNC = Sun Coast, TOOW = Toowoomba, MORB = Moreton Bay, GOLD = Gold Coast, GILB = Gilbert, FRAS = Frasier Coast, BURR = Burnett; for Victoria: COTW = Cape Otway, EGIP = East Gippsland, FREI = French Island, MALL = Mallacoota, MURR = Murray River, SGIP = South Gippsland, SHBS = Strathbogies, WVIC = Western Victoria.


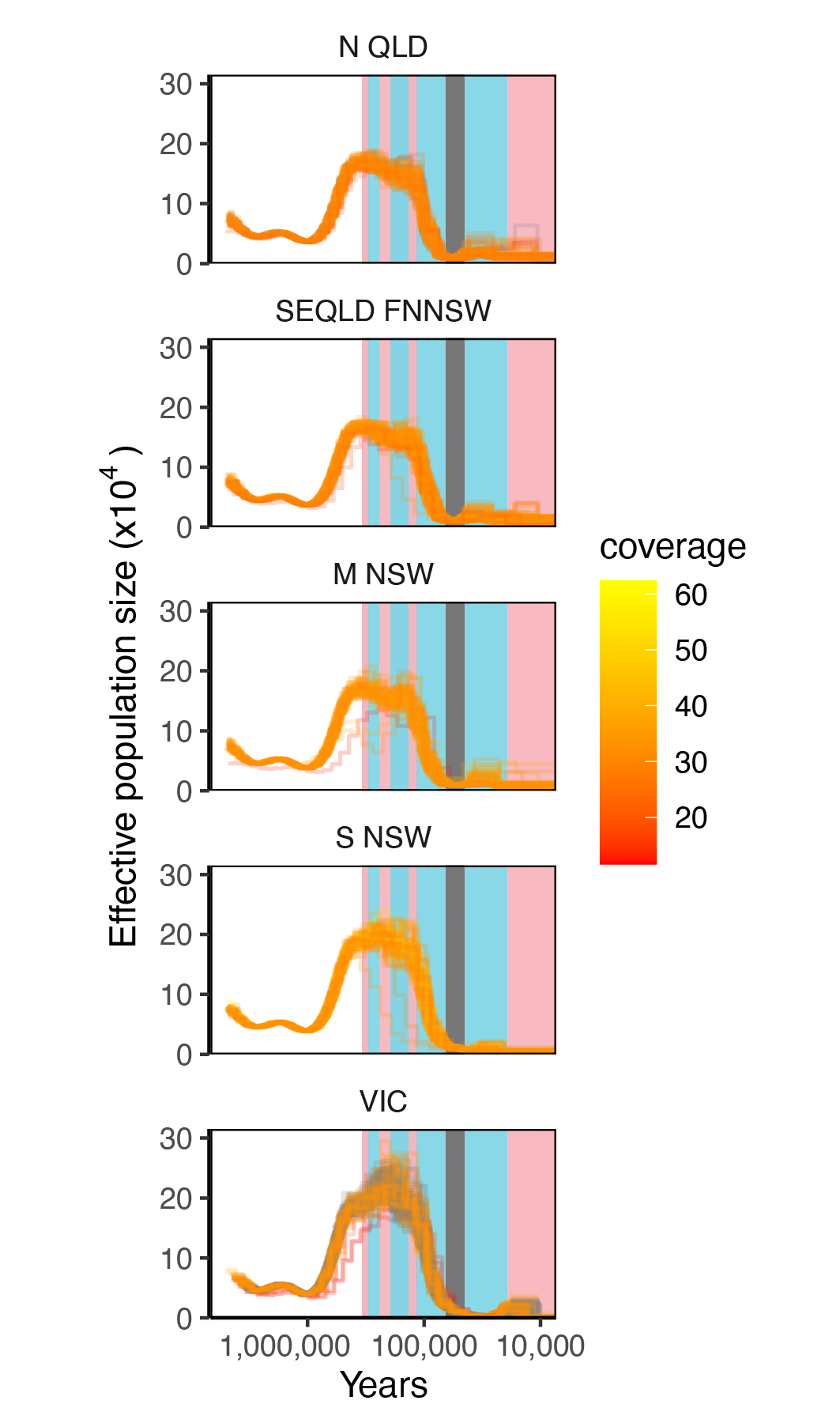


**Fig. S1**

**
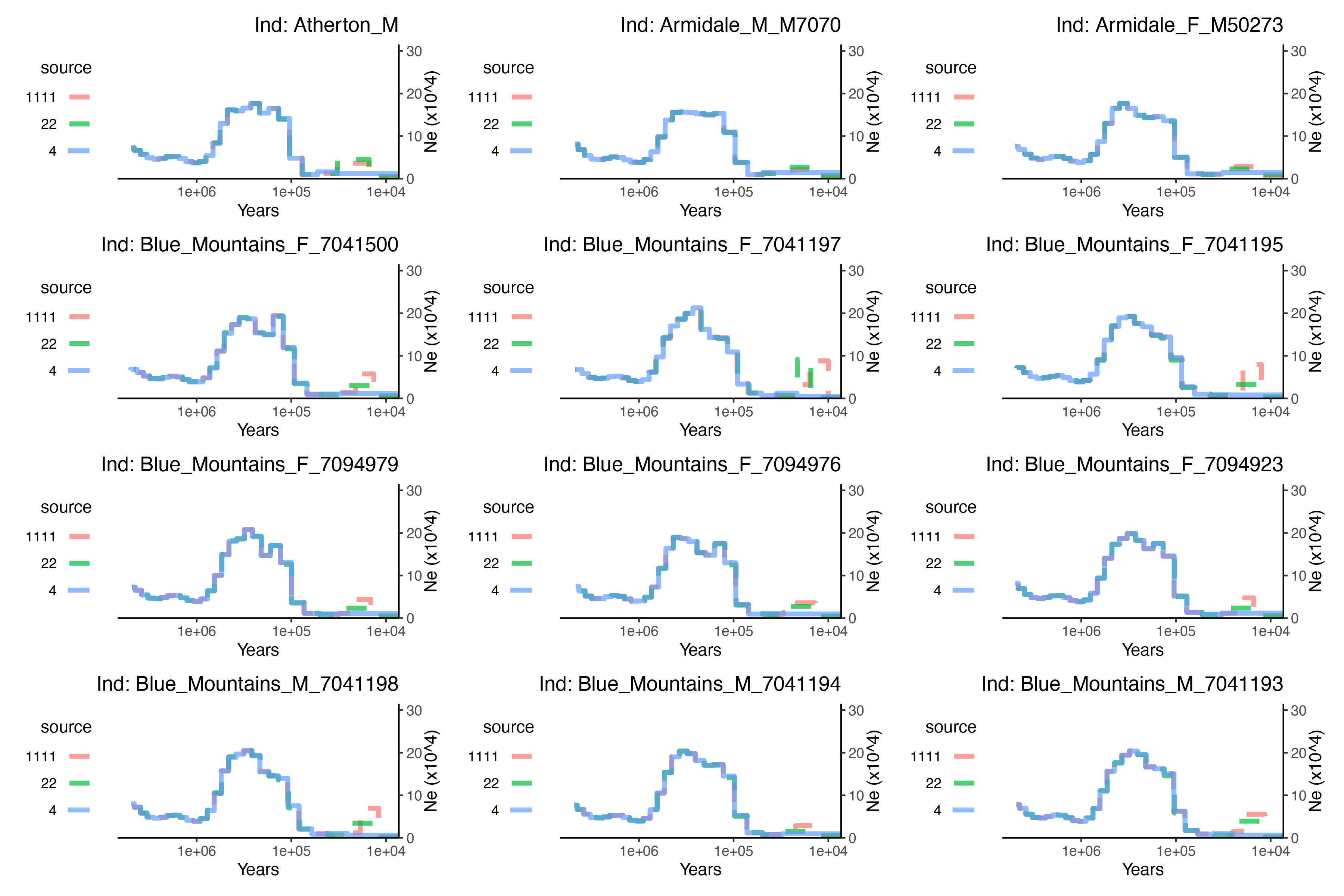

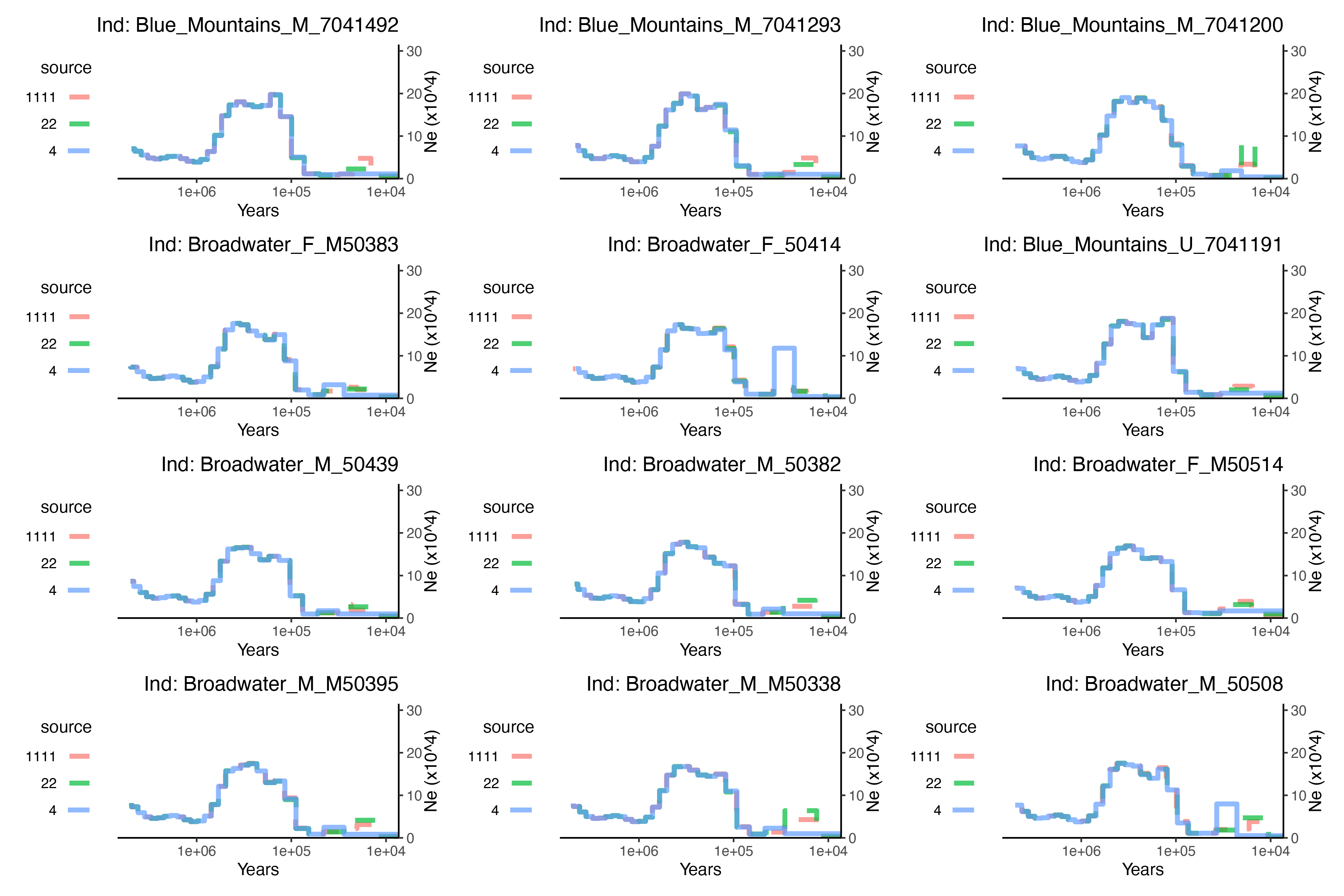
**

**Fig. S2**


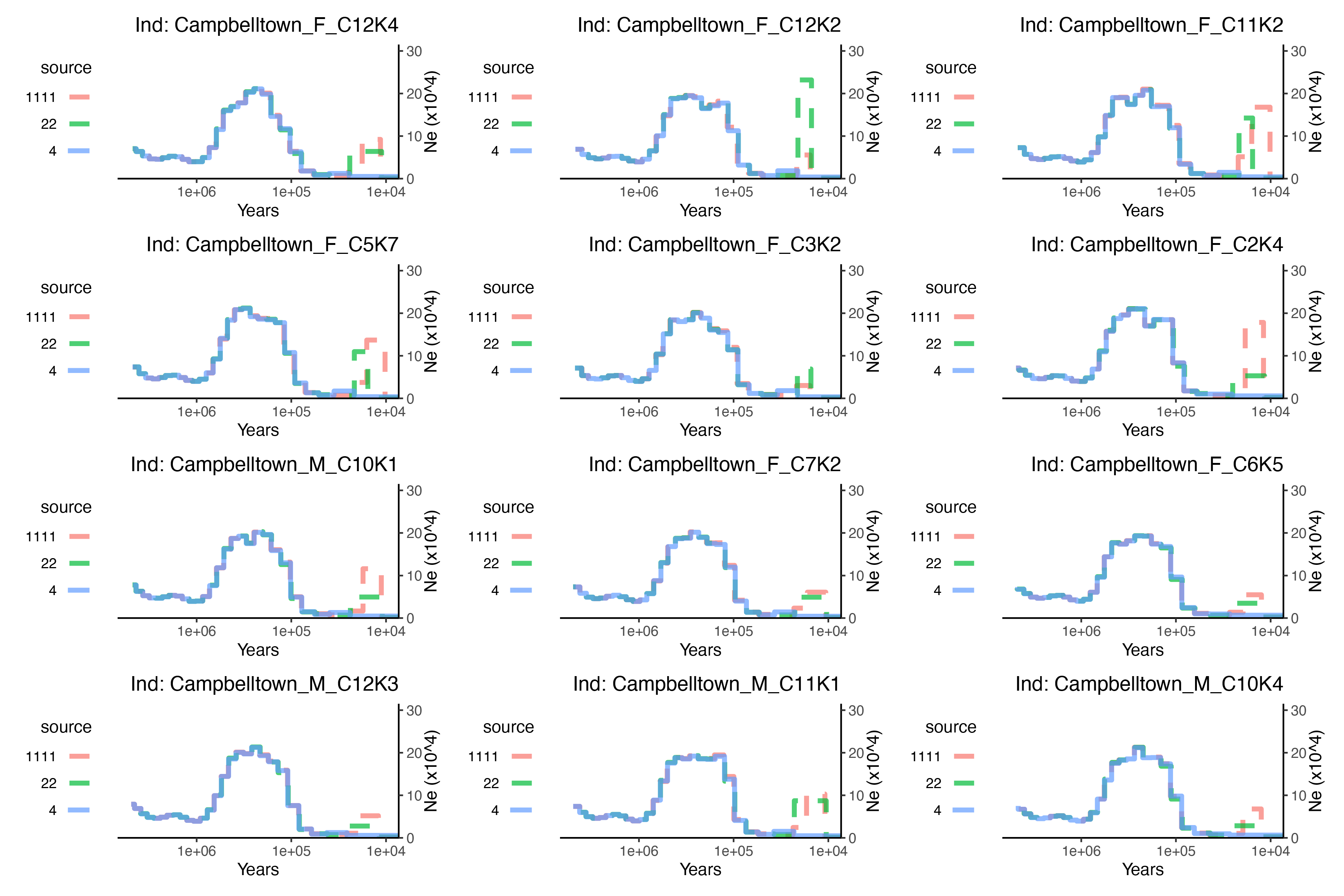


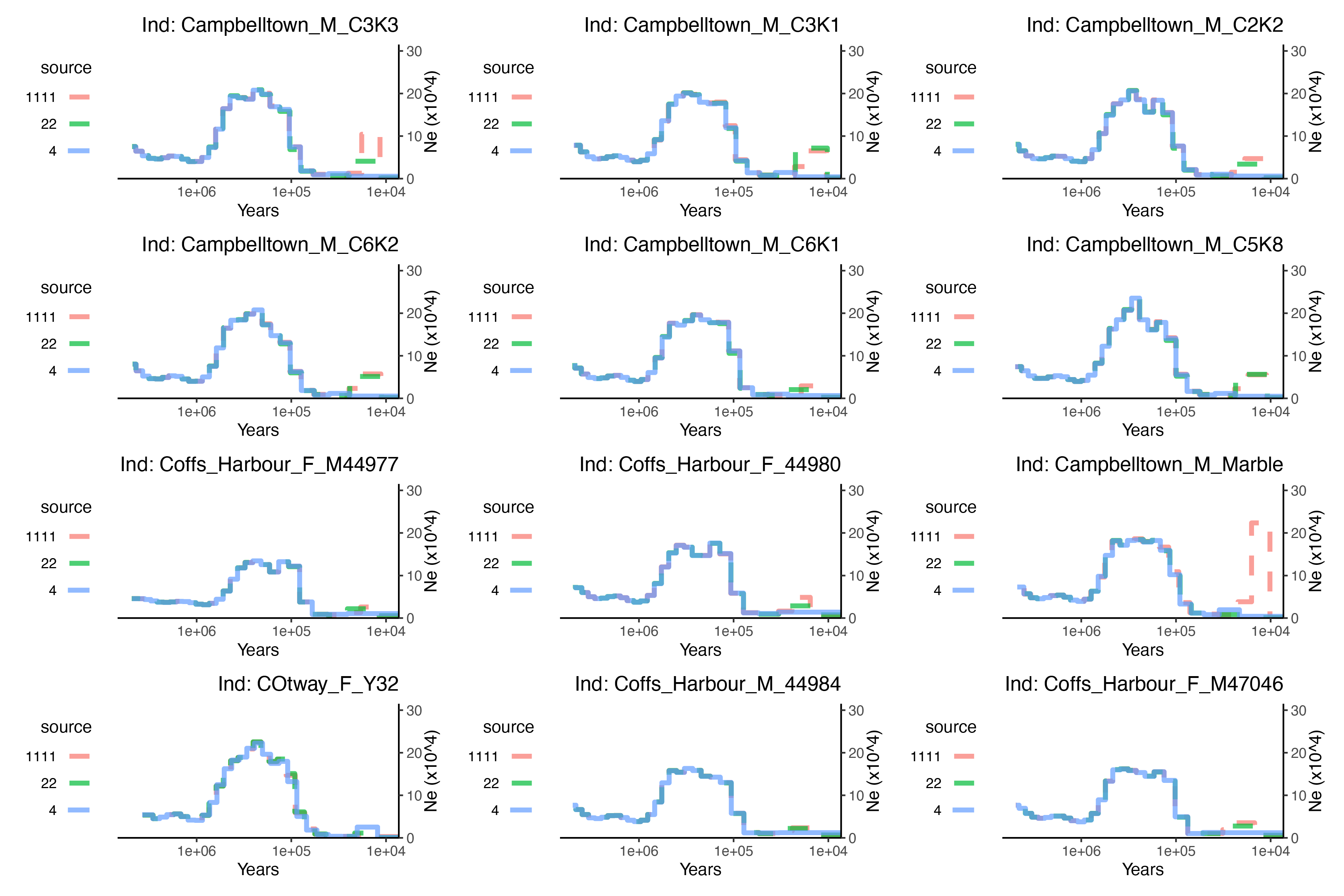


**Fig. S2 continued**

**
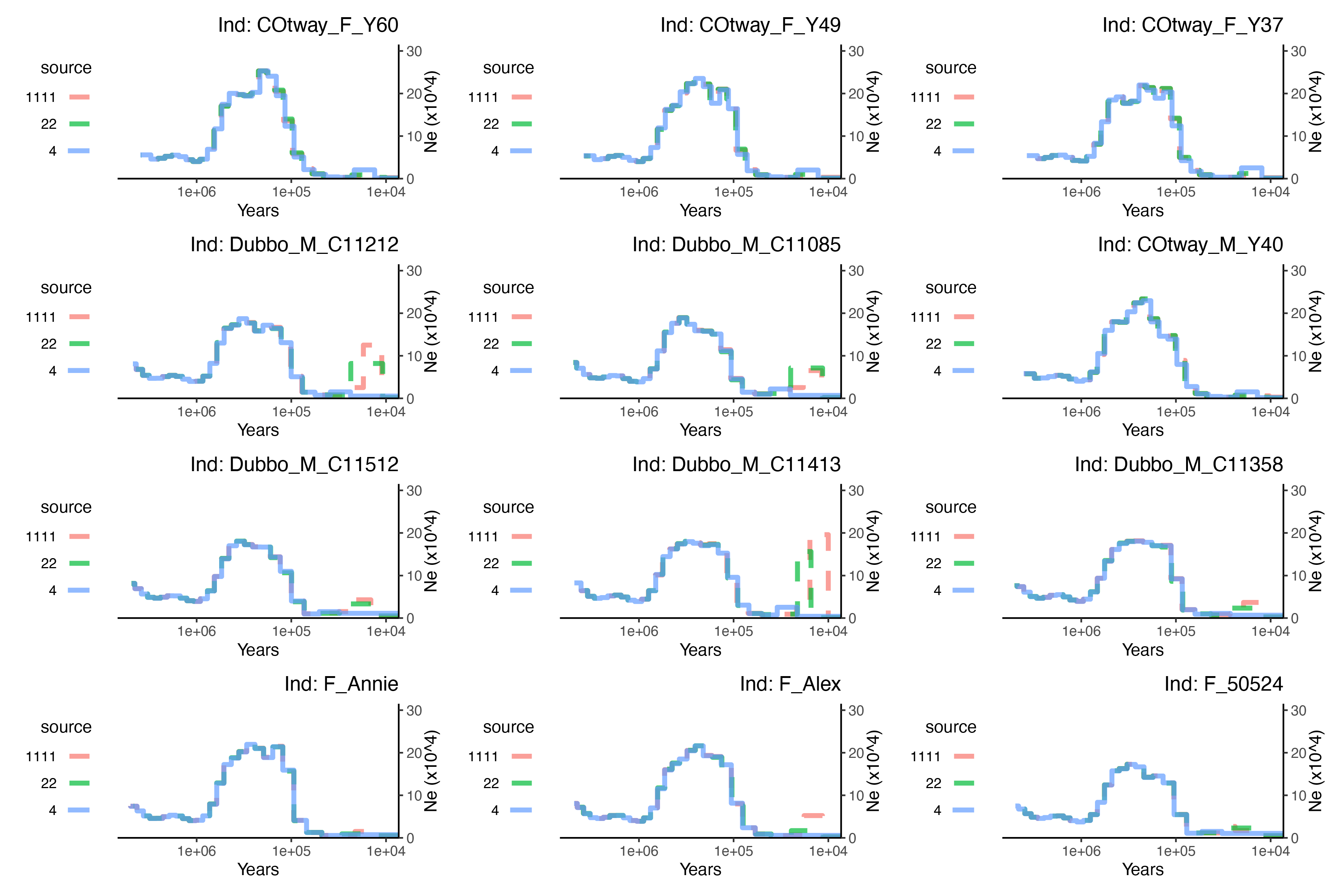

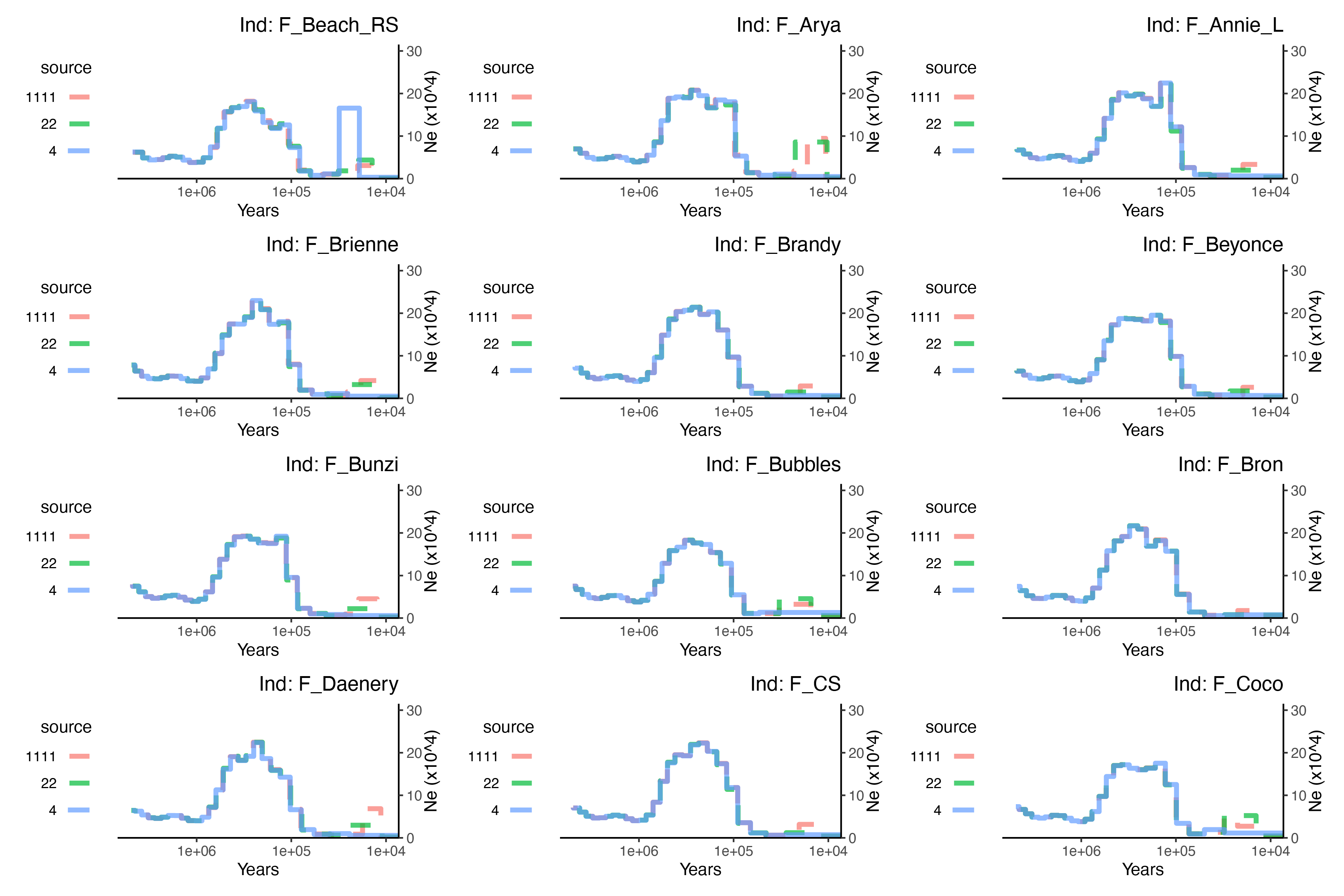
**

**Fig. S2 continued**

**
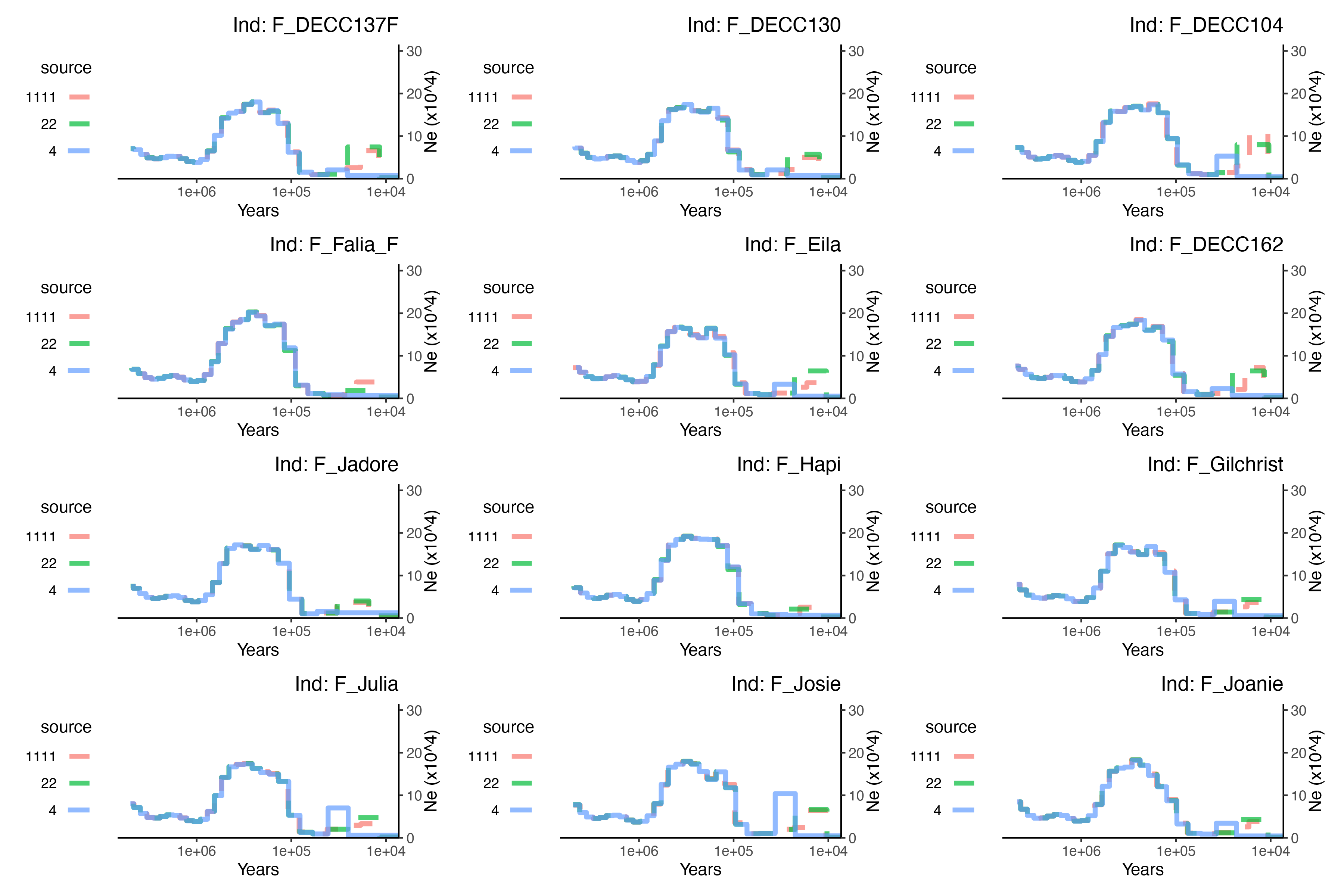

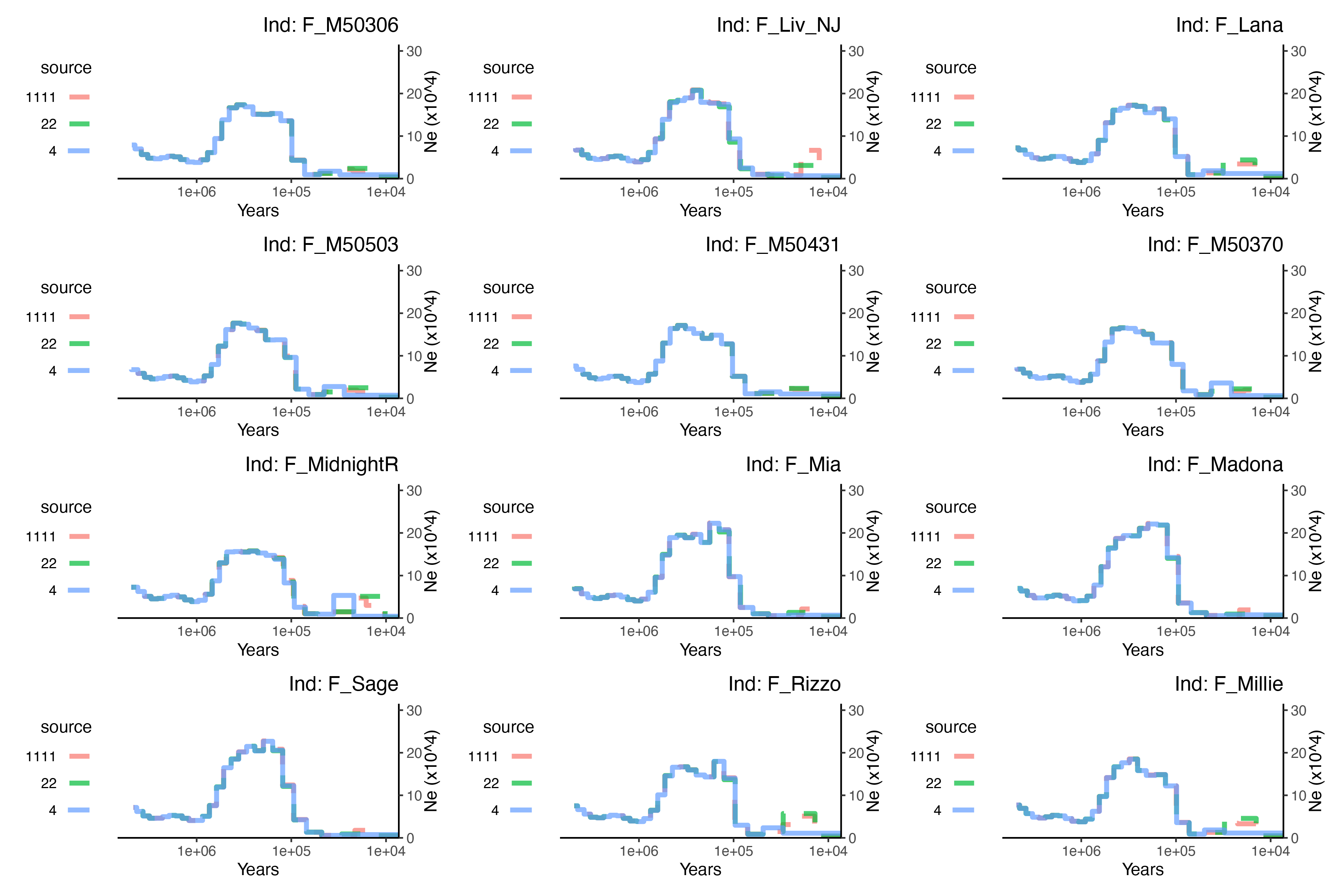
**

**Fig. S2 continued**

**
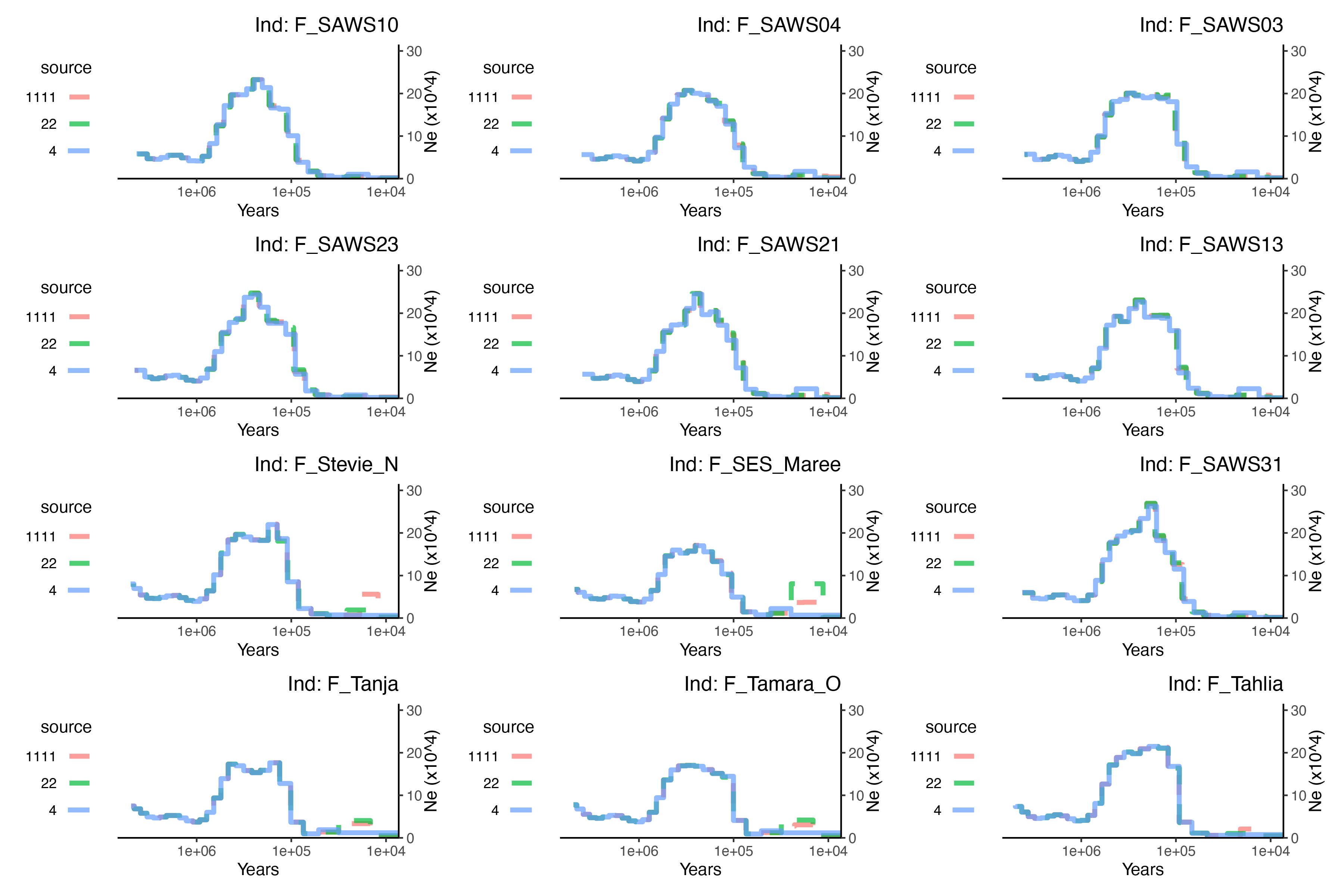

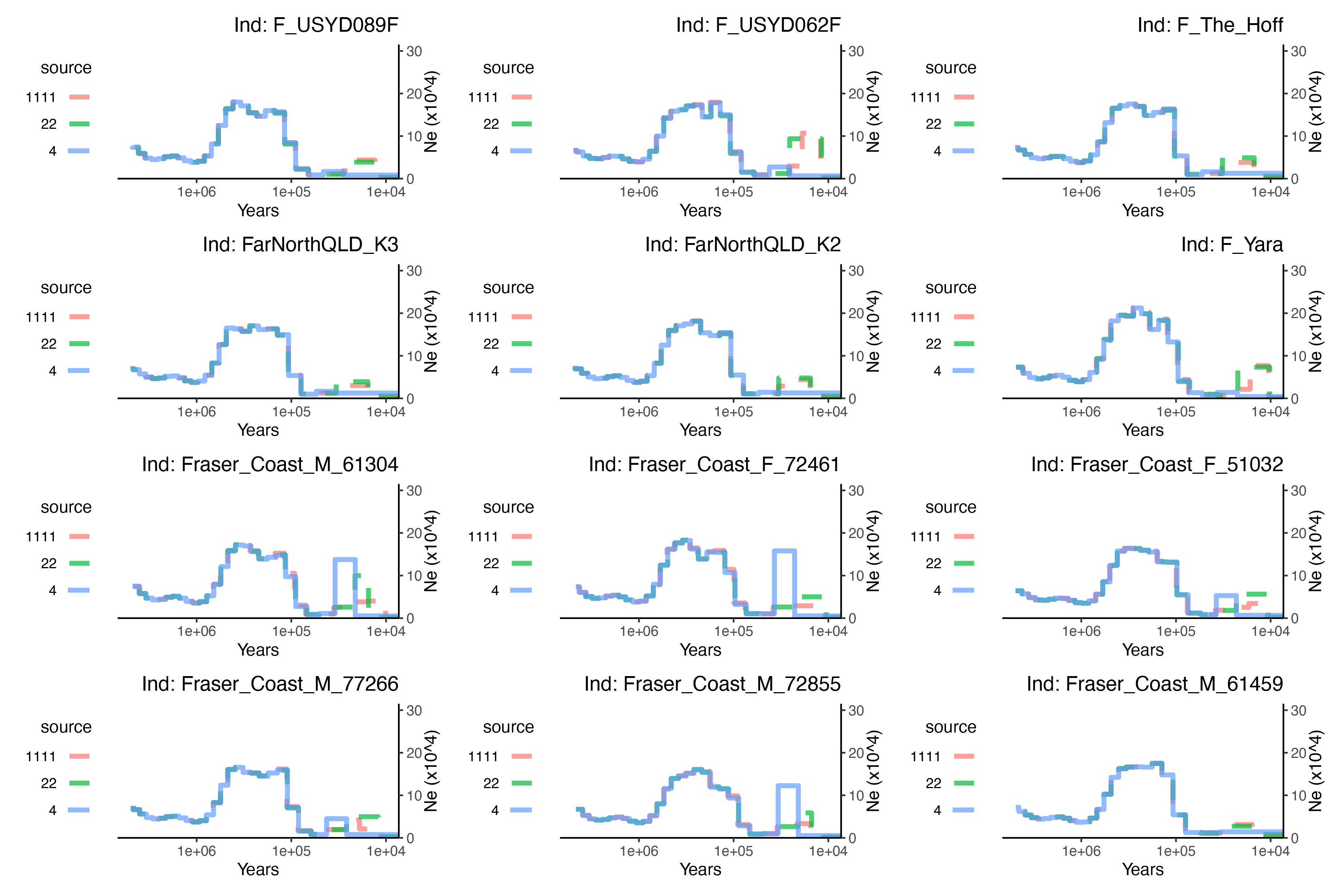
**

**Fig. S2 continued**

**
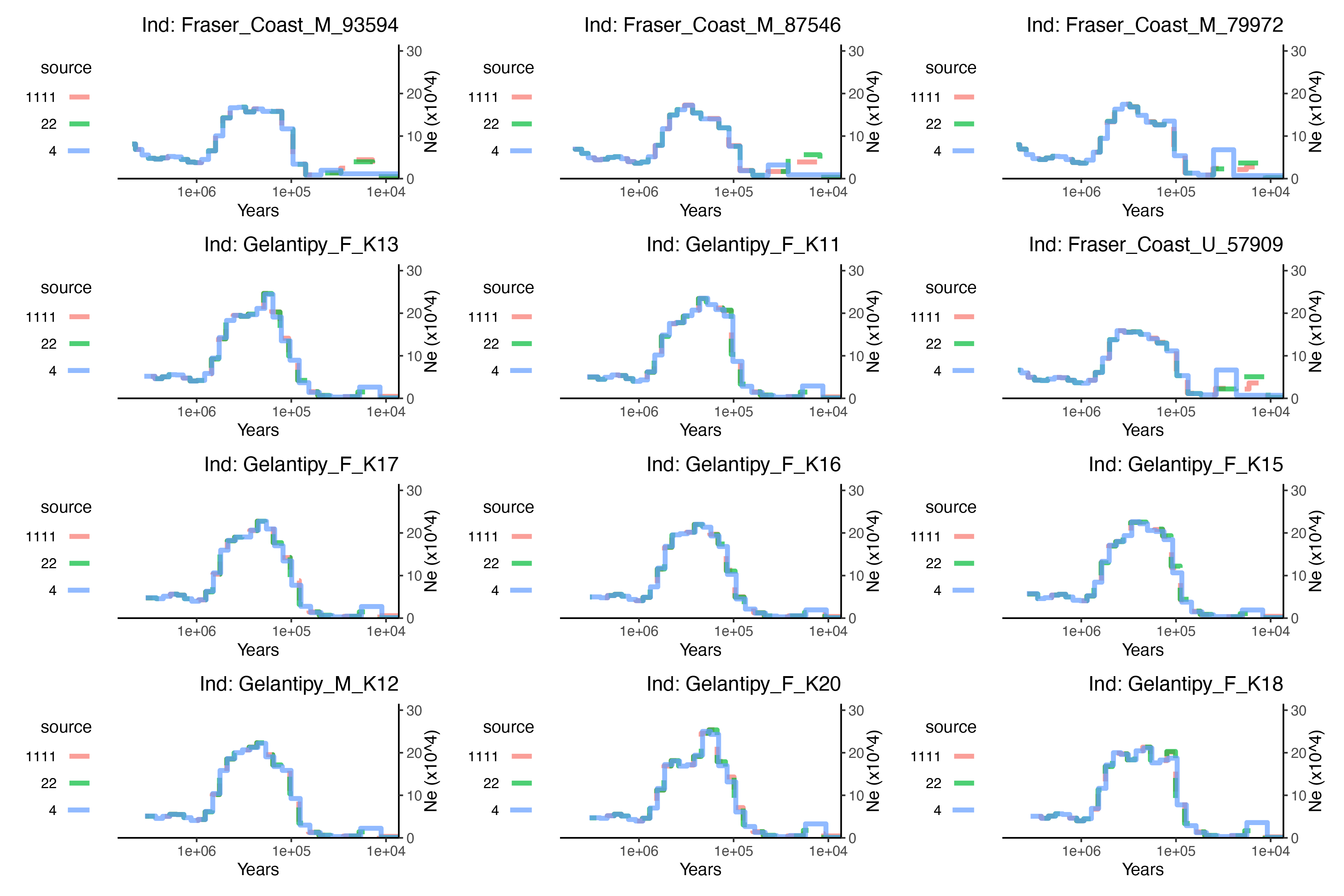

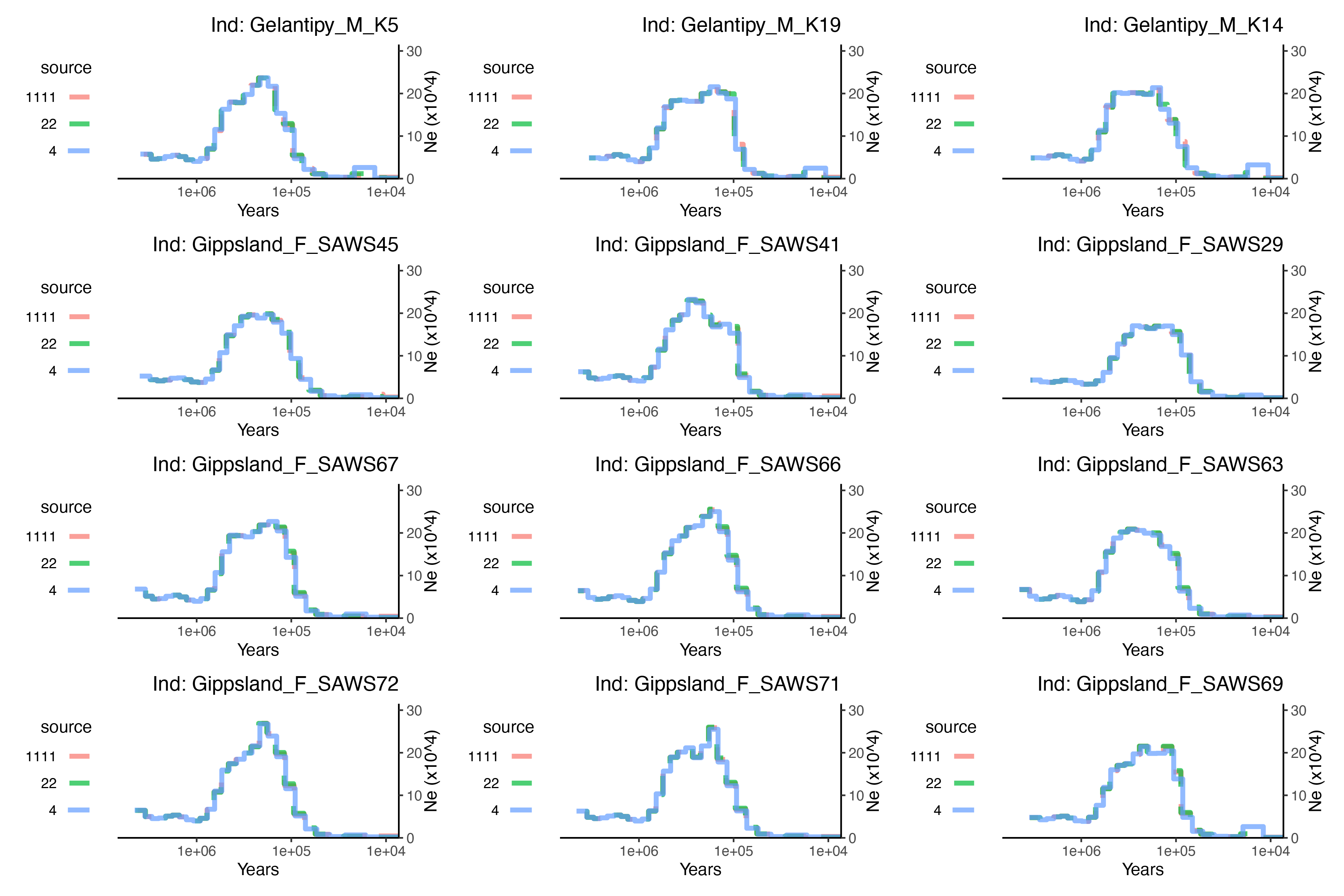
**

**Fig. S2 continued**

**
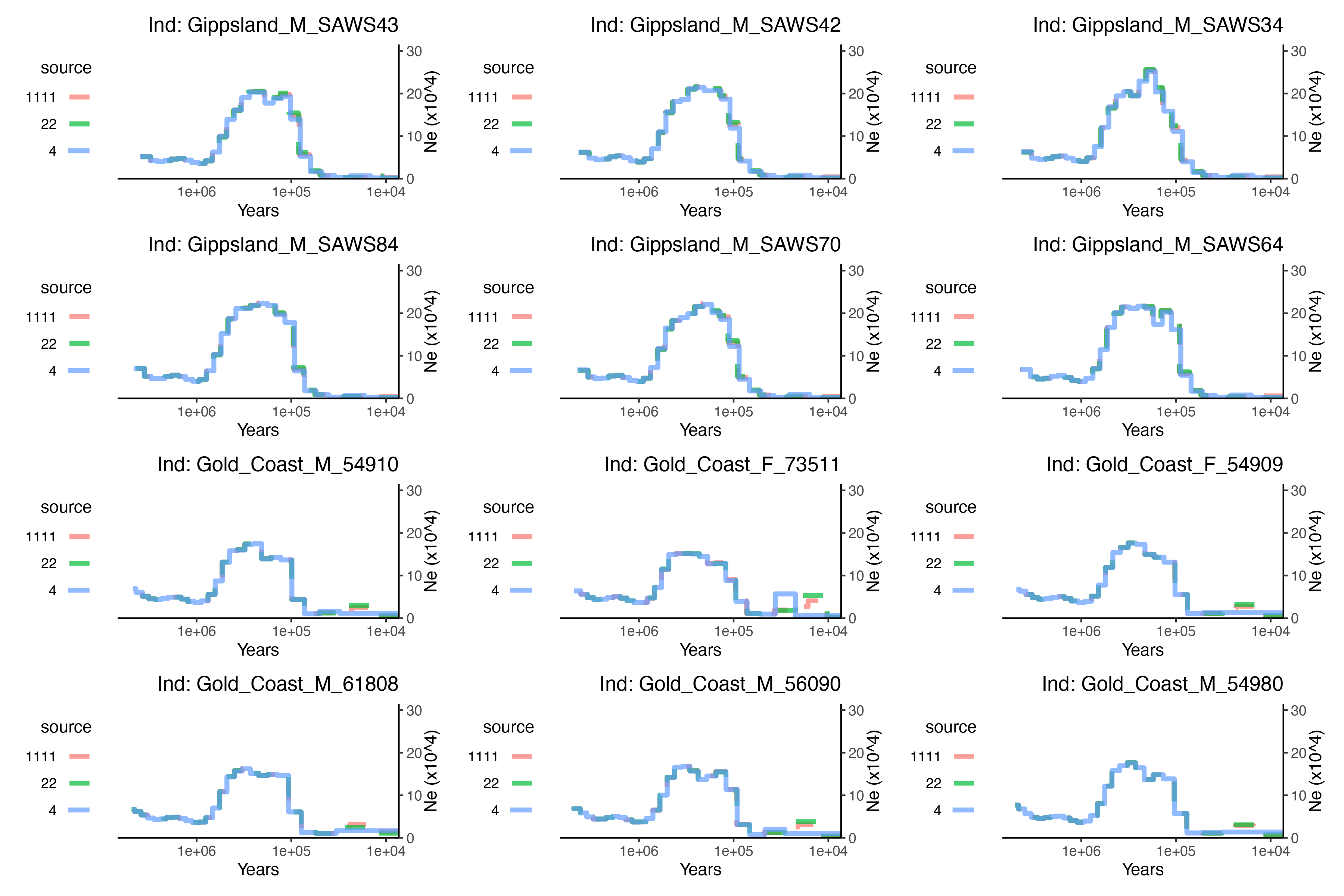

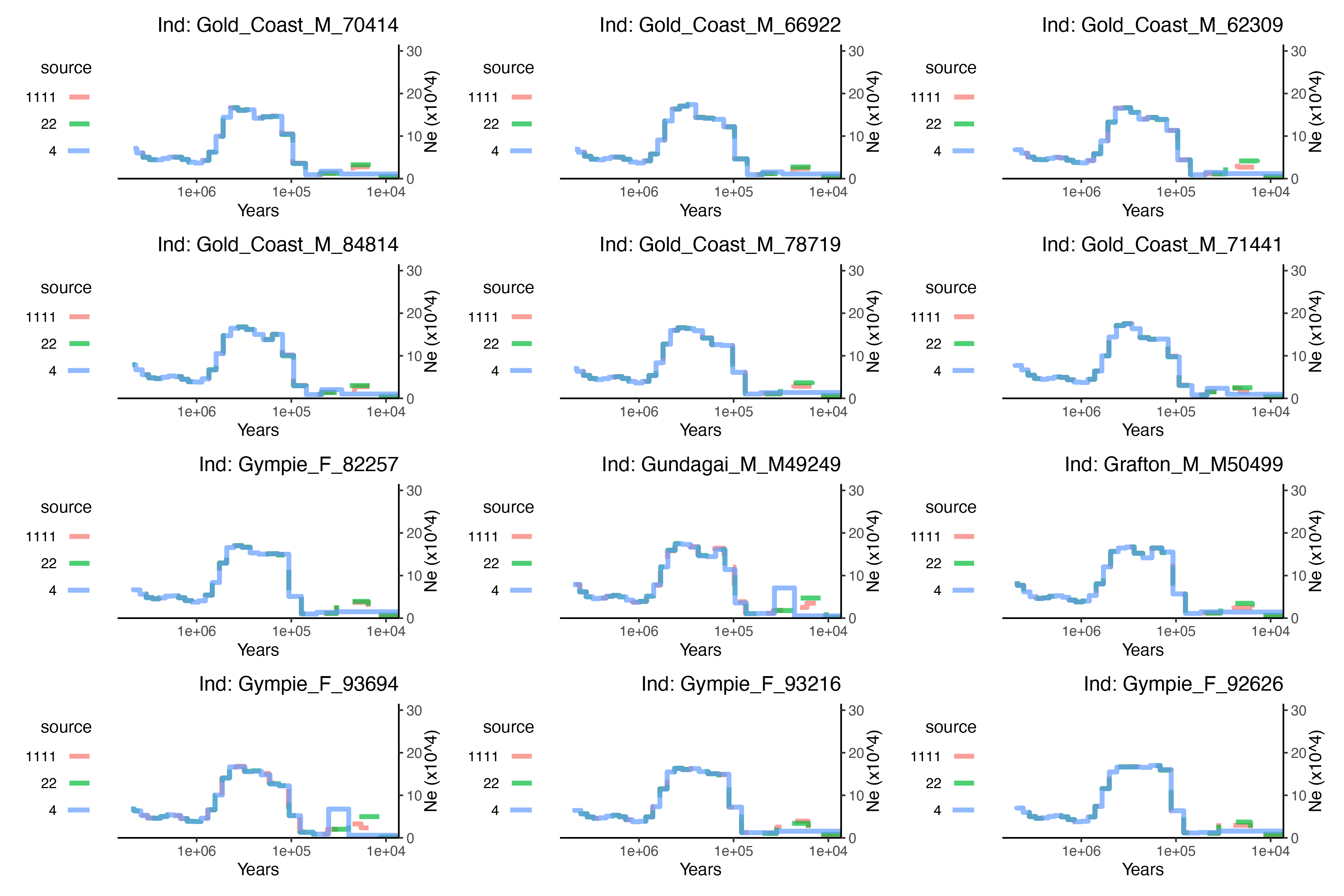
**

**Fig. S2 continued**

**
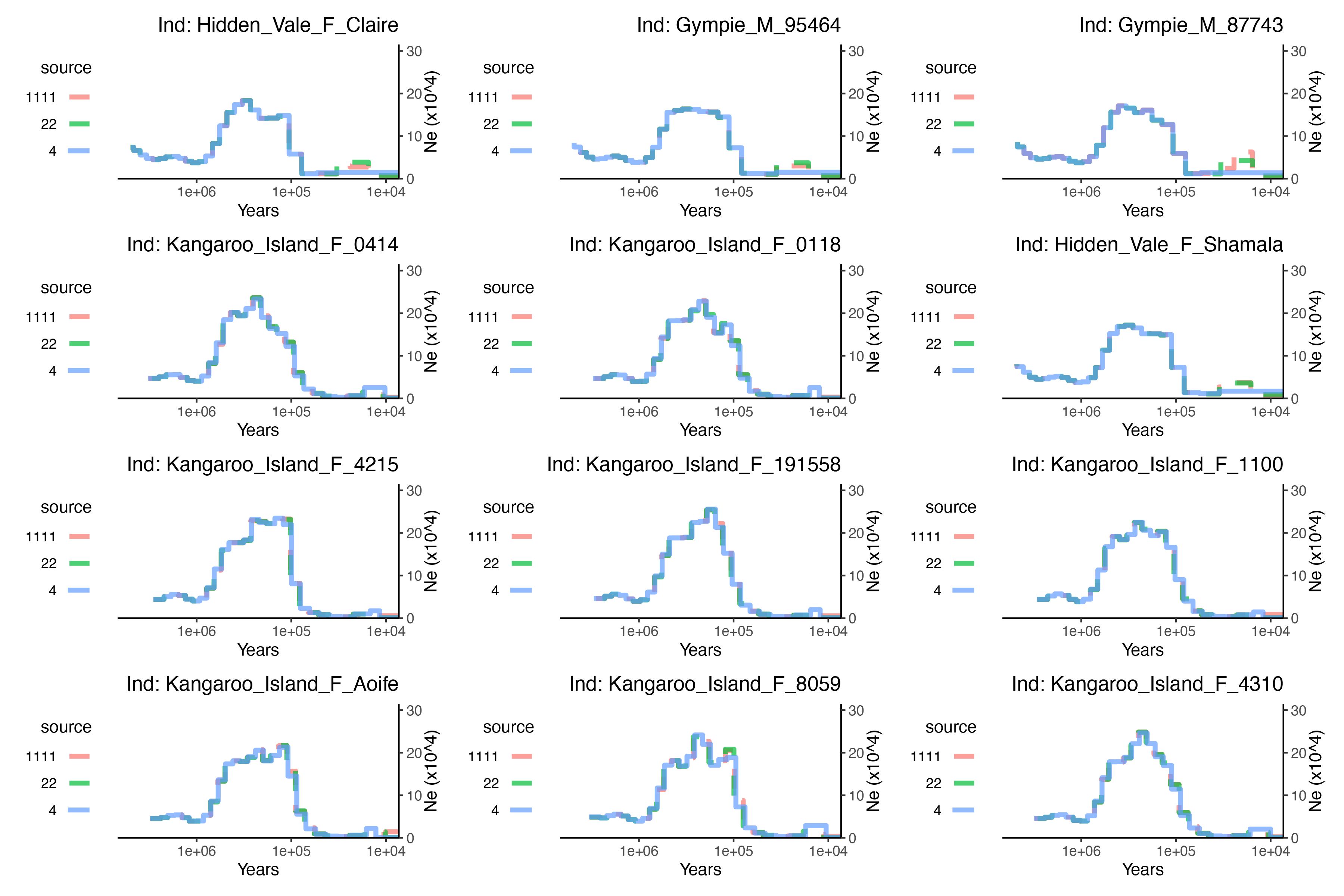

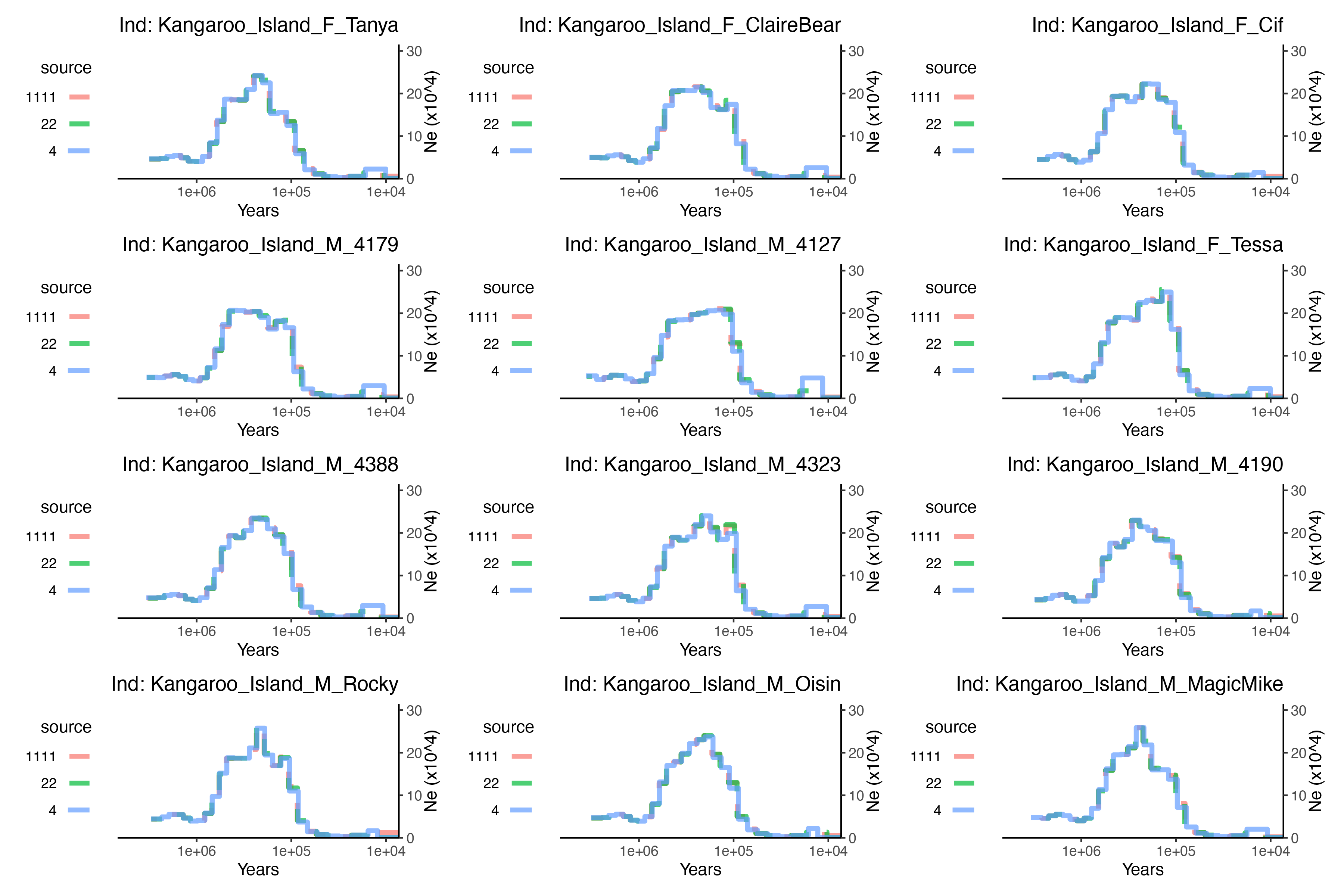
**

**Fig. S2 continued**

**
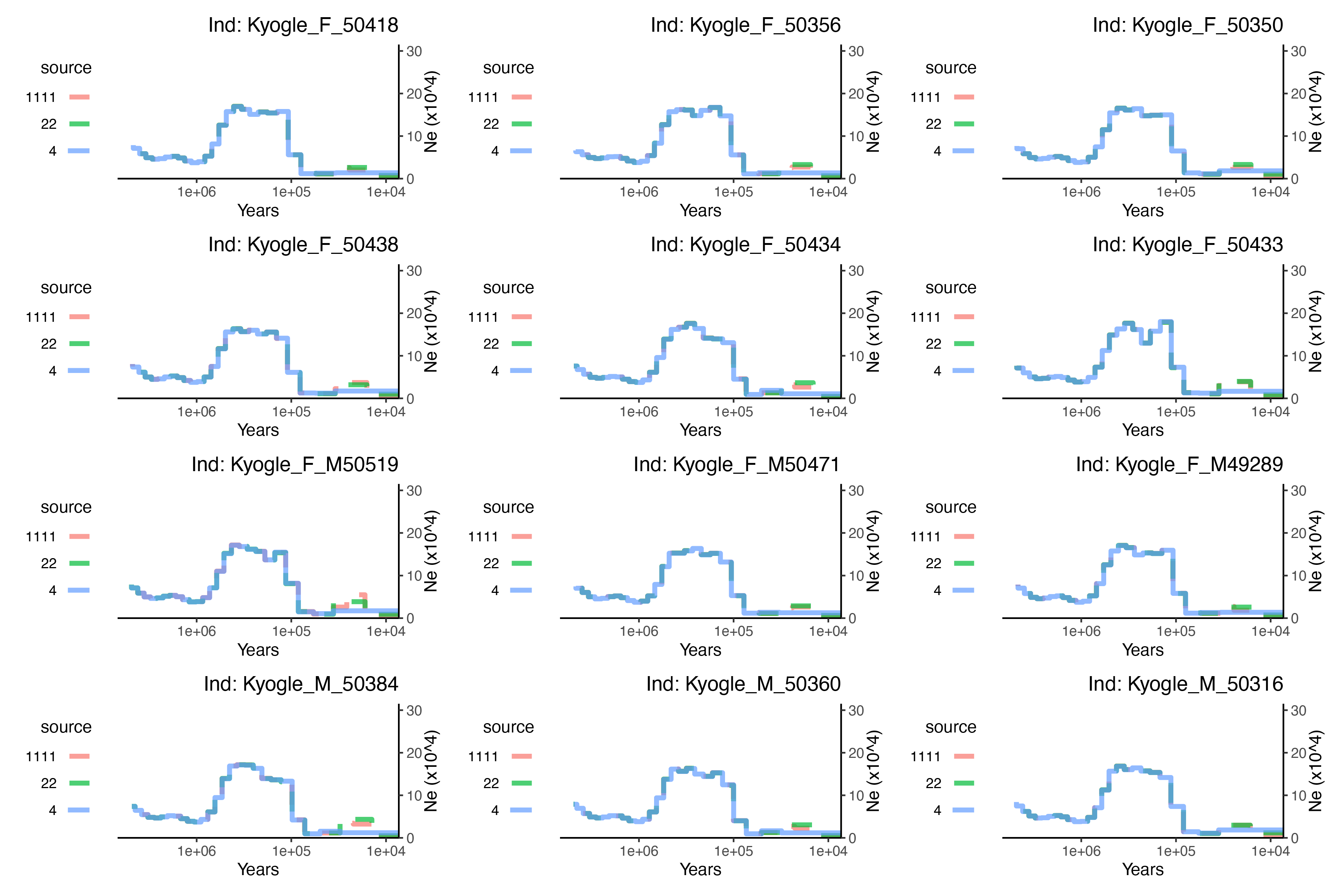

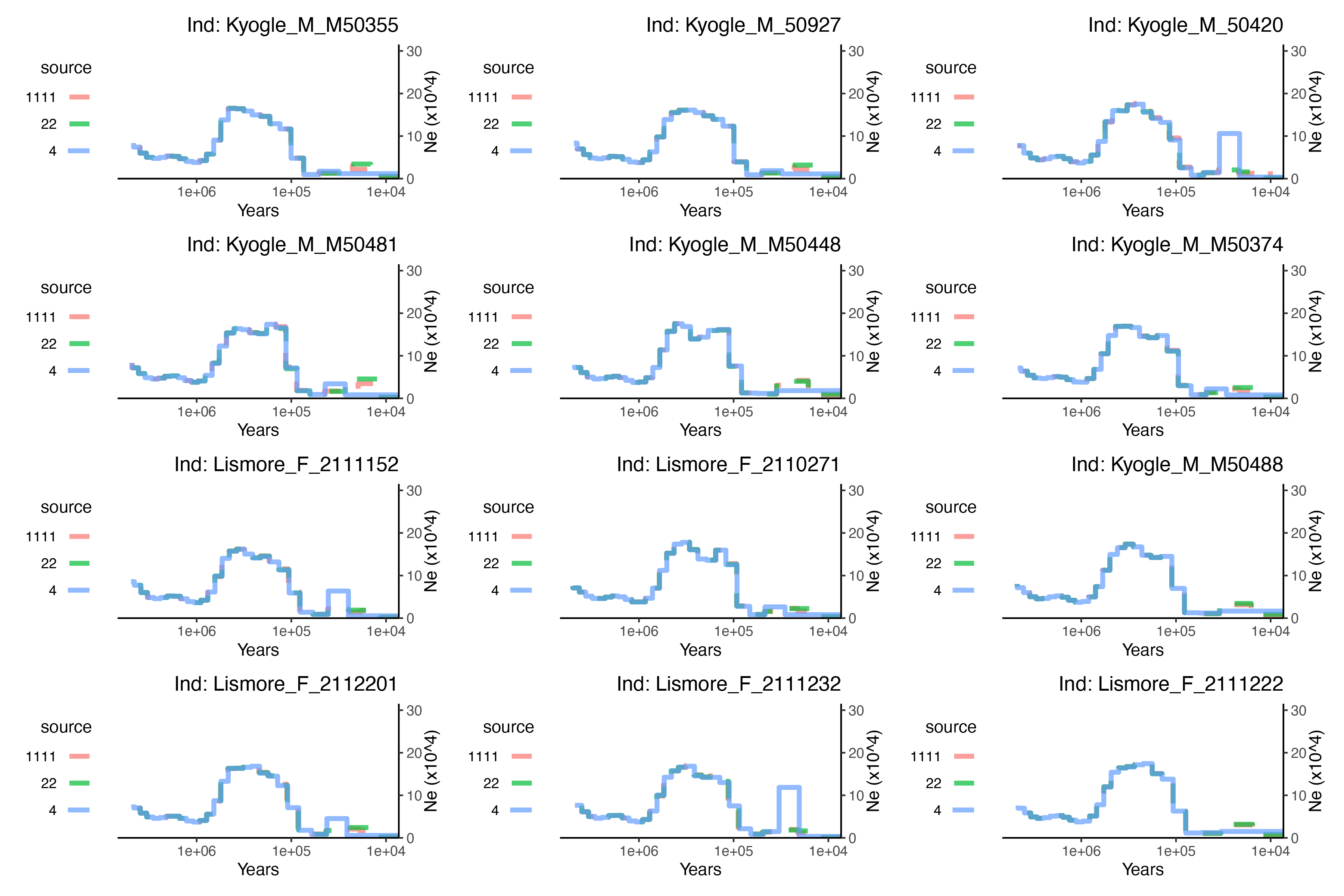
**

**Fig. S2 continued**

**
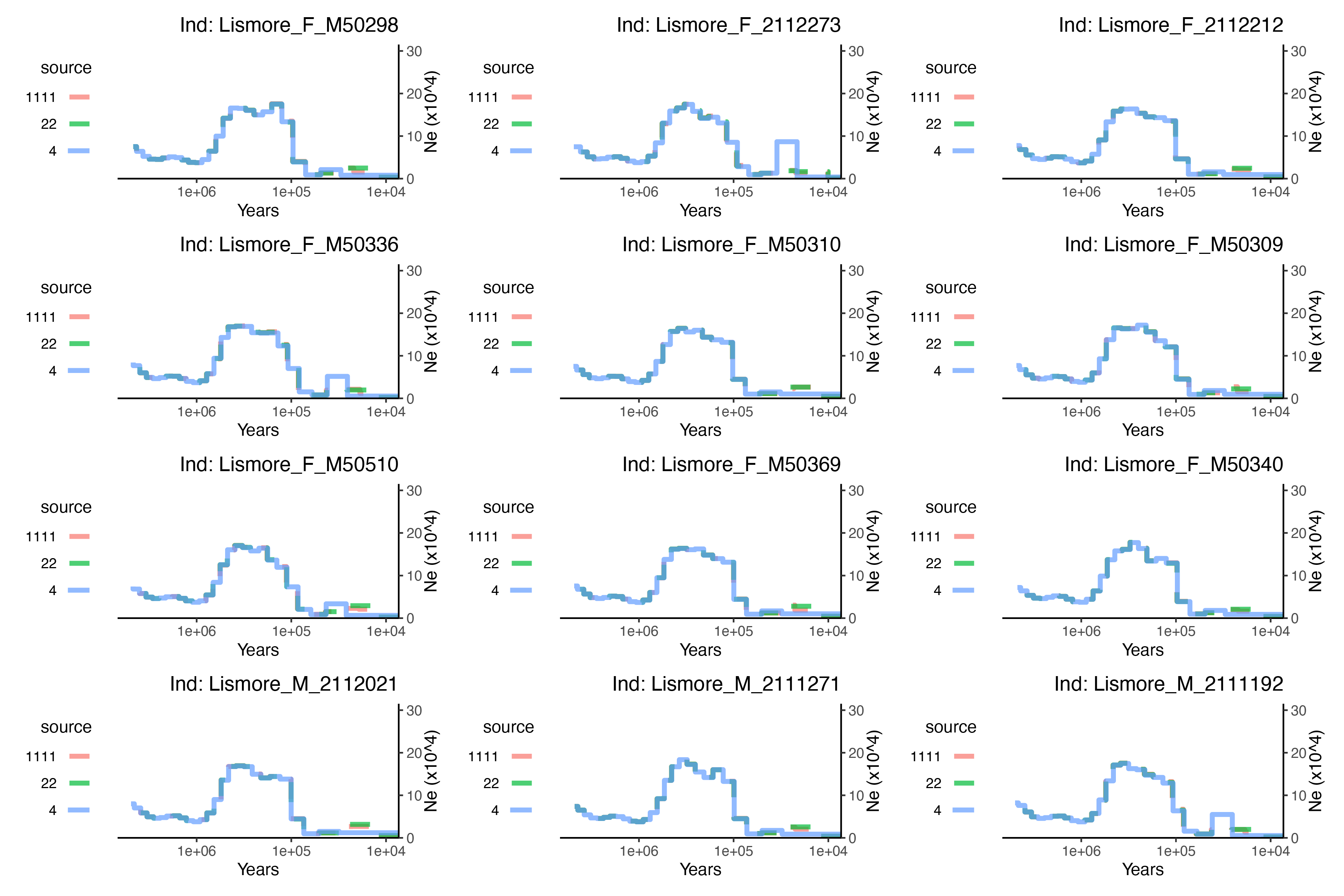

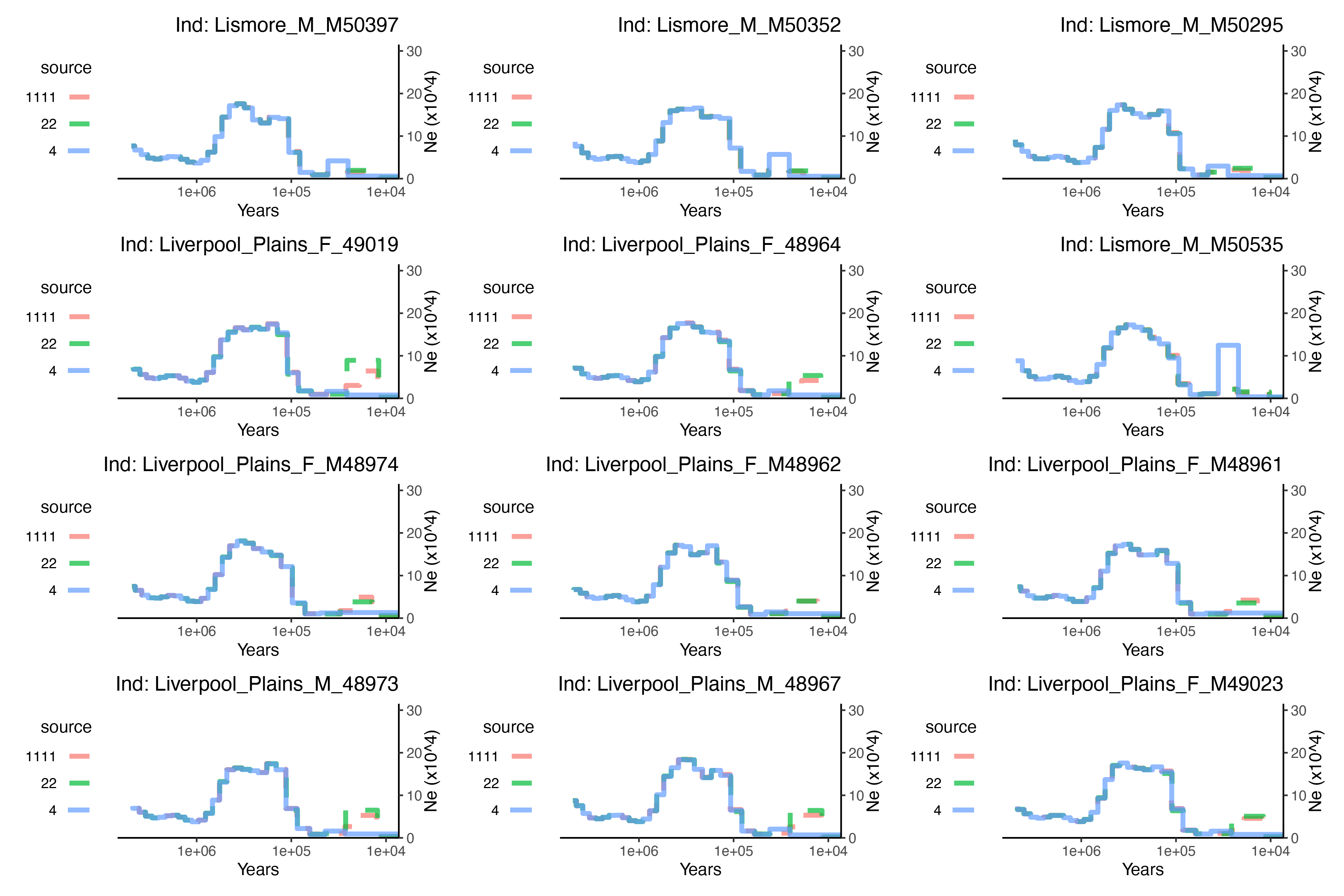
**

**Fig. S2 continued**

**
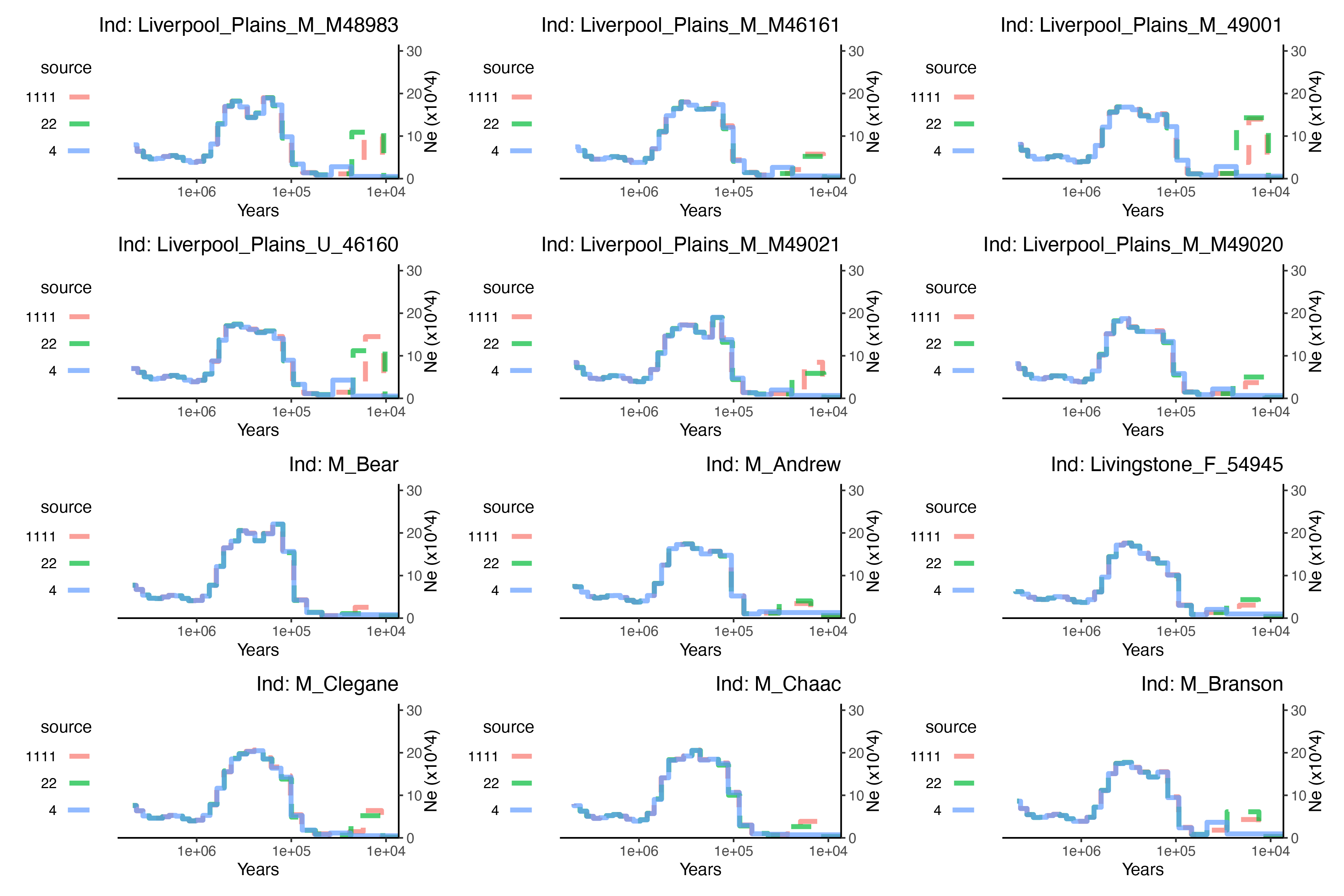

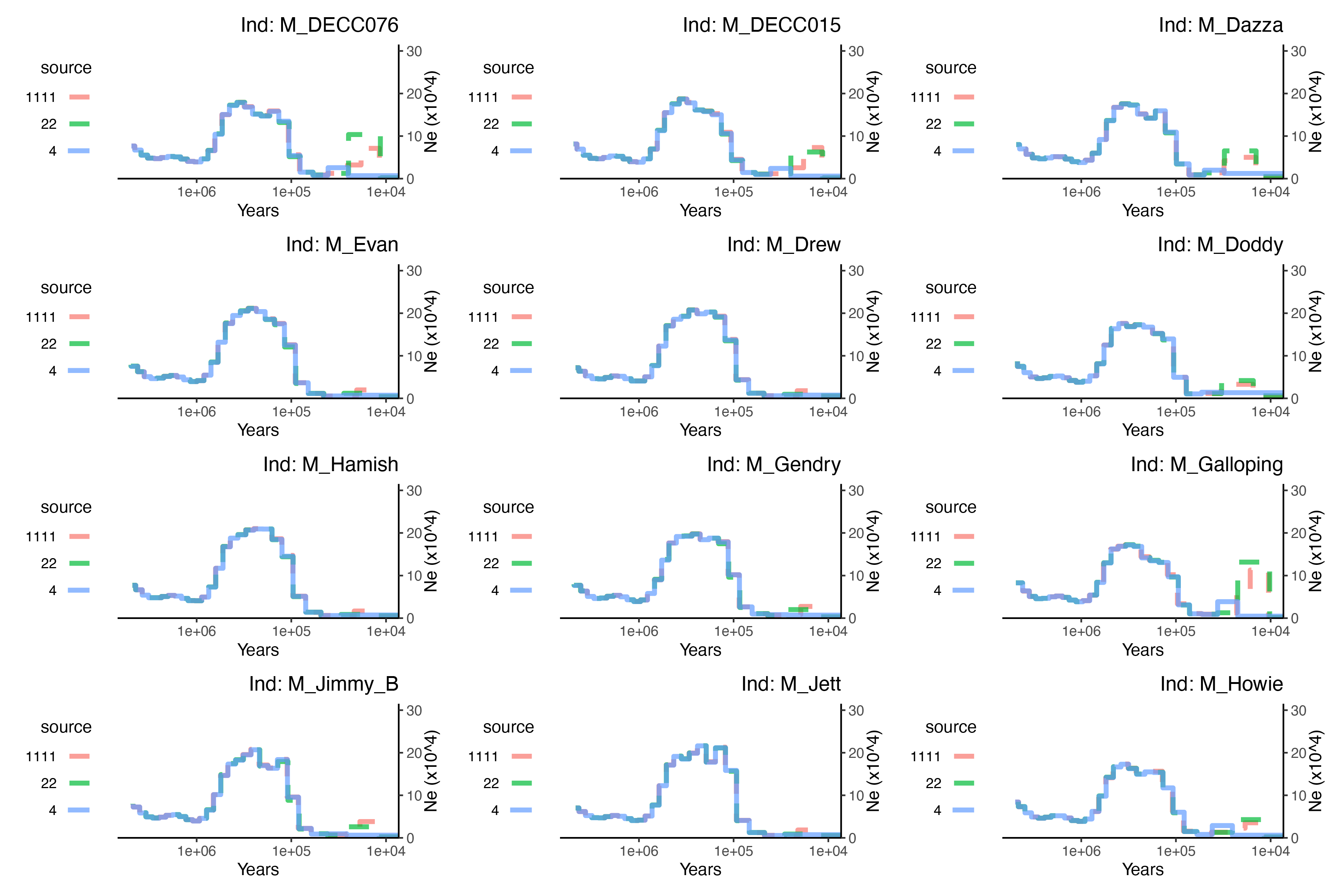
**

**Fig. S2 continued**

**
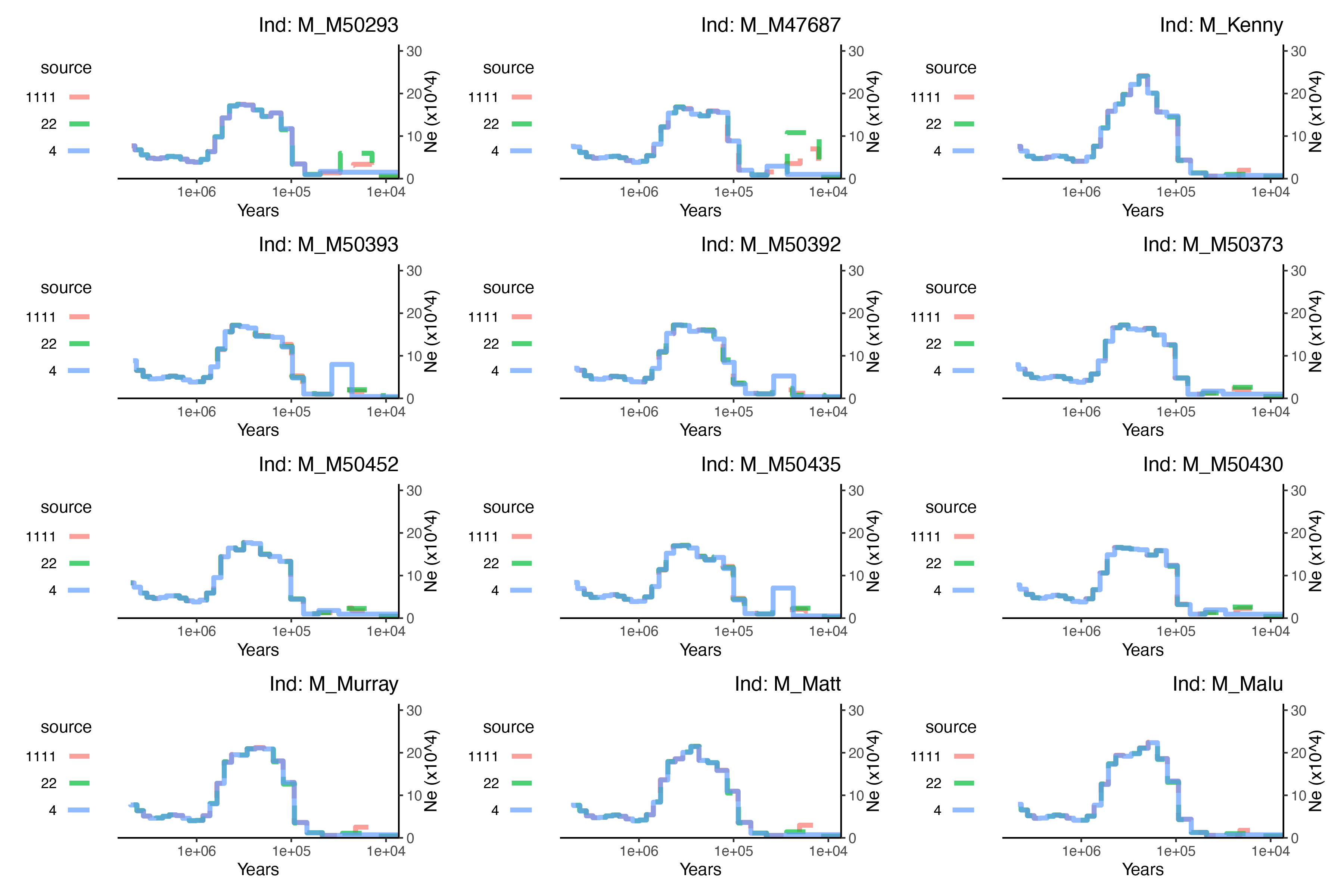

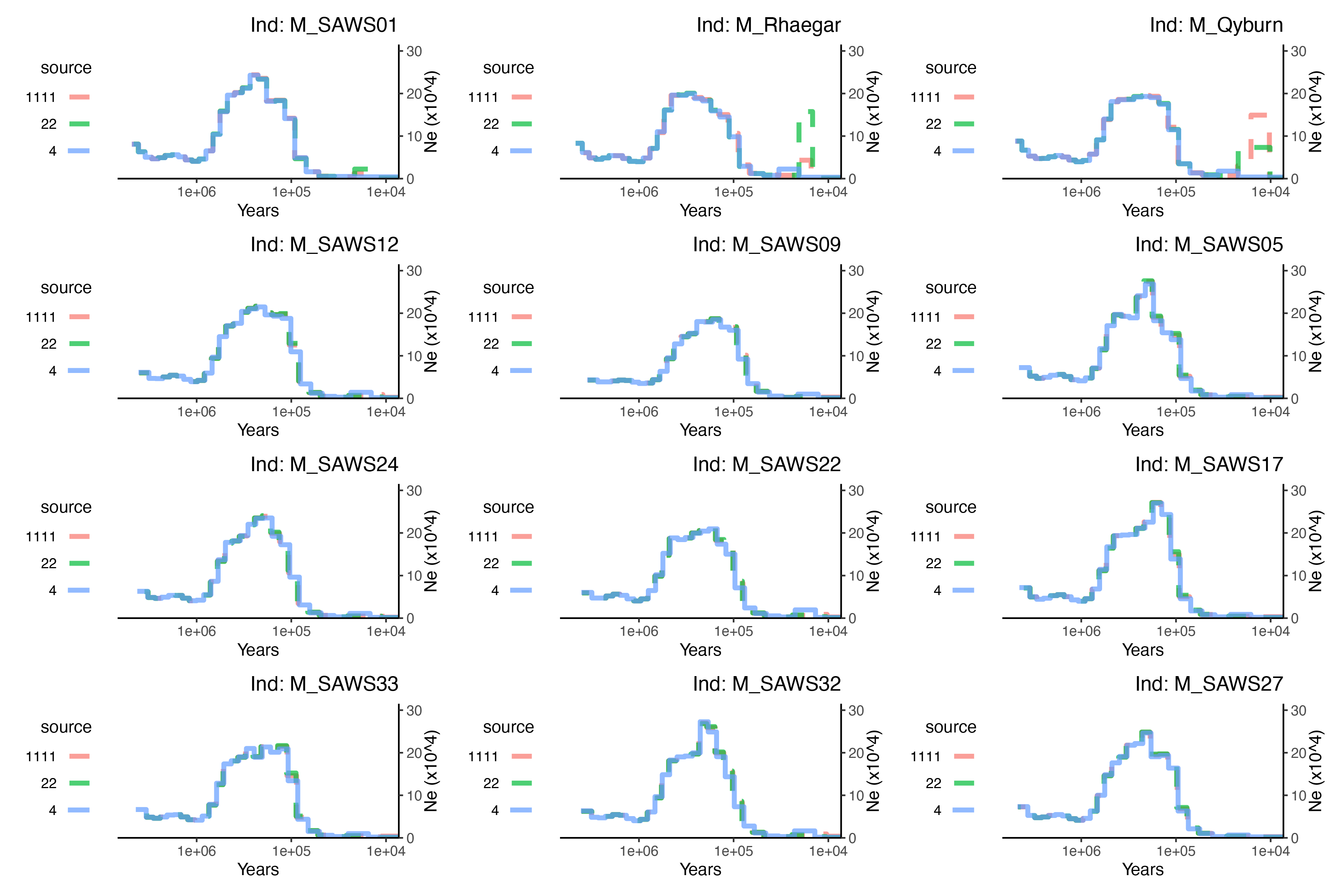
**

**Fig. S2 continued**

**
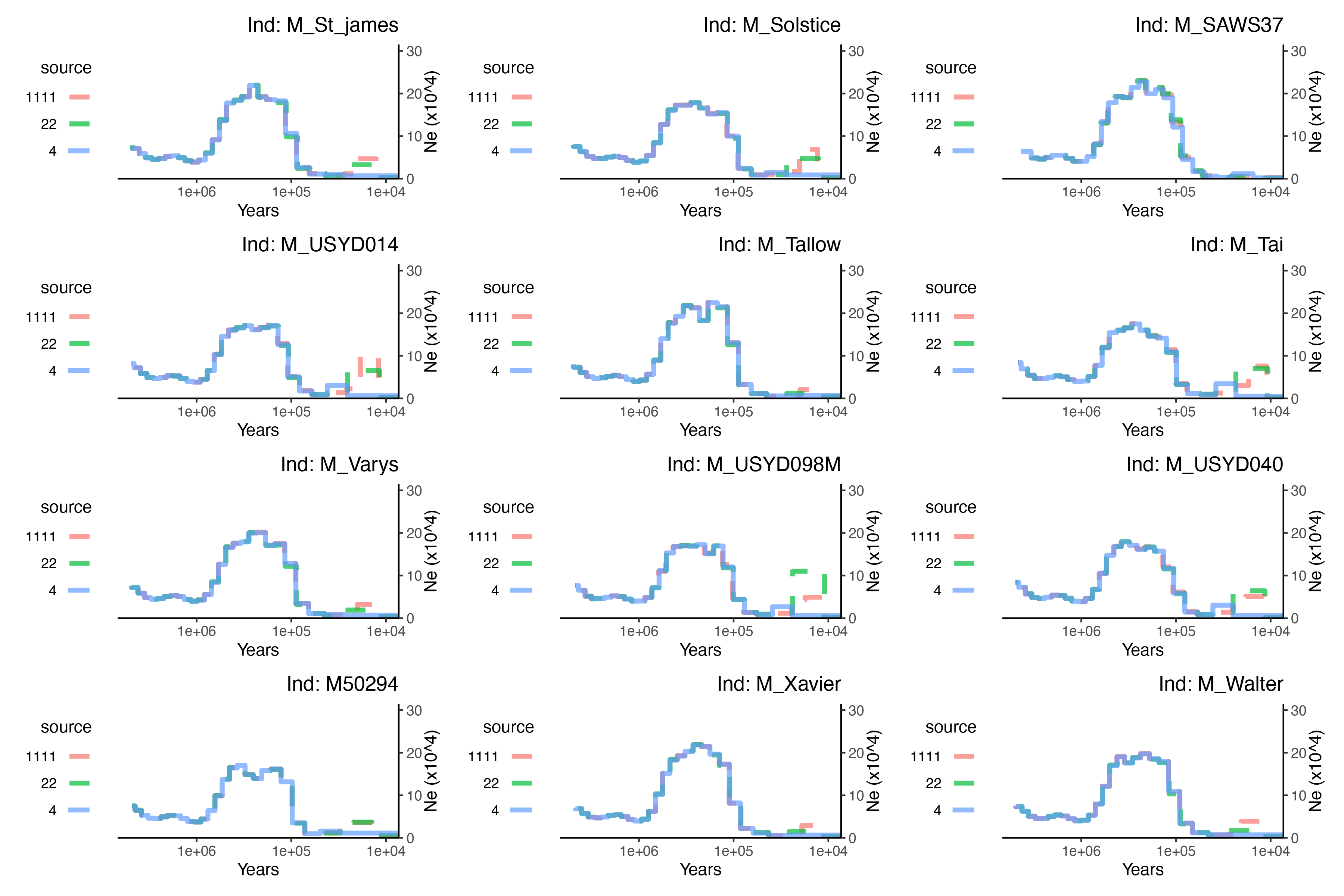

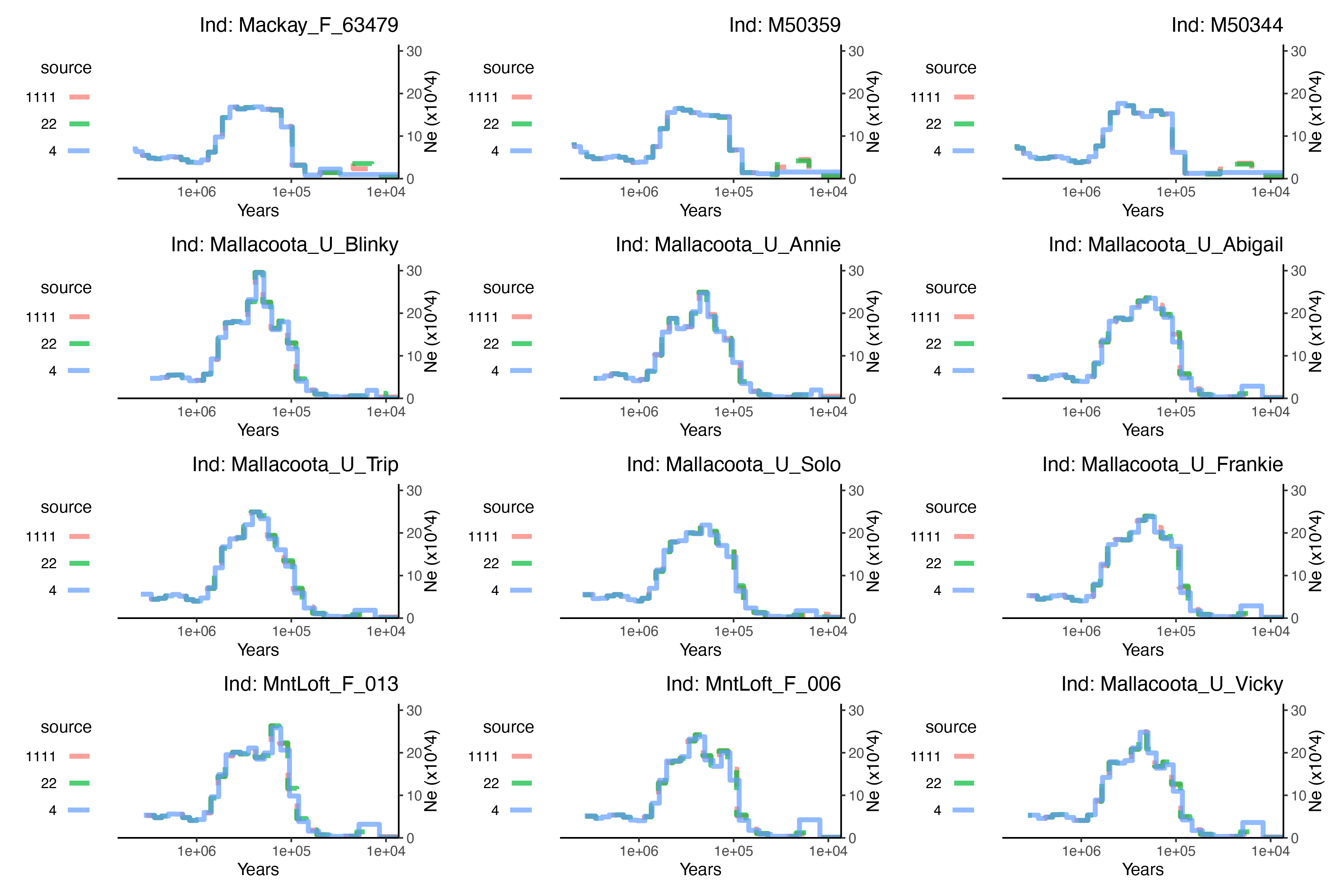
**

**Fig. S2 continued**

**
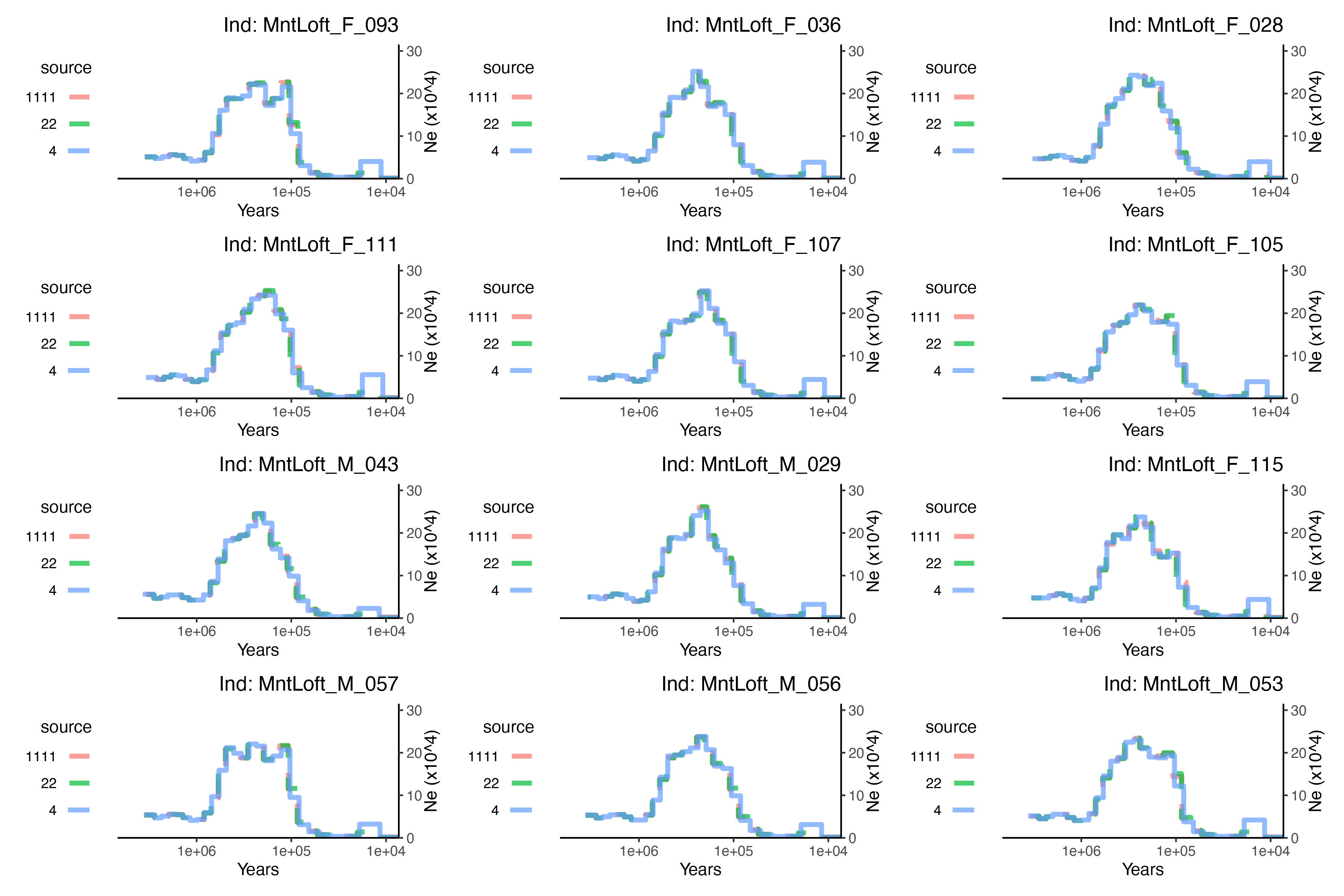

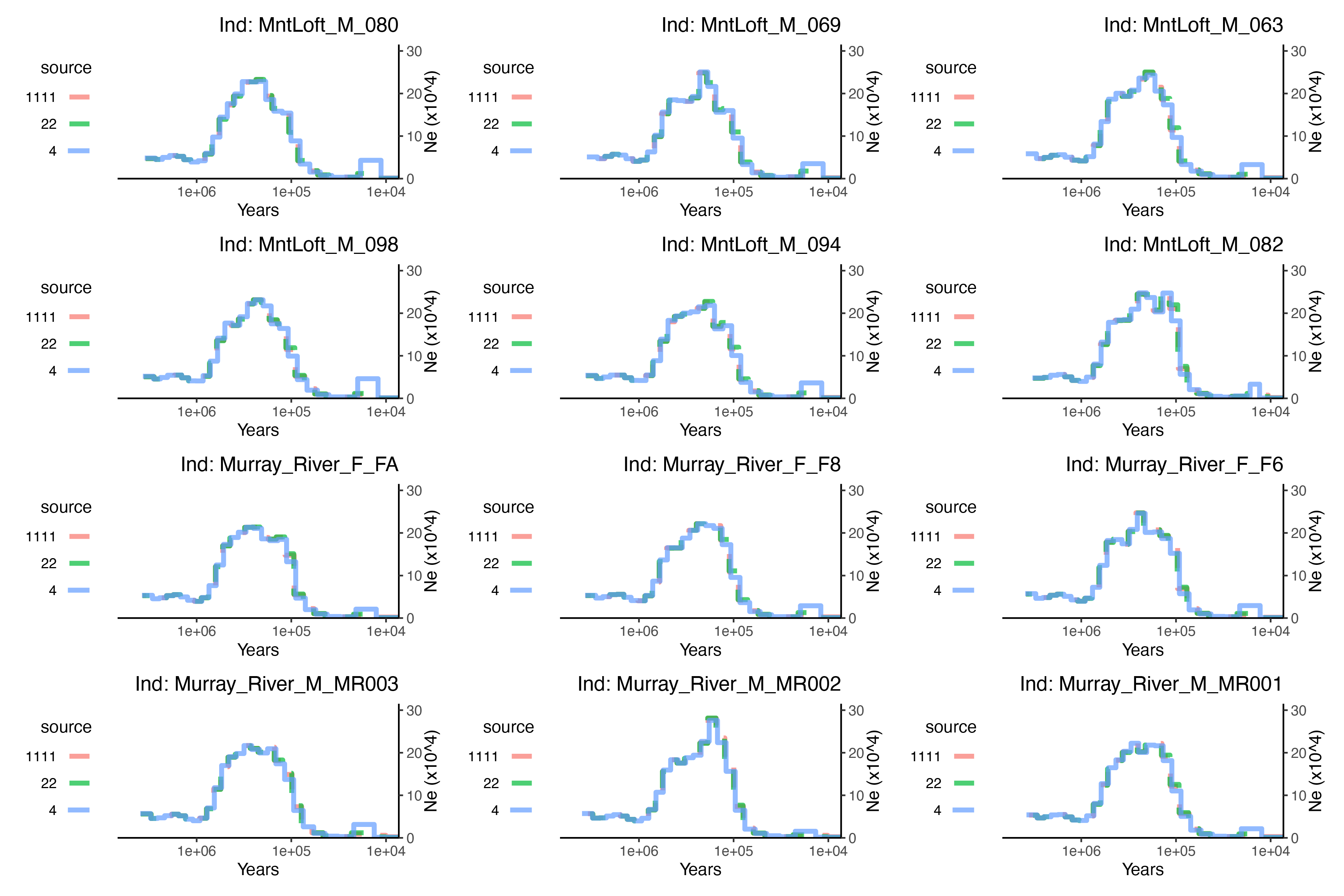
**

**Fig. S2 continued**

**
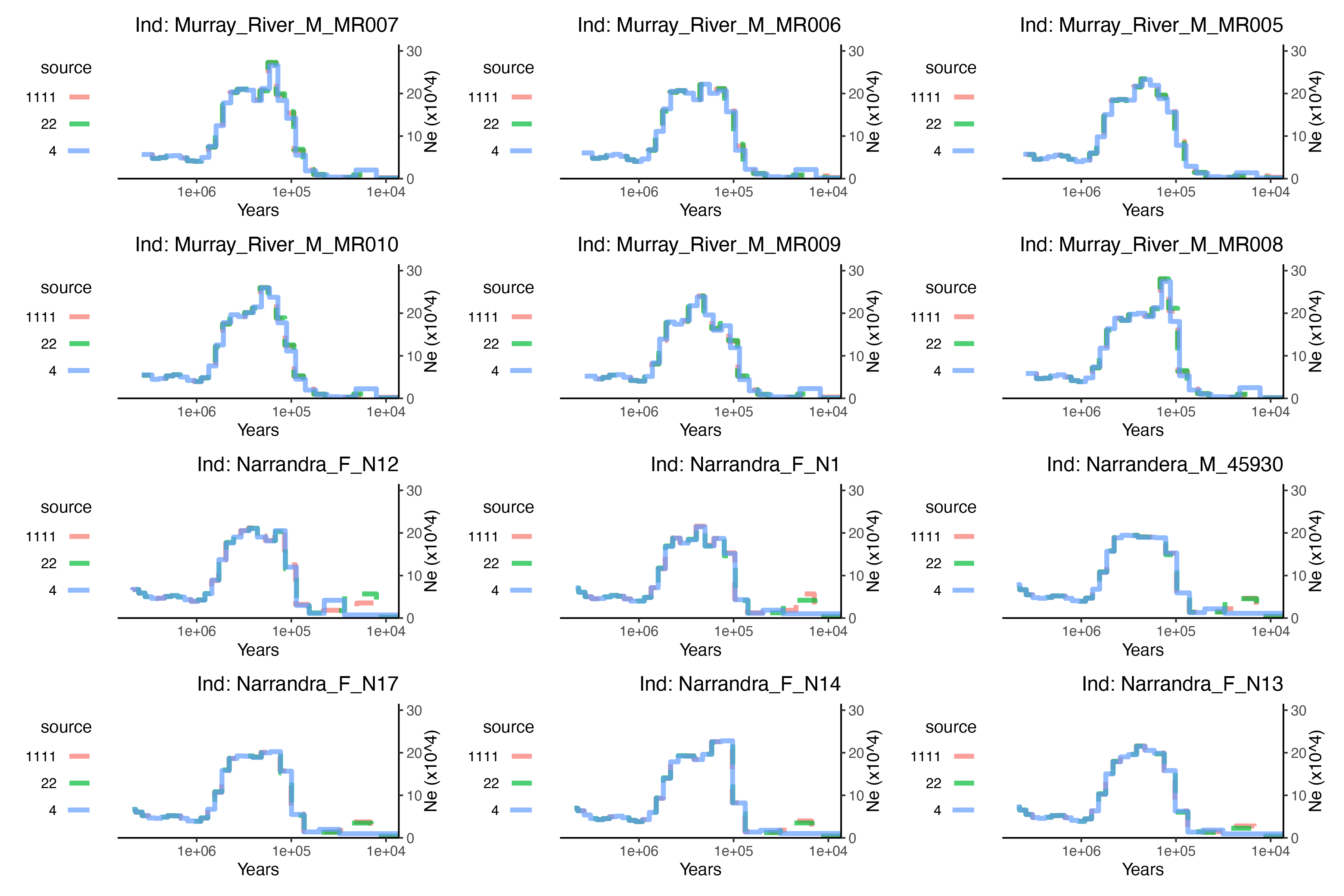

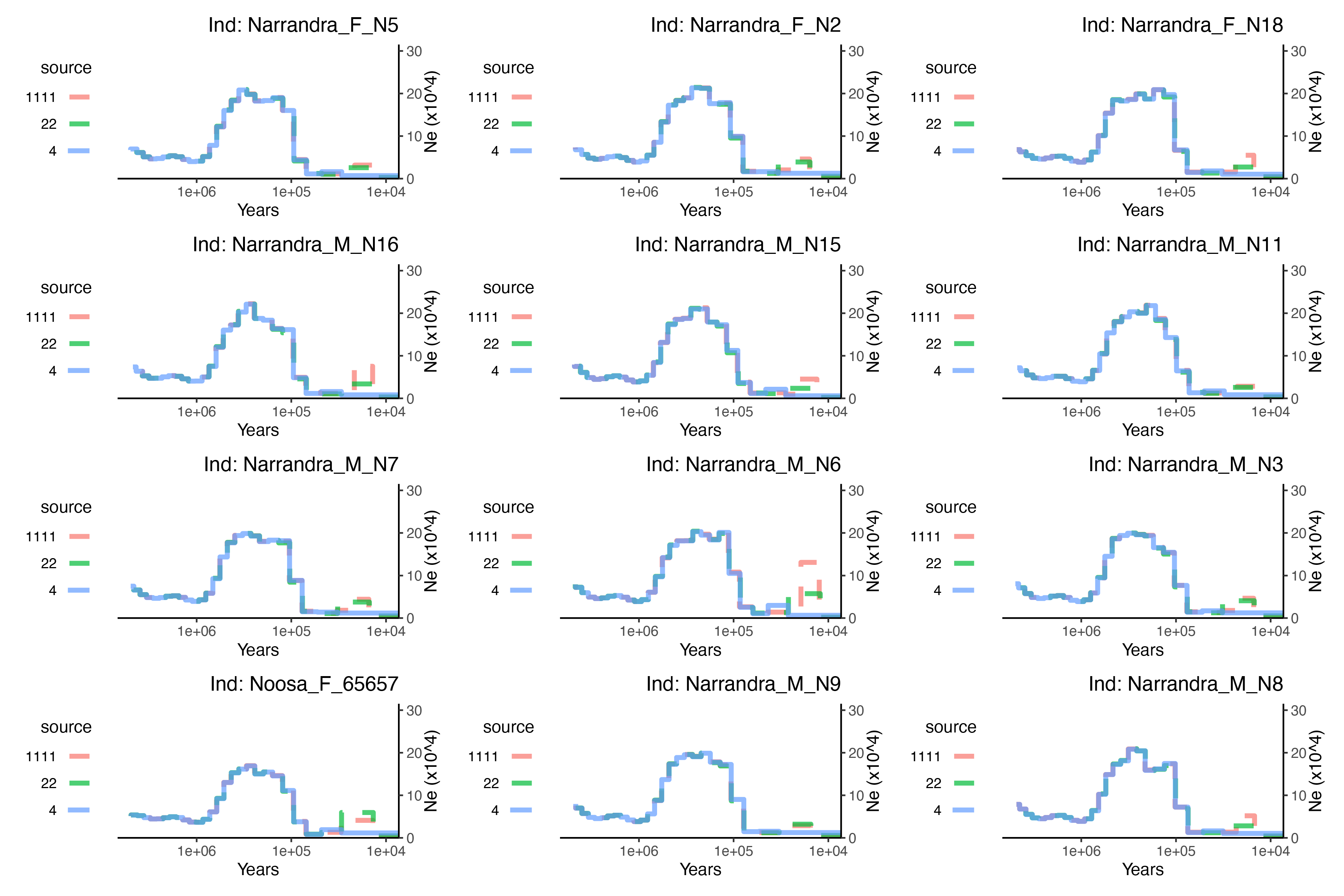
**

**Fig. S2 continued**

**
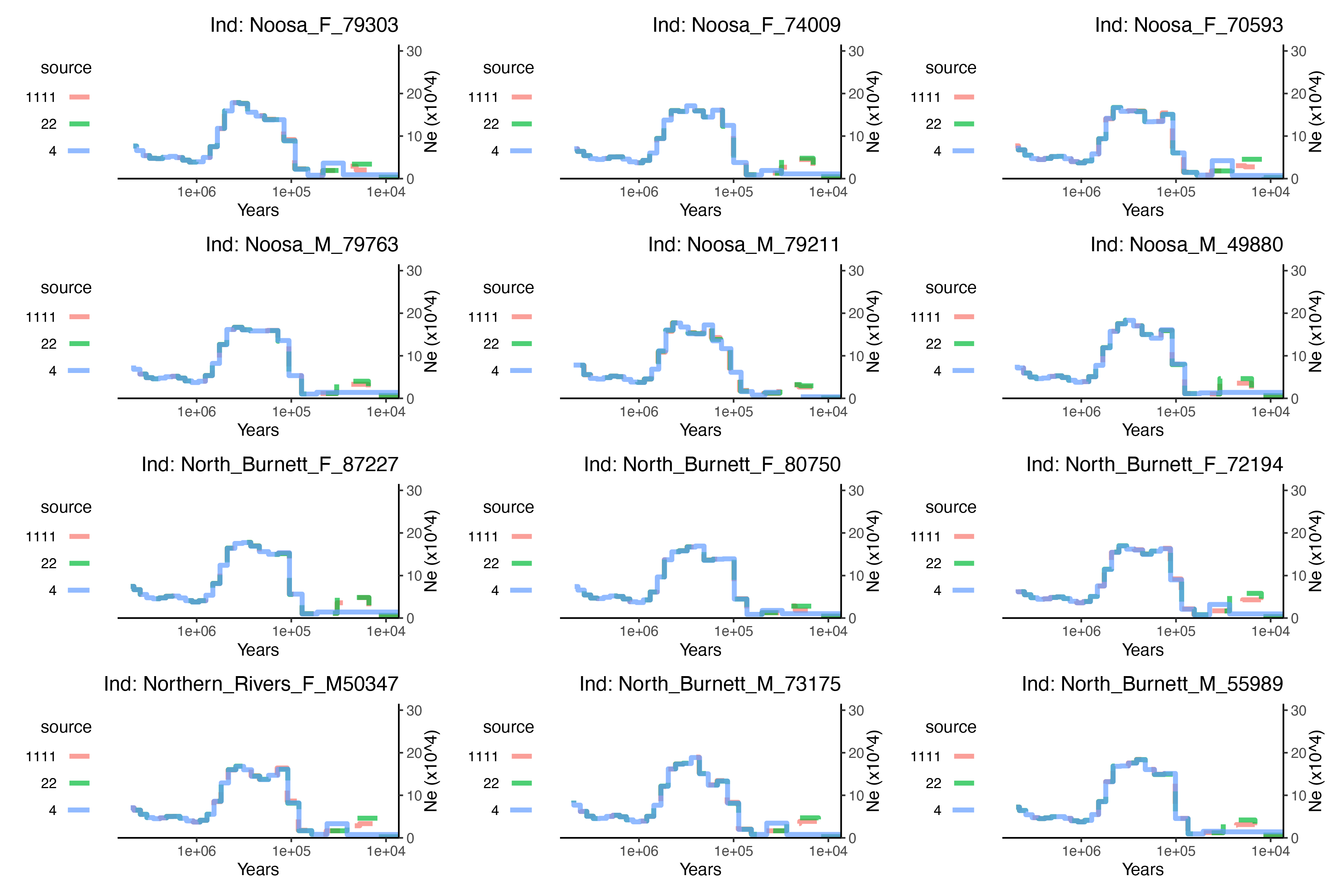

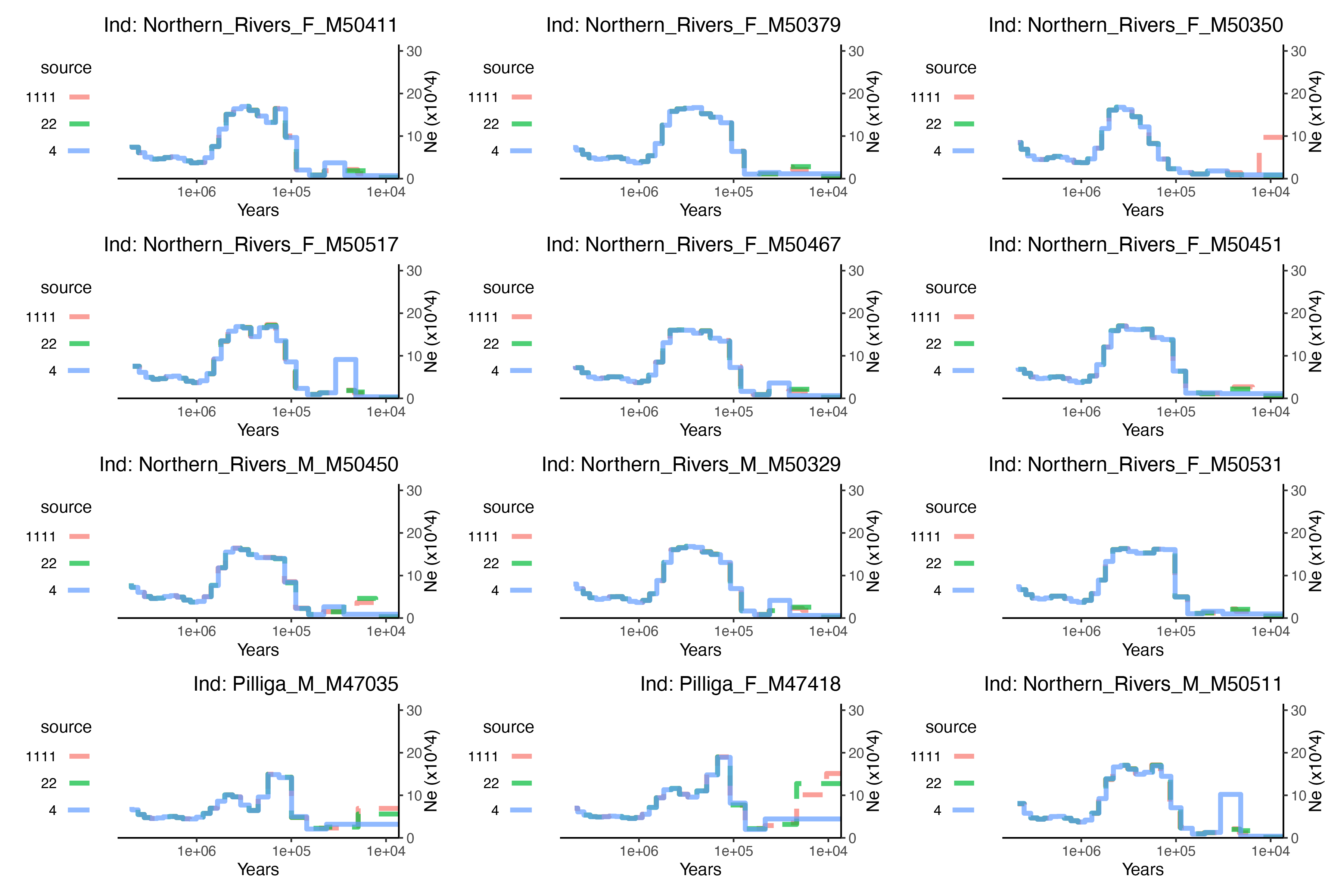
**

**Fig. S2 continued**

**
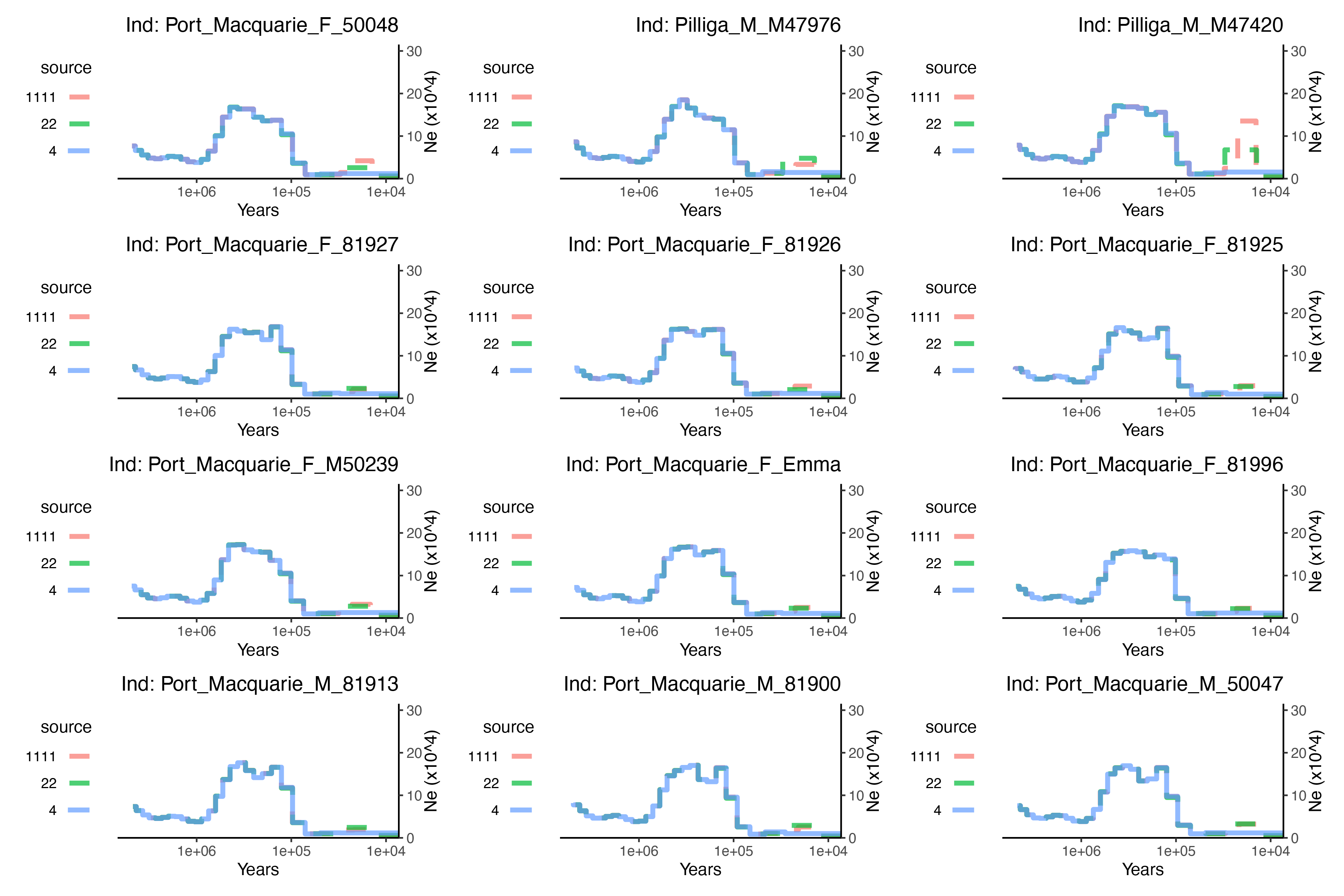

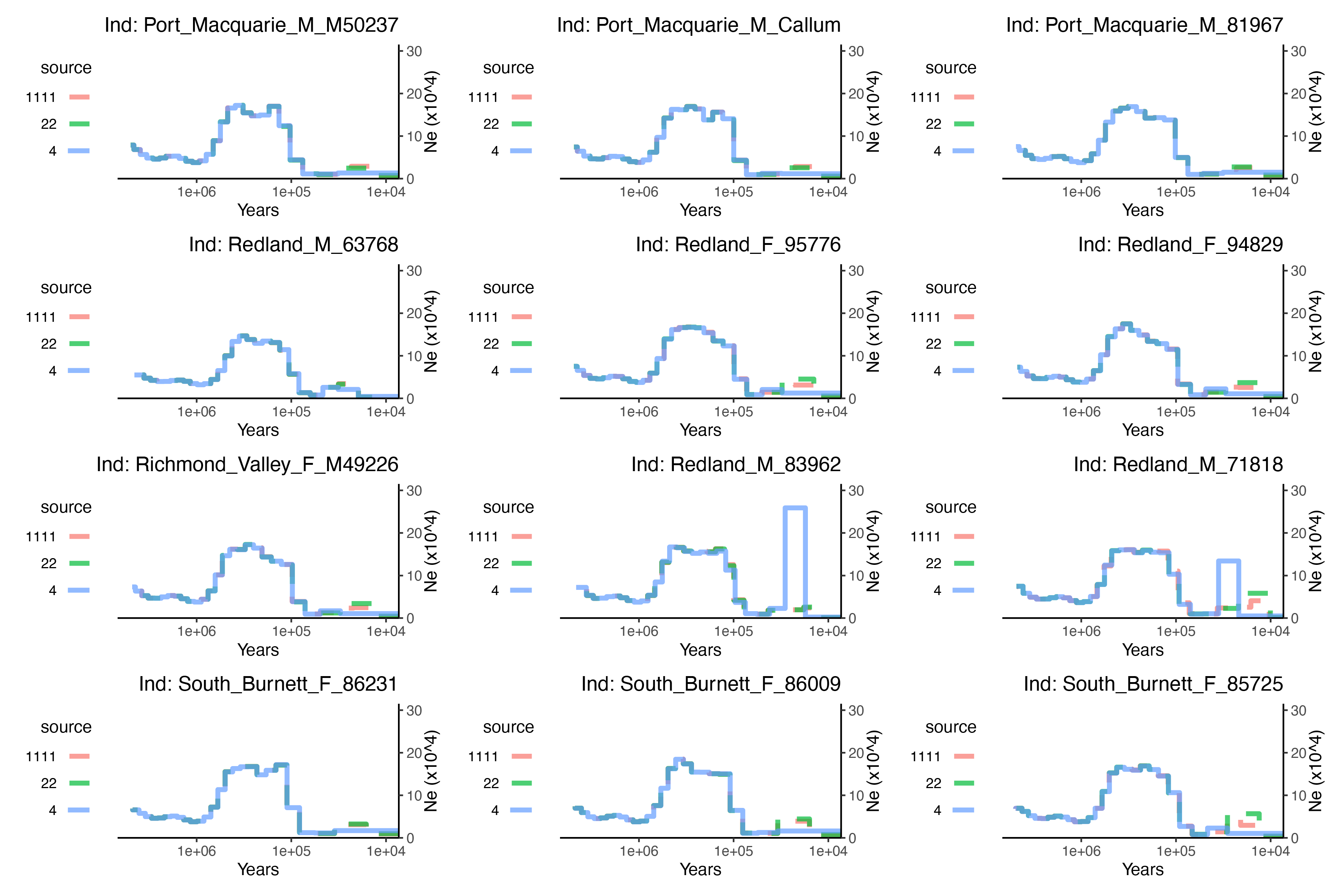
**

**Fig. S2 continued**

**
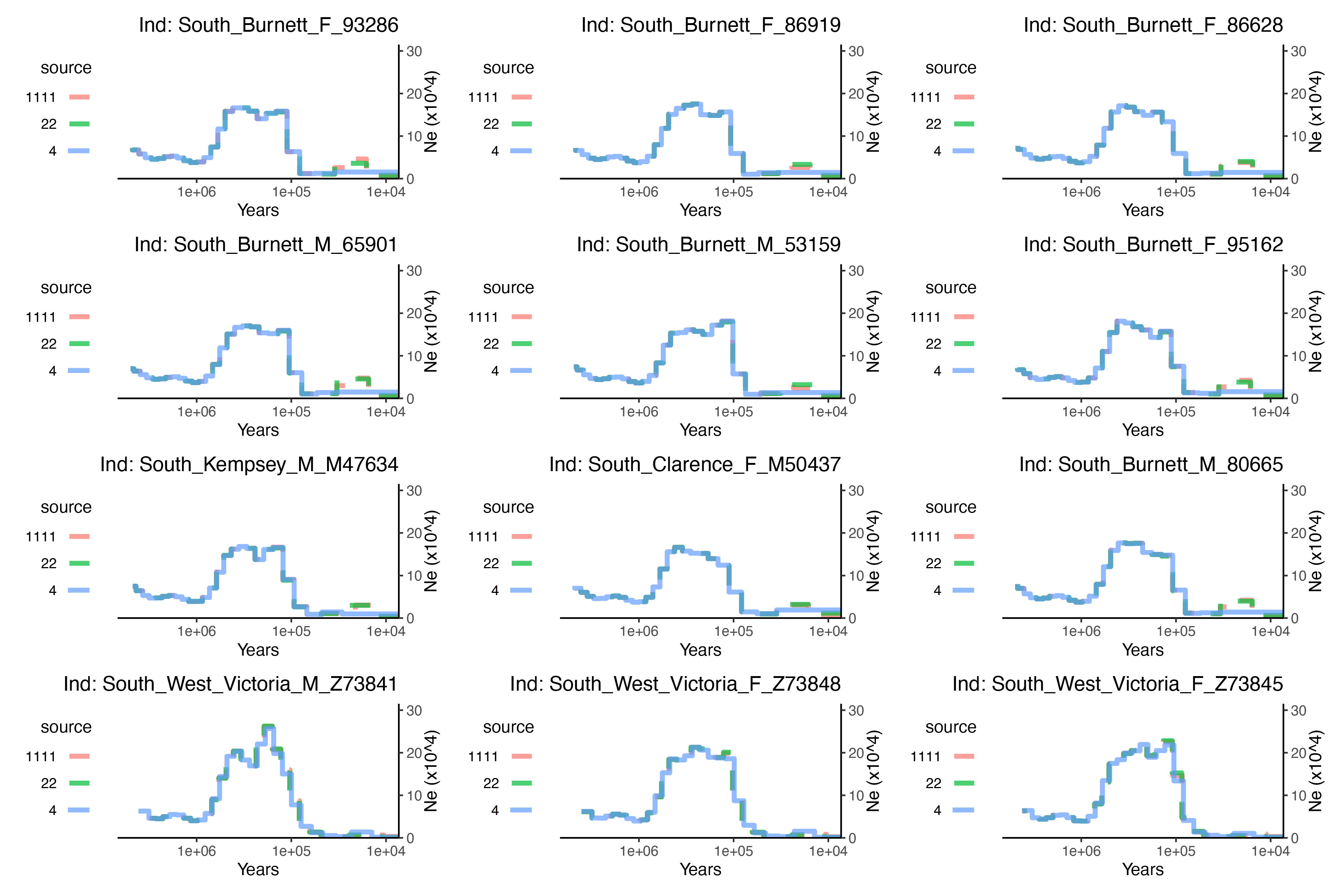

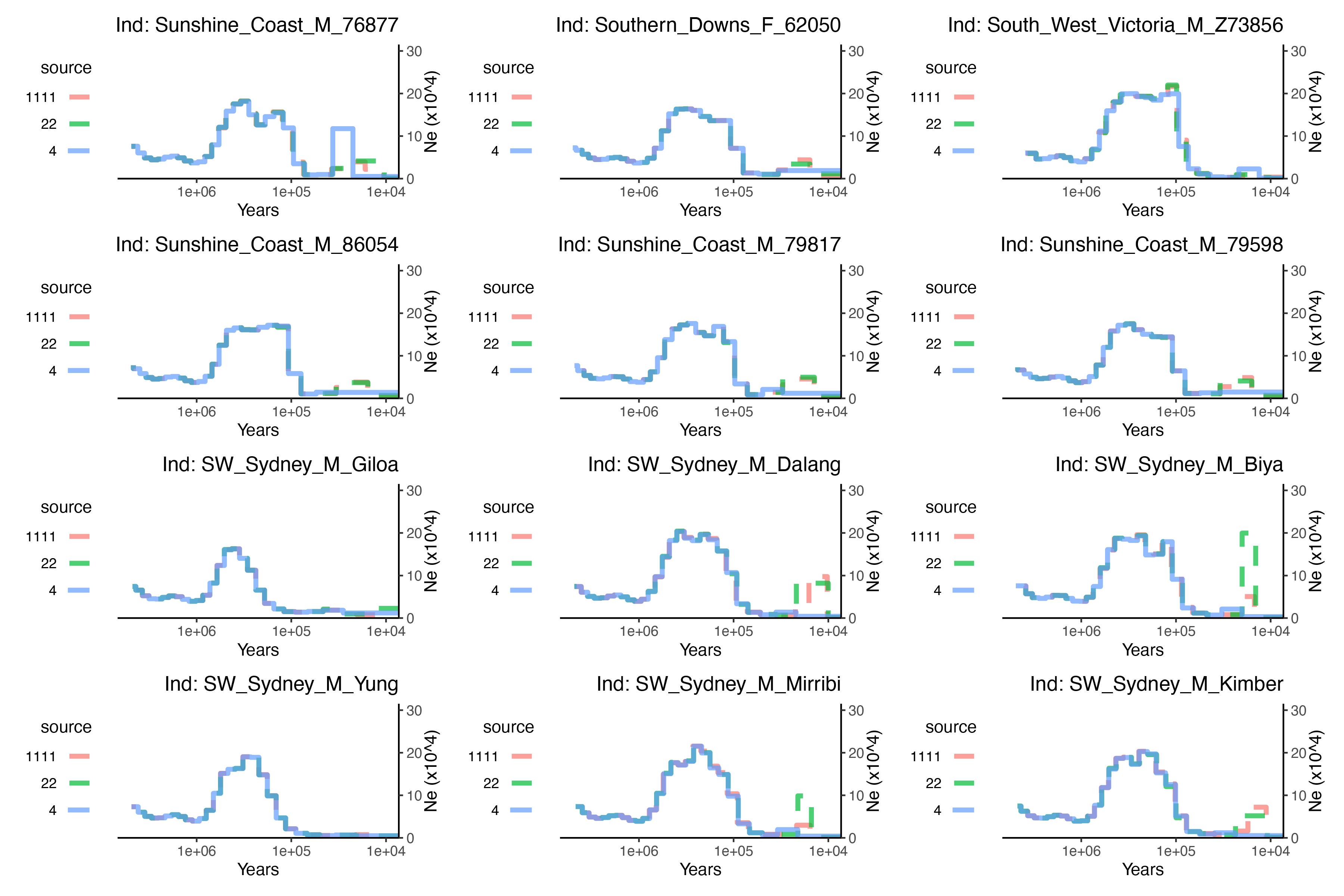
**

**Fig. S2 continued**

**
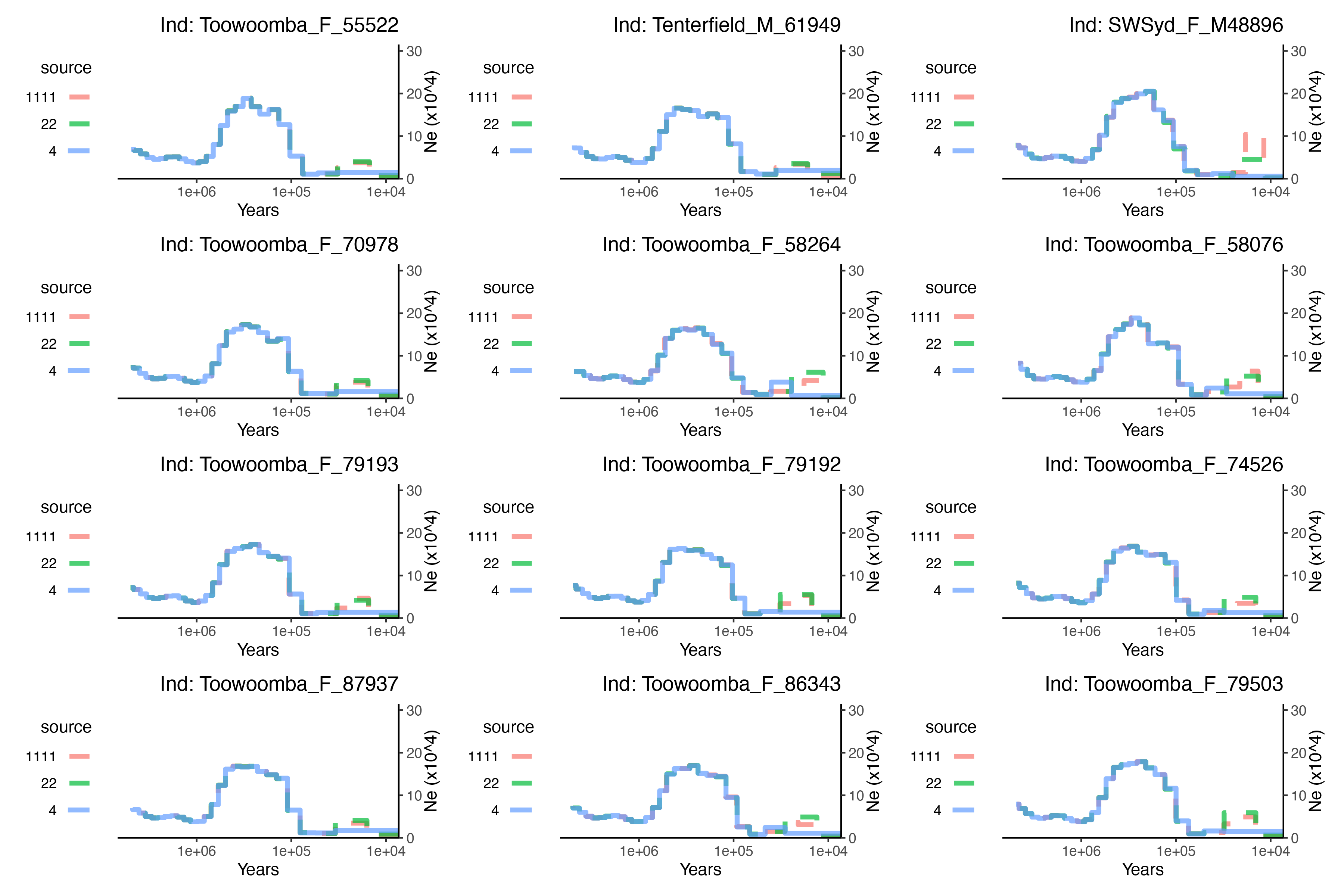

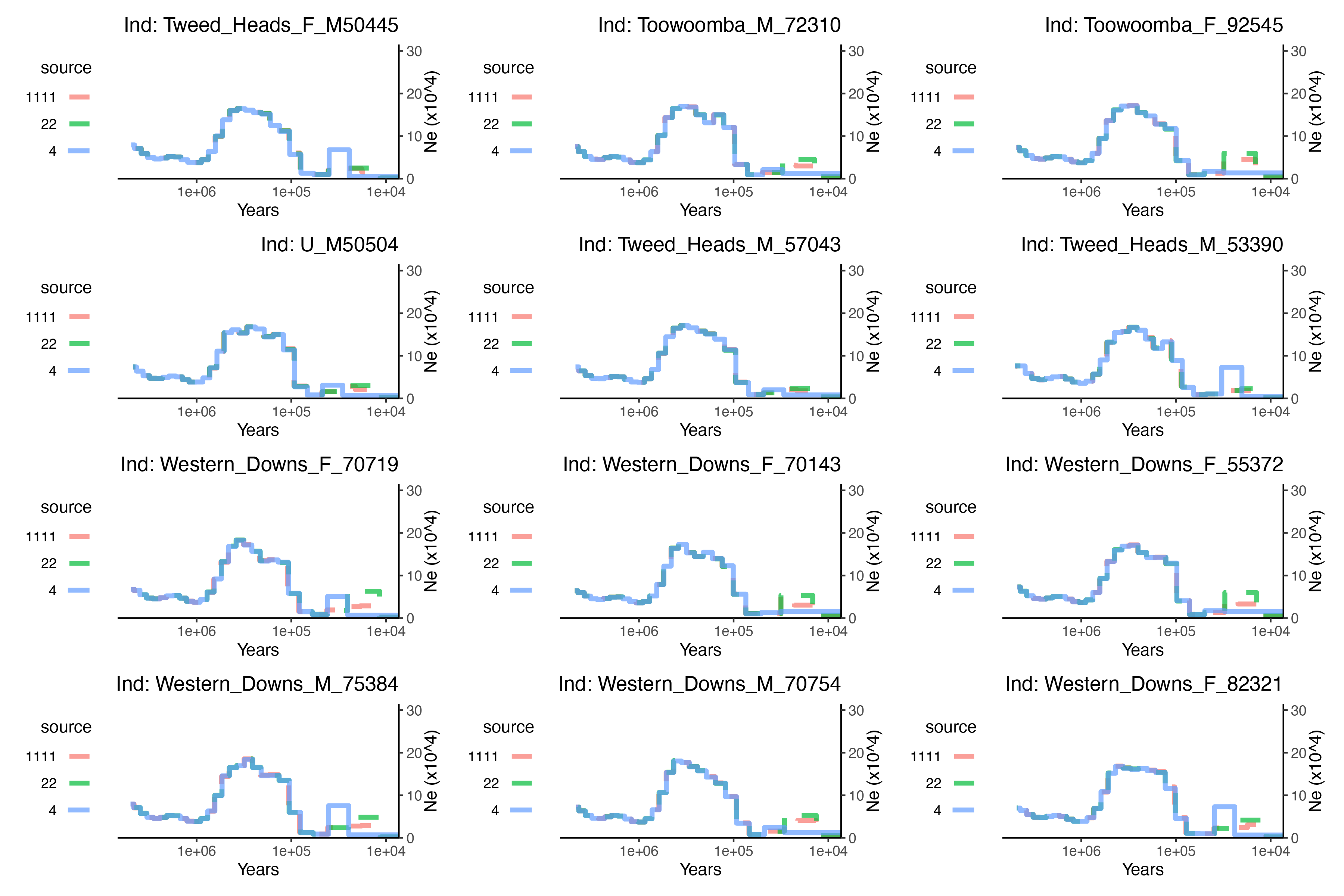
**

**Fig. S2 continued**

**
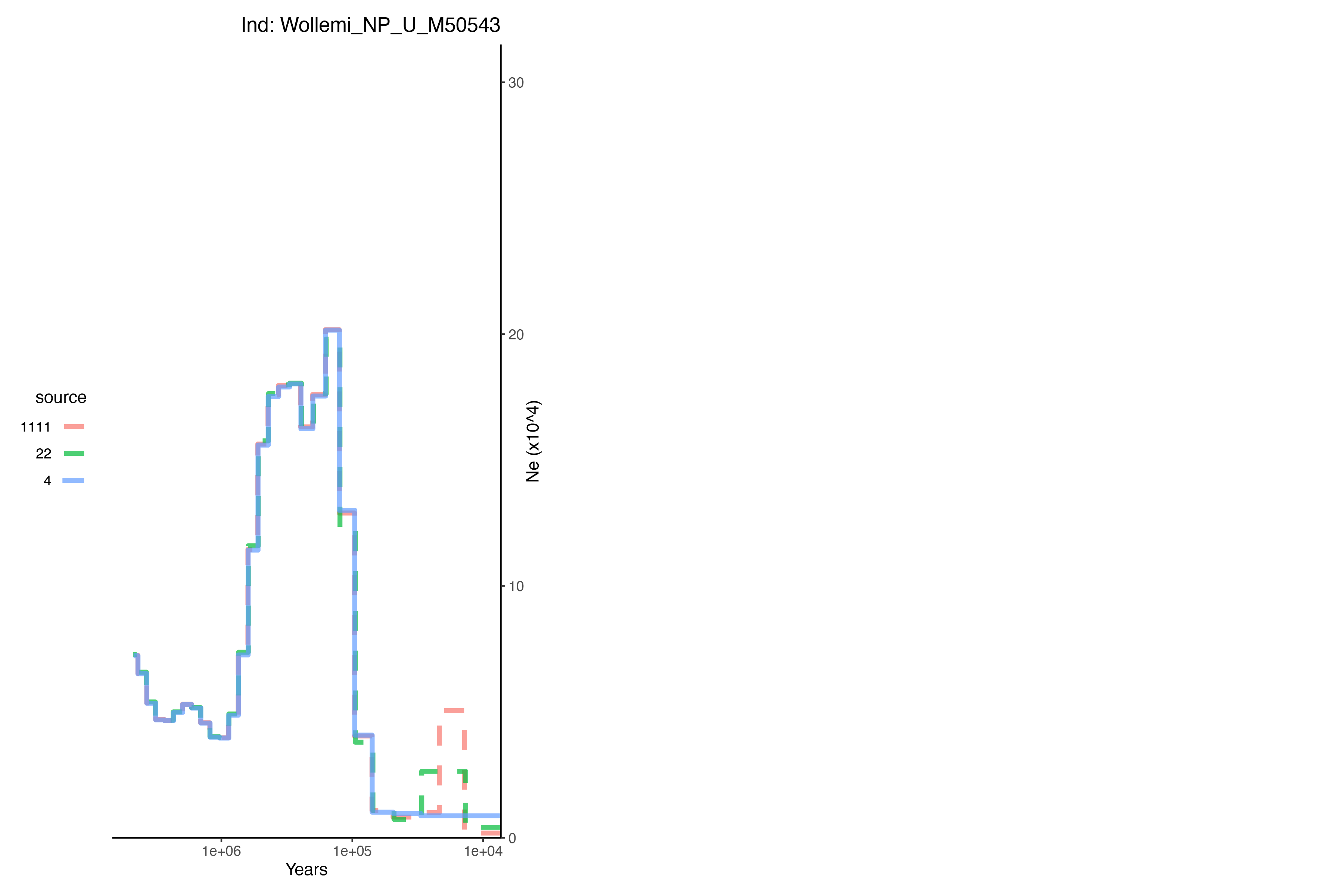
**

**Fig. S2 continued**


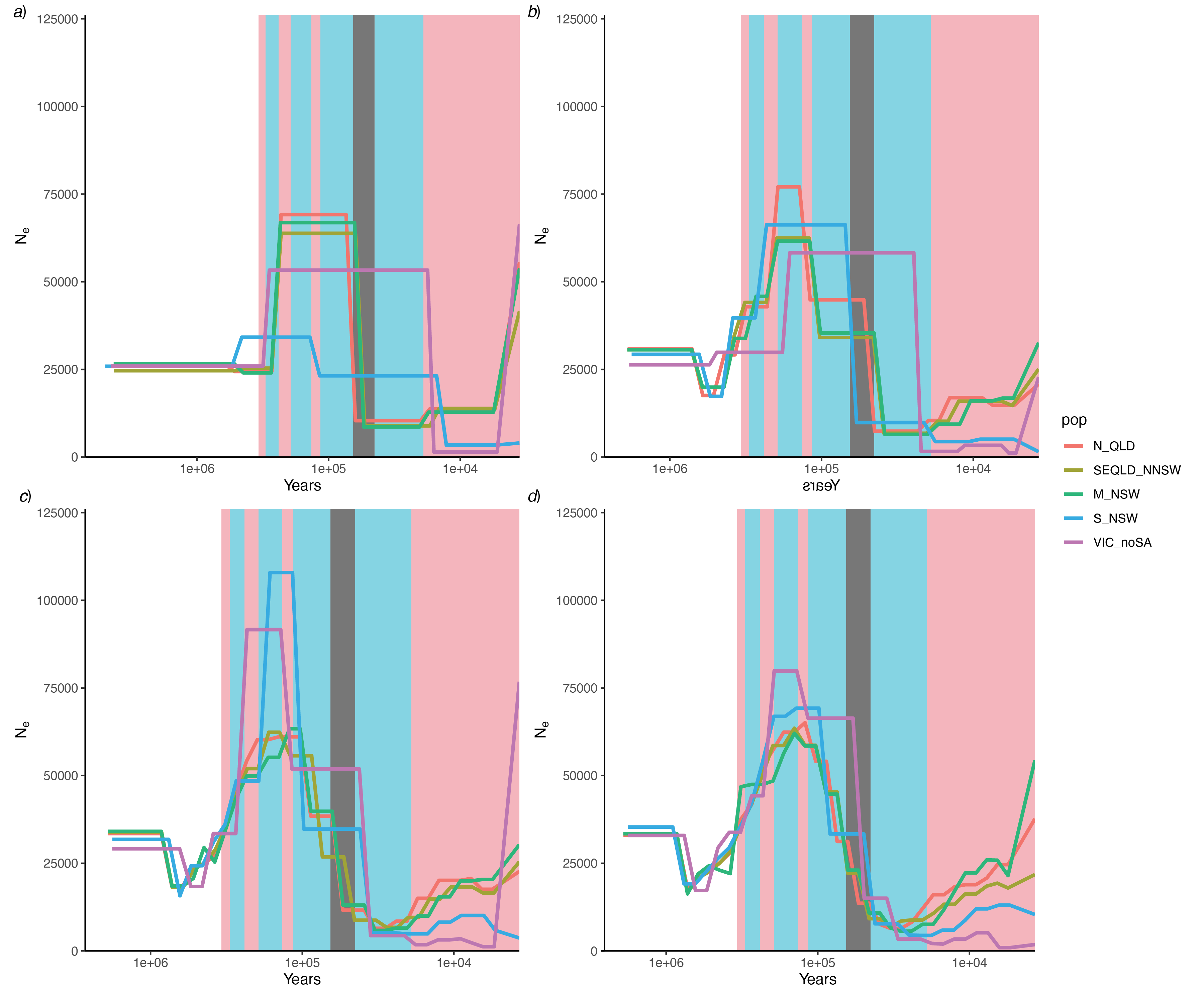


**Fig. S3**


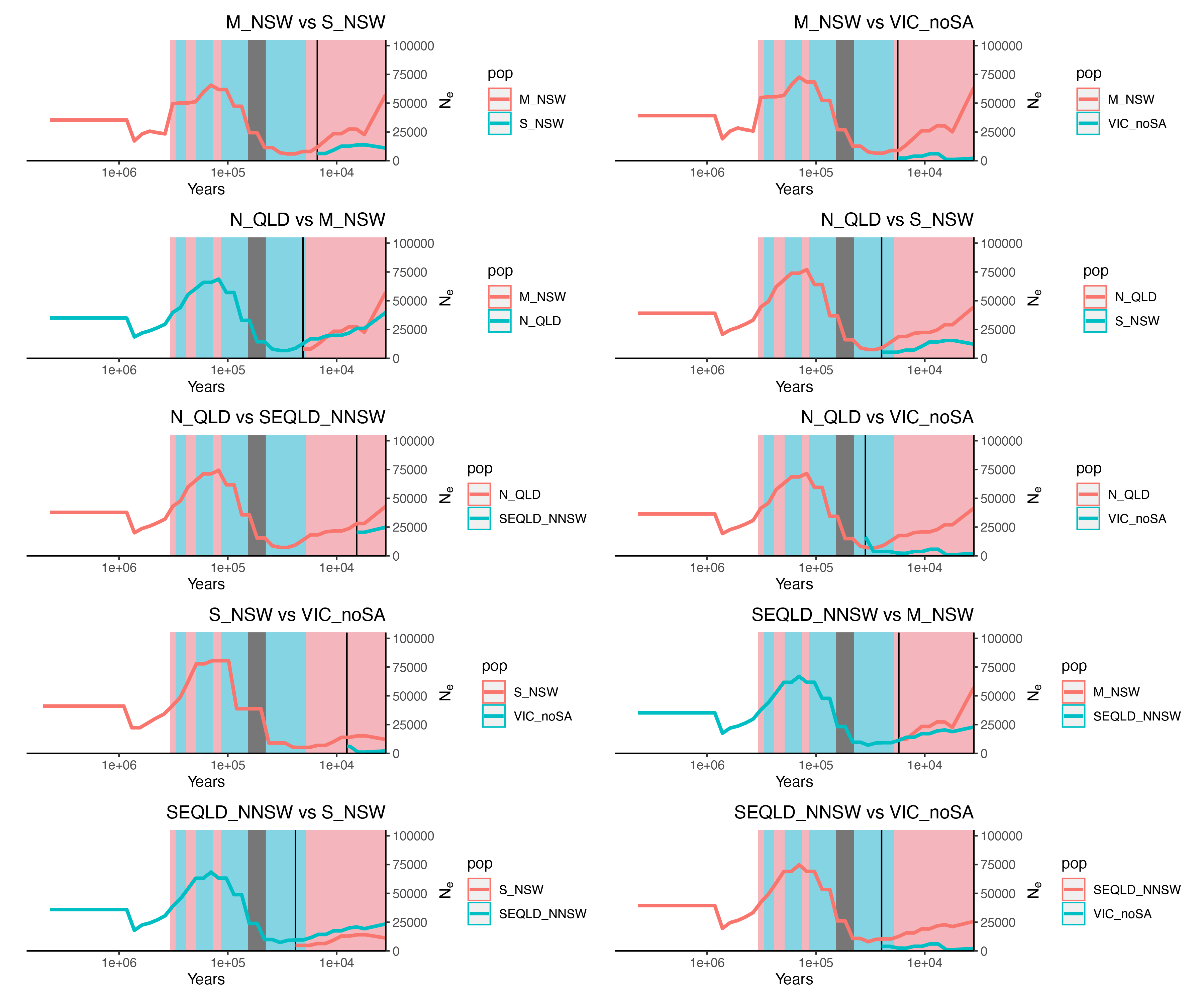


**Fig. S4**

**
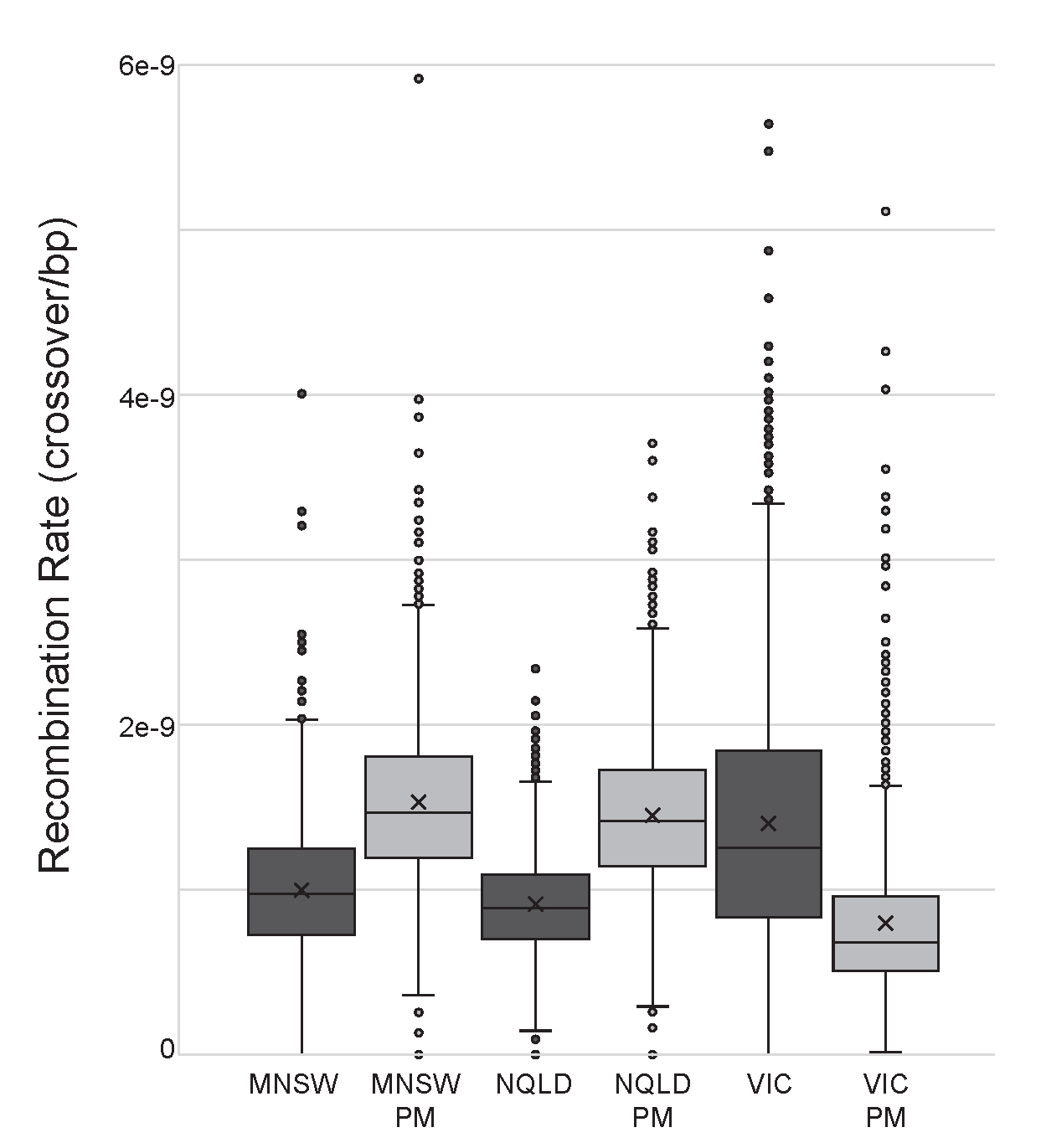
**

**Fig. S5**


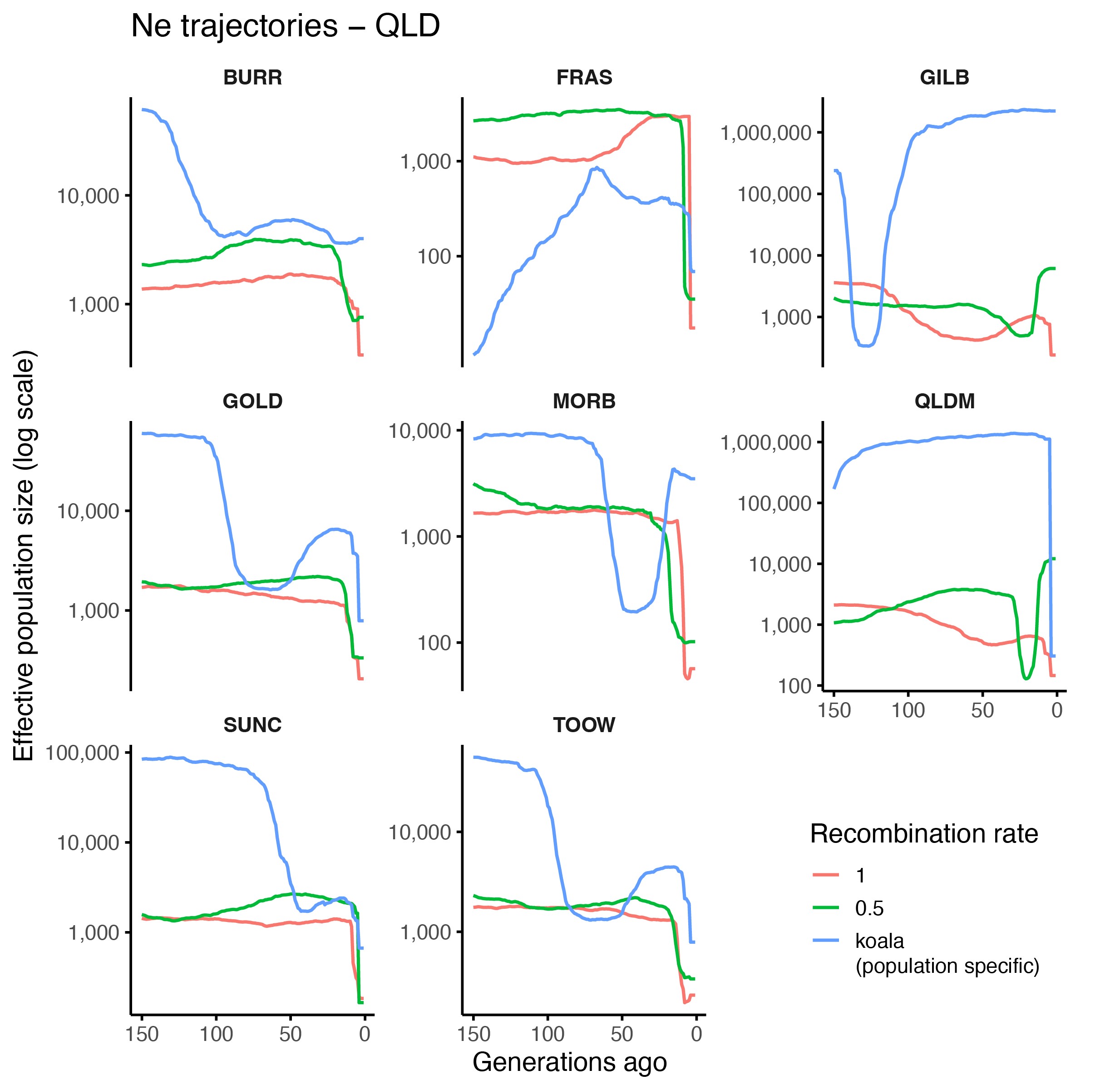


**Fig. S6**


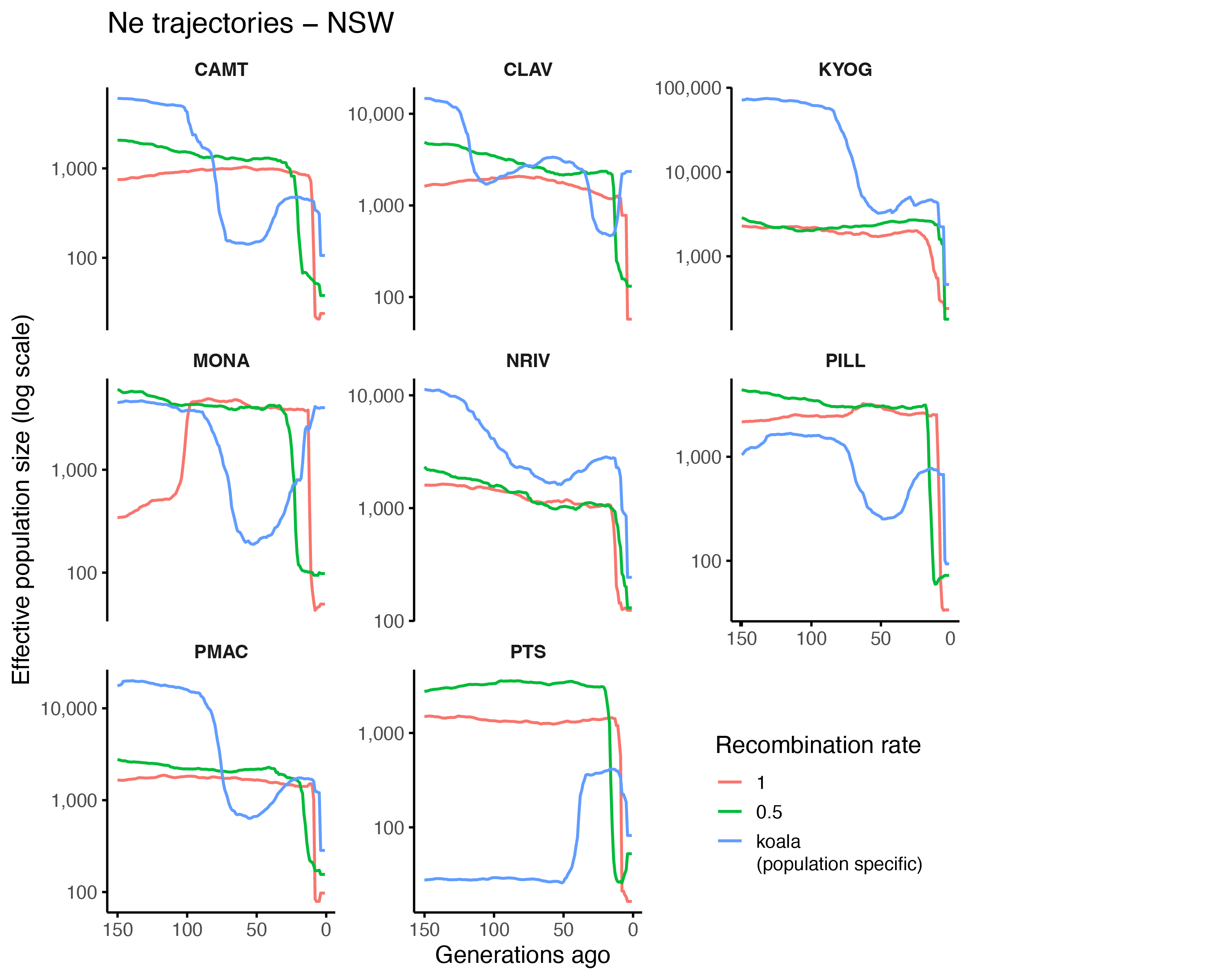


**Fig. S6 continued**


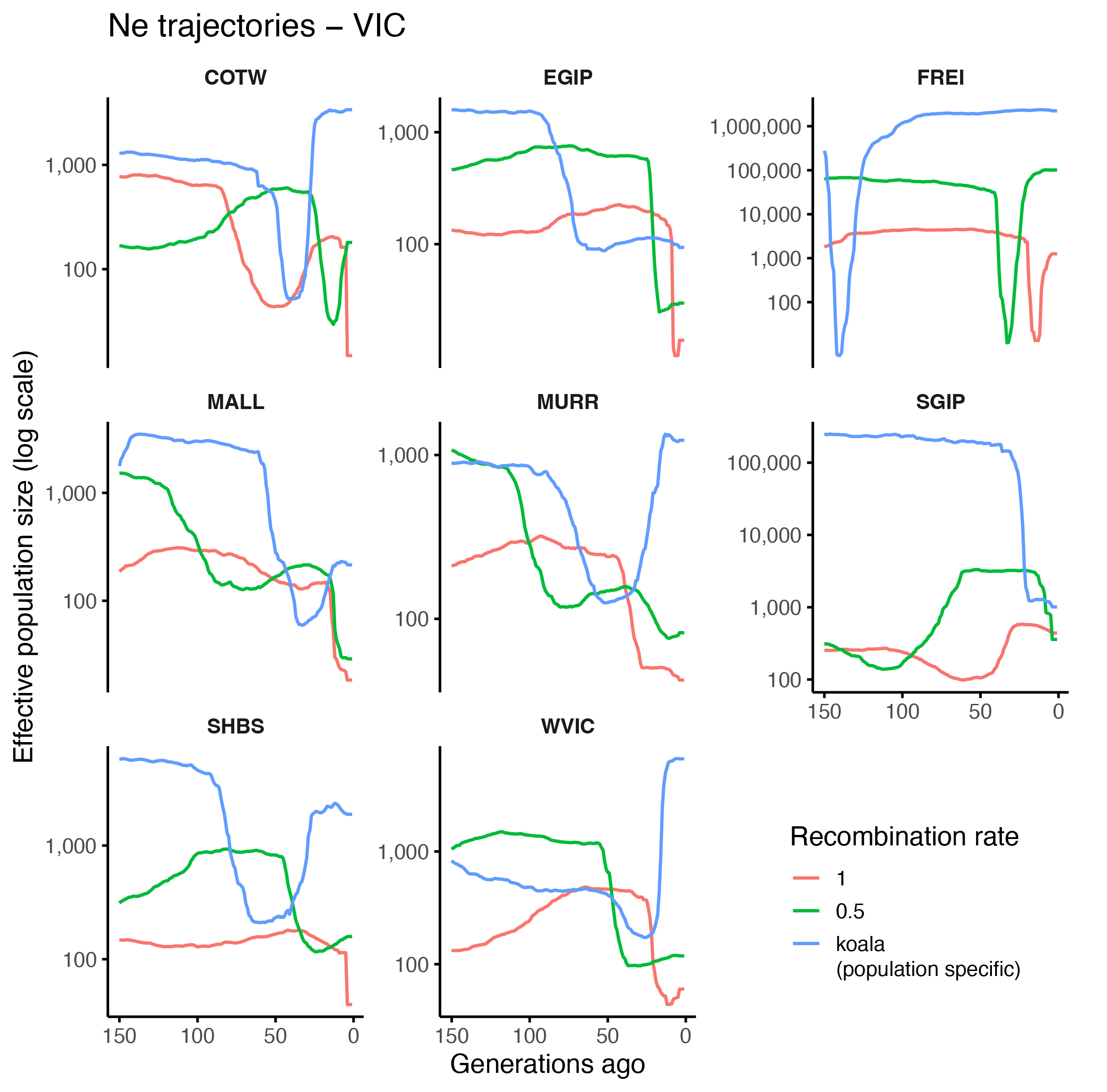


**Fig. S6 continued**
